# Success in vaccination programming through community health workers: a qualitative analysis of interviews and focus group discussions from Nepal, Senegal and Zambia

**DOI:** 10.1136/bmjopen-2023-079358

**Published:** 2024-04-03

**Authors:** Emily A Ogutu, Anna S Ellis, Kyra A Hester, Katie Rodriguez, Zoe Sakas, Chandni Jaishwal, Chenmua Yang, Sameer Dixit, Anindya Sekhar Bose, Moussa Sarr, William Kilembe, Robert Bednarczyk, Matthew C Freeman

**Affiliations:** 1 Department of Environmental Health, Emory University, Atlanta, Georgia, USA; 2 Centre for Molecular Dynamics, Kathmandu, Nepal; 3 WHO, Geneva, Switzerland; 4 Institut de Recherche en Santé, de Surveillance Epidemiologique et de Formations, Dakar, Senegal; 5 Center for Family Health Research, Lusaka, Zambia; 6 Hubert Department of Global Health, Emory University, Atlanta, Georgia, USA

**Keywords:** health education, public health, community child health, primary health care, immunization programs, capacity building

## Abstract

**Abstract:**

**Objectives:**

Community health workers are essential to front-line health outreach throughout low-income and middle-income countries, including programming for early childhood immunisation. Understanding how community health workers are engaged for successful early childhood vaccination among countries who showed success in immunisation coverage would support evidence-based policy guidance across contexts.

**Design:**

We employed a multiple case study design using qualitative research methods.

**Setting:**

We conducted research in Nepal, Senegal and Zambia.

**Participants:**

We conducted 207 interviews and 71 focus group discussions with 678 participants at the national, regional, district, health facility and community levels of the health systems of Nepal, Senegal and Zambia, from October 2019 to April 2021. We used thematic analysis to investigate contributing factors of community health worker programming that supported early childhood immunisation within each country and across contexts.

**Results:**

Implementation of vaccination programming relied principally on the (1) organisation, (2) motivation and (3) trust of community health workers. Organisation was accomplished by expanding cadres of community health workers to carry out their roles and responsibilities related to vaccination. Motivation was supported by intrinsic and extrinsic incentives. Trust was expressed by communities due to community health worker respect and value placed on their work.

**Conclusion:**

Improvements in immunisation coverage was facilitated by community health worker organisation, motivation and trust. With the continued projection of health worker shortages, especially in low-income countries, community health workers bridged the equity gap in access to vaccination services by enabling wider reach to underserved populations. Although improvements in vaccination programming were seen in all three countries—including government commitment to addressing human resource deficits, training and remuneration; workload, inconsistency in compensation, training duration and scope, and supervision remain major challenges to immunisation programming. Health decision-makers should consider organisation, motivation and trust of community health workers to improve the implementation of immunisation programming.

**STRENGTHS AND LIMITATIONS OF THIS STUDY**

- This study involved different stakeholders in immunisation programming from the national, regional, health facility and community level, thus, gaining diverse perspectives and insights on immunisation.
- The research team was multidisciplinary, and our team ensured involvement of all stakeholders in the design, implementation and the dissemination of this project.
- The research tools focused on the factors that drove catalytic change and did not focus on interventions or policies that were unsuccessful.
- The understanding of historical events using qualitative research methods was challenging; interviewees focused on current experiences rather than discussions on historical factors.
- COVID-19 pandemic impacted data collection as the stakeholders shifted focus to COVID-19 response and participants prioritised their safety over data collection.

## Introduction

 Despite efforts to increase early childhood immunisation over the past 20 years, many countries continue to report excessive morbidity and mortality from vaccine-preventable diseases. Maintaining high vaccination coverage is of economic benefit by preventing early childhood disease, improving health outcomes and reducing expenditure on treatment.^1 2^ The Immunisation Agenda 2030 (IA2030) aims to maintain hard-won gains in immunisation by leaving no one behind at any stage of life through increasing equitable access and use of new and existing vaccines, and strengthening immunisation within primary healthcare and universal health coverage.^3^ Through this strategy, countries define their own targets and timelines to achieve IA2030 goals.^3^ Achieving this goal requires strategising to address multiple inefficiencies, including health system strengthening, infrastructural development and increased human resources.^4^

The 2030 targets for the Global Strategy for Human Resources for Health, developed for low-income and middle-income countries, are to create, fill and sustain at least 10 million additional jobs in the health and social care sectors to address unmet needs for the equitable and effective coverage of health services.^5^ The WHO estimates a projected shortage of 18 million health workers by 2030, mostly in low-income and lower-middle-income countries.^6^ Estimates include the need for 44.5 basic health workers (eg, doctors, nurses and midwives) per 10 000 population; however, only half of the WHO member states have that level at present, with Southeast Asia and sub-Saharan Africa having the greatest absolute shortage of health workers.^5^ Community health workers (CHWs) are community members who volunteer to serve as front-line healthcare workers in underserved communities to bridge the human resource deficit.^7^ Continued expansion of the numbers, functions, training and support of CHWs and provision of financial packages commensurate to their job demands and workload could contribute to bridging the human resource gap because of the feasibility of CHWs deployment and effectiveness.^8 9^

As front-line health workers, CHWs play a critical role in routine vaccination programming by generating demand for childhood vaccination; connecting community members to the formalised healthcare system; and working closely with and in communities, schools and religious facilities.^10 11^ A concurrent analysis of our research showed that CHWs played a crucial role in demand generation in Nepal, Senegal and Zambia.^12 13^ The WHO health policy guidelines for CHWs programme (2018) recommend the following: (1) community engagement in selection, (2) training, (3) remuneration and (4) supervision. Coupled with other strategies, this policy aims to increase the efficiency and productivity of CHWs towards service delivery.^9^ Yet, despite these recommendations, front-line health workers in many countries—the vast majority of which are women—remain unpaid and are provided minimal training and supervision.^14^ Evidence suggests that scaling up and sustaining CHW programmes requires effective programme design and management, including adequate training, supervision, motivation and funding, community acceptability, and support from political leaders and other healthcare providers.^4^

The purpose of this analysis was to identify factors of CHW programming that successfully contributed to vaccination initiatives in Nepal, Senegal and Zambia. We describe (1) CHW programming organisation—cadres, roles and responsibilities, (2) CHW motivation—recognition, tangible incentives, capacity building, empathy and compassion and (3) trust—positive social capital, mutual trust- through community engagement and knowledge sharing. We discuss how these factors have contributed to success in service delivery and increased routine immunisation coverage in the context of the three countries. Programme implementation strategies involving active engagement of CHWs in Nepal, Senegal and Zambia may offer insight into what are critical contributors to immunisation access and intent to vaccination.

## Methods

We applied a multiple case study design to explore critical success factors of vaccine delivery systems in three countries with high vaccination rates: Nepal, Senegal and Zambia.^15^ This analysis is nested within the Vaccine Delivery project within the Exemplars in Global Health programme, funded by the Bill & Melinda Gates Foundation (grant/award number OPP1195041) and supported by Gates Ventures.^15 16^ We employed a qualitative analysis to investigate factors that contributed to high and sustained vaccination coverage through key informant interviews (KIIs) and focus group discussions (FGDs) at the national, regional, district, health facility and community levels of the health system.

In this analysis, we identified three themes—organisation, motivation and trust as they relate to CHWs and how these contributed to increased immunisation coverage in Nepal, Senegal and Zambia. We provide operational definitions of the themes and some specific examples of the components as they emerged from literature and data (table 1). The protocol of the primary analysis, along with individual case studies of countries and a synthesis case study, is published separately.^12 15 17 18^

**Table 1:** Definitions of themes as derived from literature and data

Domain	Definition and examples
Organisation	Expansion of CHWs with different cadres, and roles and responsibilities; may be based on the scope of work, reimbursement, qualifications, language, education level, geographical region, and their distinct roles and responsibilities.^27 44^ These CHWs work together to improve community health and increase vaccination rates.
1.A. Cadre	Specialised types of CHW providing immunisation-related services in the communities Female community health volunteers, mother groups, village development committees (Nepal). Agents de Santé Communautaire, Matrones, Dispensateur de Soins à Domicile, Bajenu Gox, Relais (Senegal). Neighbourhood Health Committees, Safe Motherhood Action Groups, Growth Monitoring Promoters (Zambia).
1.B. Roles and responsibilities	Work and functions of CHWs related to immunisations activities, based on specific age ranges of the CHWs, ethnicities, language skills and qualifications, including: Defaulter tracing which involves finding children who missed their scheduled immunisation. Outreach planning. Data collection and reporting. Community mobilisation. Public awareness and education.
Motivation	The reason, desire (eg, empathy, compassion) or willingness of a CHW to do their work.^25 45 46^
2.A. Recognition	Identification of CHWs by the communities and healthcare workers as integral members of healthcare team due to their knowledge and close understanding of the communities they serve. Examples include: Respect by community members. Support from community. Value placed on the work of CHWs. National, regional or local celebrations.
2.B. Tangible incentives	Factors—direct or indirect, financial or non-financial—that increase motivation to engage and perform well, which include specific forms of reward.^35^ Examples include: Money, including salary and remuneration for specific training. Food, usually staples of diet. Childcare. Harvesting assistance. Bicycles, uniforms, raincoats, stationary. Certificates, including-training completion. Free healthcare services.
2.C. Capacity building	Includes training and supervision of CHWs and the downward impact on the community, including: Increased self-efficacy to perform responsibilities. Increased knowledge of diseases, diagnoses, treatment and facilitation. Contribution to programme planning and decision-making.
2.D. Empathy and compassion	The ability to understand and share the feelings of others, show concern for the suffering or misfortune of others, and prioritise the needs of other community members—all of which motivated CHWs to perform their roles.
Trust	Positive social capital or social relationship, as an element of truth telling, empowering others through knowledge.
3.A. Community engagement	The involvement of communities in decision-making and in planning, design, governance and delivery of services.^47^ Community members involvement in selection of CHWs. CHW as residents of their respective communities. CHWs speak the same language as community members. CHWs relaying health information to community members and sharing feedback from the community to healthcare workers.
3.B. Knowledge sharing	Activities through which information and skills are shared between individuals or groups can include frequent interaction, social relationships between CHWs, communities and health workers.^48^

CHW, community health worker.

### Study settings

**Figure 1:**
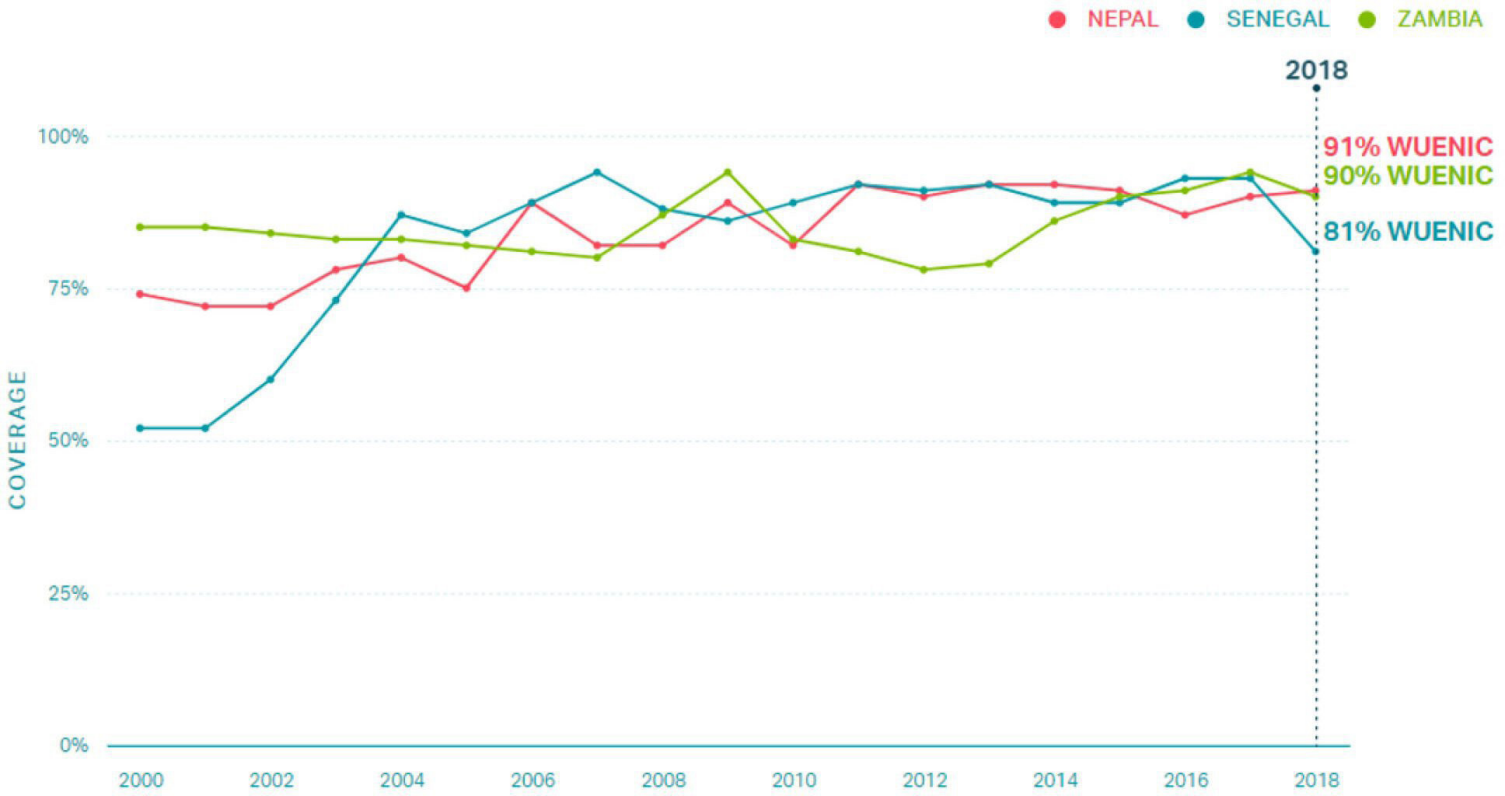
DTP3 coverage of Zambia, Nepal and Senegal from 2000 to 2018; WUENIC data. DTP, diphtheria, tetanus, pertussis.

Nepal, Senegal and Zambia were selected based on their success at achieving high and sustained growth in early childhood vaccination, based on historical (ie, 2000–2018, up to the point of starting this project) diphtheria, tetanus, pertussis vaccine (DTP) 1 and DTP3 coverage estimates. Figure 1 provides snapshot of coverage estimates for Nepal, Senegal and Zambia. Details are explained elsewhere.^15^ Three regions within each country were identified in consultation with national stakeholders and available data.^12 17 18^ Ministry of Health (MoH) officials and in-country partner organisations facilitated site selection and data collection activities. Each country has a slightly different name for their MoH (eg, MoH and Population in Nepal; MoH and Social Action in Senegal), but all will be referred to as ‘MoH’ throughout for simplicity.

### Qualitative data collection and analysis

#### Qualitative data collection

Qualitative data were collected at the national, regional, district, health facility and community levels. Data collection was from August 2019 to April 2021. The interview guides were informed by the Consolidated Framework for Implementation Research and the Context and Implementation of Complex Interventions frameworks.^19 20^ KII and FGD guides were translated into national and regional languages by research assistants, piloted by the research assistants among themselves before use and adjusted iteratively throughout data collection. An initial list of KIIs was developed with local research partners and MoH officials; snowball sampling was used to identify additional key informants. Sample size was determined by the point of saturation, when in-depth understanding of issues from the interviews was gained, and no new information was noted from the KIIs and FGDs.^21^ Caregivers and CHWs were recruited for FGDs from health facility catchment areas with the assistance of local health staff. The duration of KIIs and FGDs averaged one and a half hours. KIIs and FGDs were conducted in Nepali in Nepal, French in Senegal and Bemba in Zambia. KIIs and FGDs were audiorecorded with the permission of participants. Transcripts were translated to English manually or by using Google Translate (for French) and verified by fluent bilingual speaker. Research files, recordings and transcriptions were deidentified, password protected and kept on a secure server. The activities are summarised in table 2.

**Table 2:** Summary of countries, partner organisations, regions, districts and data collection activities^49^

Country	Nepal		Senegal		Zambia	
In country research partner	Centre for molecular dynamics, Nepal		Institut de Recherche en Santé de Surveillance, de Surveillance Epidémiologique et de Formation		Centre for Family Health Research in Zambia	
Data collection period (MM/YYYY)	August/2019–December/2019		December 2020–April 2021		October 2019–February 2020	
Regions and districts						
Region 1	Madhes*	Dhanusha, Bara, Mahottari	Ziguinchor	Ziguinchor, Oussouye, Diouloulou	Lusaka	Lusaka, Rufunsa, Chongwe
Region 2	Bagmati	Makwanpur, Dolakha, Kathmandu	Dakar	Rufisque, Mbao, Keur Massar	Central	Chibombo, Chitambo, Serenje
Region 3	Gandaki Pradesh	Kaski, Myagdi, Nawalparasi	Tambacounda	Tambacounda, Koumpentoum, Goudiry	Luapula	Chipili, Nchelenge, Samfya
KII: Number of KIIs (participants)	**79** (**79**)		**62** (**63**)		**66** (**85**)	
National-level government staff	11 (11)		5 (5)		11 (12)	
Partner organisation staff	8 (8)		4 (4)		11 (15)	
Regional health staff	5 (5)		7 (7)		6 (8)	
District health staff	15 (15)		38 (38)		10 (19)	
Health facility staff	23 (23)		6 (6)		7 (10)	
Community leaders	15 (15)		2 (2)		10 (10)	
CHWs†	2 (2)		–		11 (11)	
FGD: Number of FGDs (participants)	**30** (**191**)		**19** (**128**)		**22** (**132**)	
CHWs†	9 (60)		10 (65)		10 (60)	
Mothers	9 (60)		9 (63)		8 (48)	
Fathers	6 (36)		–		1 (6)	
Grandparents	6 (35)		–		3 (18)	
Total (per country)	**109** (**270**)		**81** (**191**)		**88** (**217**)	
Total (across countries)	–		–		**278** (**678**)	

Total number of KII activities and participants per country

Total nummber of FGD activities and participants per country

Total number of KIIs and FGD activities and participants per country

Total number of KII and FGD activities, and participants across the three countries

* Madhes was referred to as province 2 prior to 2022 (including data collection period).

† Includes FCHV in Nepal, vaccinators, bajenu gox, Agents de Santé Communautaire in Senegal and CHWs and neighbourhood health committee members in Zambia.

CHWs, community health workers; FCHV, female community health volunteer; FGDs, focus group discussions; KIIs, key informant interviews.

#### Data analysis

We applied thematic analysis to identify the contribution of CHWs in the success of childhood immunisation in Nepal, Senegal and Zambia. Emory researchers developed codebook inductively using emerging themes, and deductively using themes from CHW frameworks from previous studies, coded the transcripts and analysed data using MaxQDA2020 software (Berlin, Germany). The coding team met frequently, discussed emerging themes and clarified discrepancies within the codes. The researchers then categorised the themes within the broader domains of organisation, motivation and trust (table 1). Topic guides and codebooks used for data collection and analysis are attached in the (online supplemental appendix 1^22^ and online supplemental appendix 2. 

### Patient and public involvement

All stakeholders in the immunisation sector from global, national, regional and the community level were involved in this study. Researchers, policy-makers and other actors in the immunisation sector were involved in the design, implementation and dissemination of this project in Nepal, Senegal and Zambia. We conducted a scoping visit in these countries and engaged with the in-country partners and experts to expand on, and get more guidance on the initial questions developed by our technical advisory group (TAG). The final data collection tools were informed by the findings from the scoping visits. We followed best practices for qualitative research, and iteratively incorporated feedback from experts, TAG and the local partners. Communities were involved through FGDs and interviews, with the primary focus of data collection being centred on historical stakeholders who played key roles in the immunisation programme. We gathered feedback on the methods, findings and implications of the findings through engagement in discussions with advisors, follow-up emails and phone calls with selected individuals, which lead to improvements in data analysis and presentation. We disseminated final findings to key stakeholders including in-country experts, country governments and TAG for validation and contextualisation. Reports, and manuscripts, of findings were sent to those involved in each country. More information on how local research and policy priorities were addressed is included in the reflexivity statement (online supplemental appendix 3).

### Findings

**Figure 2:**
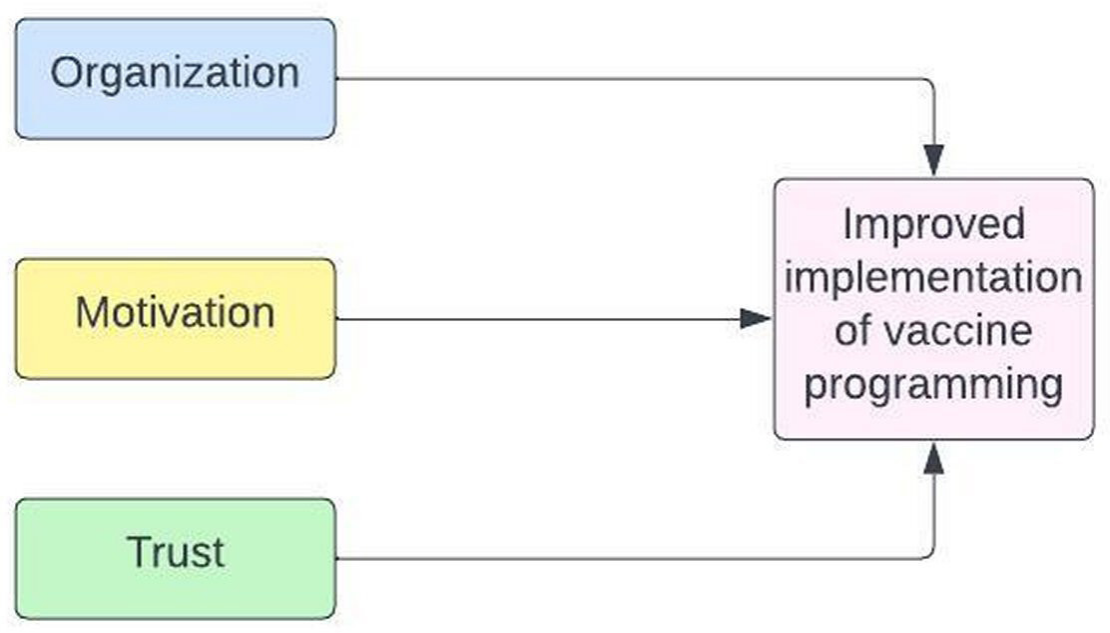
Summary figure of CHW domains that shaped vaccination in Nepal, Senegal and Zambia. CHW, community health worker.

We analysed strategies and delivery methods of CHWs and how they contributed to routine vaccination programming in Nepal, Senegal and Zambia. First, we developed a framework specific to CHWs and their involvement in vaccine delivery and uptake to facilitate organisation of the findings. We developed the framework both deductively and inductively, withdrawing the domains of organisation, motivation and trust from previous studies, and aligning the definitions and categorisations with information from data. We included aspects of our general research framework and other scales and frameworks specific to CHWs.^23 24^ We found that CHWs’ contribution to improvement in healthcare delivery and utilisation in different settings aligned with the broad domains around cultural congruence, social support, mutual trust and others.^23^,^25^ We then focused on the themes of organisation, trust and motivation (figure 2) to compare success factors across these countries. For this analysis, we chose to focus on the roles of CHWs, recognising that in all countries, at least one of the cadres (eg, bajenu gox) was more trusted by communities than other CHW cadres, and was instrumental in the vaccination programming (table 3). CHWs capitalised on their strong community role to educate community members, combat misinformation, conduct outreach and provide personalised service to caregivers. Themes around (1) organisation, (2) motivation and (3) trust were derived from the literature and aligned with data, and these contributed to improvements in vaccination programme implementation (table 1). Organisation through expansion of CHW types motivated CHWs to carry out their roles and responsibilities related to vaccination. Trust that communities and healthcare workers expressed to CHWs through respect and value placed on work contributed to CHWs’ motivation resulting in the improved implementation of vaccination programming.

**Table 3:** Community health workers cadres, roles, recruitment requirements, training and remuneration by country

	Cadre title	Established	Roles and responsibilities	Key requirements*	Reported training	Remuneration†
Nepal	FCHVs	Initiated in 1988, played critical role since 1991, in 75 districts, 52 000 FCHVs in the country.^50^	Treatment of common illnesses, maternal and child health interventions Support national campaigns Defaulter tracing Lead mother health group meetings and disseminate health information Report data on births and vaccinations	Selected by mother health groups Married and reside in the area of operation Literate No other income-generating activities	Initial training (18 days) Refresher training yearly	Receive 10 000 rupees from the government on retirement
Senegal	Agents de santé communautaires (ASCs)^51^	Since the 1950s	Maternal and child health interventions and services Education on vaccines and referrals Support outreach strategies Supervised by nurse in charge	17–47 years of age Read and write in French Speak local language From local community	Initial training (30+ days, 10 days practice)	Determined by Comités de développement sanitaire or non governmental organizations
	Matrones^51^	Not clear when this was established	Maternal and child health services and interventions Education on vaccines and referrals Support outreach strategies Supervised by nurse in charge	Age: 25–50 years Education: at least fourth grade Reside in the community	Initial training (30+ days, 10 days practice)	Determined by CDS or NGO
	Home-based care providers (Dispensateur de Soins à Domicile)	Created in 2008^51^	Integrated management of childhood illnesses in remote communities Referrals of clients to ASCs Supervised by nurse in charge	Able to read Selected by local community	Initial training (30+ days, 10 days practice)	Determined by CDS or NGO
	Bajenu Gox	Launched in 2009^52^	Promote maternal and child health interventions including vaccination Outreach services: coordination and attendance Door-to-door activities, defaulter tracing and referrals of clients Supervised by non governmental organization employees, community development agents and head nurse	Proven leader Female From and selected by local community	Initial training (40–55 days)	Determined by CDS or NGO
	Relais communautaires	Not clear when this was established	Education and behaviour change strategies: Maternal neonatal child health, integrated management of childhood illnesses topics, water, sanitation and hygiene Outreach services: coordination and attendance Door-to-door services, defaulter tracing and referrals of clients to ASCs Text reminders to parents of vaccine days Supervised by nurse in charge	From the local community	Initial training (40–55 days)	Determined by CDS or NGO
Zambia	Neighbourhood health committees (NHCs)	Introduced in the late 1990s	Review reports Identify and address barriers Hold monthly meetings with village leaders Provide education on vaccines Follow-up visits, conduct head count children aged 0–11 months and 1–5 years	Influential community member Community appointed	Receive 1 week training from health centre nurse in charge Can be both CHW and NHC	Volunteer
	Safe motherhood action groups	Established in 2003^53^	Education on prenatal, postnatal and infant immunisations Refer women with complications to health facilities Persuade husbands to support their wives during pregnancy and accompany them for ante-natal care visits	Community appointed based on respect, trust and acceptability by community	Standard 5 days training^54^	Volunteer
	Growth monitoring promoters	Introduced in the 1980s^55^	Education on nutrition for children aged 0–5 years Promotes childhood immunisations	From the local community	4 days training; retrained every 6 months	Volunteer

* All cadres had to be willing to volunteer and selected by the local community.

† Renumeration was dependent on the district, CDS (Senegal), or external partner managing each cadre, and not based on the cadre itself.

FCHVs, female community health volunteers.

### Organisation

In all three countries, key informants reported that recently introduced cadres and diversification of roles and responsibilities contributed to improved implementation of vaccination programming. The roles and responsibilities of CHWs shifted and often expanded, and this was subsequent with increased funding and focus on health services strengthening. Governments created more positions/cadres, defined and aligned responsibilities with specific qualifications, which bolstered front-line health outreach and services (table 3). The current CHW roles across the three countries required specific qualifications, training and responsibilities. CHW roles included generating public awareness, dispelling misinformation, defaulter tracking (for beneficiaries missing immunisation) and data collection.

#### Cadre

Each country had different types of CHWs who played specific roles in immunisation programming. These cadres were created to distribute the heavy workload, assign specific roles and responsibilities to specialised staff and improve overall coordination and delivery of services. In Nepal, female community health volunteers (FCHVs) had a major role in immunisation, they created awareness on immunisation and linked the communities to the healthcare facilities. Senegal had five different cadres (table 3) involved in different aspects of immunisation. In Zambia, neighbourhood health committees (NHCs), safe motherhood action groups and growth monitoring promoters played a critical role in improving vaccination coverage through the roles and responsibilities they held as discussed in the next section.

The specific characteristics and detailed elaboration of each cadre of the CHWs in Nepal, Senegal and Zambia are outlined in table 3.

#### Roles and responsibilities

In the three countries, at least one CHW cadre existed. In Senegal and Zambia, most of CHWs performed similar roles. The lack of defined roles and localised training led to overlapping or deficient coverage of health services by the cadres in Zambia and Senegal.

In Nepal, key informants indicated that FCHVs, ‘the backbone of the health system’, received the most credit nationwide for improved immunisation coverage. With the increase of paid front-line health workers in 2010, the responsibilities of FCHVs shifted to building awareness of available health services and their benefits at different community levels, including local mothers’ groups (table 3).

FCHVs and the mothers’ group provide information regarding immunization campaigns or [the] regular immunization program. For new vaccines, it needs to [be] discussed with FCHVs and [the] mothers’ group—unless there is support and involvement in the program, it will not be successful because they are the one who build awareness and give knowledge and information to the people in the grass root level. (Ministry official, National level, Nepal)

In Senegal, relais and bajenu gox conduct more targeted vaccine outreach although the five cadres of CHWs serve complementary roles in delivering front-line health services (table 3). KIIs and the literature identified the relais and the bajenu gox as critical to the success of the vaccination programme, and mothers referred to them as ‘real fighters’ for the health of children. Community promotion actors were mobile, focusing on visiting communities to promote awareness of and create demand for disease prevention and health promotion activities, including vaccinations. Community members lauded the mobilisation and the motivation these actors inspired which contributed to the improvement of vaccination programming.

The involvement of community actors helps them a lot because the community actors live with the populations and know the families. Today, even after the counting, they cannot determine the house of someone or they are given the wrong telephone number. But with the involvement of community actors, they go into the neighborhoods to bring back the children. (Community health worker, community level, Senegal)

In Zambia, key informants discussed the NHCs extensively. NHCs are community-appointed volunteers whose primary role is to coordinate and manage other CHWs on behalf of health facility staff, including CHW recruitment, training and attrition. NHCs also ensure that CHWs accurately report monthly activities and represent both CHWs and community members in annual action planning. NHCs’ responsibilities have increased in the last two decades to accommodate the increasing number of CHW roles across multiple health sectors, bridge the gap in human resources and institutionalise the link between communities and the health system. Many individuals who hold NHCs role also hold another CHW position so as to receive training and other incentives through disease-specific initiatives.

A long time ago, as I said, we [community members] used to come in numbers here at the [health] center. It was difficult because we [the community] did not have enough [health facility] staff, so that’s how this [the NHC] program started. And they [NHCs] started talking to people so that we [NHCs] help each other because members of staff were few. We [NHCs] are grateful they trained us, and we are working. (NHC member, community level, Zambia)

The NHCs in Zambia were involved in action planning, identifying annual priorities, setting objectives, selecting strategies, determining indicators to monitor and evaluate activities, and estimating required activity resources and budget. Through their involvement, the NHCs indicated ownership over these responsibilities, which were formalised in the Reaching Every District (RED) programme manual.

### Motivation

CHWs in all three countries emphasised the meaning and value they found in their work. This was through recognition of their effort, incentives and knowledge gained through training. CHWs received training which increased their knowledge on vaccinations and the present vaccine challenges. However, training varied and was sometimes inconsistent within specific countries. Supervision was done to ensure that CHWs performed their roles well; though, inconsistency in supervision was experienced in all countries, with key informants indicating workload and insufficient human resources as the barriers. Beyond the individual level, CHWs were motivated through the declaration and celebration of their villages by the national government as fully immunised (Full Immunisation Declaration (FID) Programme in Nepal), and successfully reaching all children in their villages through the implementation of Reaching Every Child (REC in Senegal) and (RED in Zambia).

#### Recognition

In all three countries, CHWs stated that recognition for their contributions and their status in the community were motivating factors. Communities, districts and regions recognised the efforts and successes of CHWs through ceremonies, certificates and media recognition. Community selection showed recognition and respect for the individual chosen to be a CHW. Particularly for women, CHWs gained stature through imparting valued knowledge, supporting caregivers and contributing to positive health outcomes.

Our motivation is we have respect through this work. Everyone praises our work. They praise our contribution towards work for pregnant women, children and delivery of pregnant women. (FCHV, Nepal)

In Nepal, FCHVs appreciated the recognition for their role in increasing vaccine coverage. Some FCHVs received certificates. Villages, districts and provinces were declared fully immunised and celebrated by the national government through the FID Programme in a bottom-up approach, and this motivated community workers and volunteers, and facilitated community buy-in to immunisation. Children who are fully vaccinated receive an official vaccination card with complete information on vaccines received from their health post for documentation. Increased public awareness and FCHV activities support the success of the Full Immunisation Programme (FIP)—which promoted urgency and action among all key players, including caregivers, religious and community leaders, and teachers.

We are all working together so that the children won’t miss vaccines. We can assure you that every child in our area have got vaccines. We celebrated the immunization month at [location] last time. (FC*HV, Nepal)*

In Nepal and Senegal, CHWs expressed their motivation from the satisfaction they derived from belonging to their communities. CHWs highly respected their communities, and they served voluntarily because of the mutual respect between community and CHW. Seeing children in their communities protected from diseases and growing up healthy was additional motivation. FCHVs had pride and expressed happiness if all the children within their catchment areas were fully vaccinated. A FCHV in Nepal stated that ‘they worked for the good of the community.’

#### Incentives

In all countries, incentives (table 1) were crucial to engage and motivate CHW volunteers. Though inconsistently distributed, tangible extrinsic resources—including money, food, bicycles, uniforms, stationary, childcare and harvesting assistance—motivated CHWs and could reduce any intrahousehold tensions caused by absences due to the programme. When CHWs areas of jurisdiction reported low vaccination coverage, especially in comparison to other districts or regions, their motivation to improve outcomes increased.

We do recognize them [FCHVs]. We do a public audit and take decisions on whom we should give credit; that is recognized by the community and system so that they become more motivated. (Regional Director, Nepal)

However, even with efforts to recognise CHW contributions, CHWs in all countries felt underappreciated by the national government. They expressed that the appreciation they received was more evident in the community than by the government. In all countries, CHWs felt they lacked commensurate compensation for their labour; they were required to commit to being a volunteer. Retention suffered because in some instances, CHWs were forbidden to engage in income-generating activities, and compensation was often inconsistent. CHWs families often expected them to complete household labour even though they were engaged in volunteer responsibilities. Community health volunteers often supported and contributed to the work of paid CHWs and head nurses, resulting in additional workloads without appropriate compensation. Additionally, countries’ projections for labour were unmet, leading to heavy responsibilities for CHWs.

#### Capacity building

CHW’s knowledge increased through training and supportive supervision; in many cases, the opportunity to learn new skills and knowledge promoted self-value and enhanced their capacity to support the communities in the three countries. CHWs received formalised training through national health programmes, Non-governmnetal organizations (NGOs) and multilateral partners; however, the length, breadth and depth of training differed by country and trainer (table 3). Key informants valued NGO and multilateral training, which provided financial incentives. Some FCHVs and bajenu gox reported the benefits of CHW literacy promoting groups. In all countries, CHW used their community knowledge through identification of community needs around immunisation, which informed decision-making, and ultimately contributed to planning at higher levels. CHWs had frequent interactions with the local population, as they resided in those communities. This facilitated their deeper understanding of the community’s needs, which they escalated during planning meetings. The frequent interactions between CHWs and community members through community dialogues and education, and encouragement of knowledge sharing between the community members themselves enhanced community empowerment, enabling development and maintenance of social norms related to vaccination.

Supervision of CHWs occurred in all countries. Supportive supervision increased CHWs self-confidence through cross-learning and skills sharing facilitated teamwork, and enhanced relationships with healthcare workers. In Senegal, bajenu gox received supportive supervision while working in communities. Their supervisors recorded field visit results on a standardised checklist and sent supervision reports to the district medical offices for review. However, supervision in the field may have been rare due to a lack of human resources. In Zambia, the NHCs had a supervisory position over CHW reporting, and played an essential role in data collection and evidence-based decision-making. Prior to taking up their roles, NHCs received training, which enhanced their capacity to perform their duties. NHCs were motivated by these responsibilities because they elevated the NHC’s position and described the emphasis on community-driven strategies to combat health challenges. Health facility staff and supervisors from the districts included NHCs in meetings and trainings, which provided encouragement. According to national policy, by encouraging the possession and responsibility of data, the front-line workers reviewed their performance against their stated goal, identified gaps and discussed ways to improve vaccination coverage.

There is the NHC that spearheads all community activities because all the CBVs, all community volunteers, they report through the NHC. (District Nursing Officer, Zambia)

Although CHWs in all countries desired consistent and increased opportunities for training; these differed significantly depending on the location and human, financial, and material resources.

#### Empathy and compassion

CHWs expressed being motivated by their sense of empathy and compassion for their communities in all three countries. CHWs understood and shared the feelings of their fellow community members and showed concern for their suffering and misfortune. This motivated CHWs to perform their roles well since they felt invested in the health of the community at large in addition to children’s health. CHWs understood the lived experiences of the community members and had compassion for the potential morbidity and mortality of children when not immunised against vaccine-preventable diseases. This increased their effort to focus on improving the health and well-being of children in their communities.

Vaccine prevents from many diseases that is also why people bring their children for vaccination. There were more Pneumonia cases and diarrheal diseases before but nowadays there are very less children who suffer from Pneumonia and Diarrhea. If we hear that some children have suffered from diseases, then we feel very worried. We also feel responsible towards those children. (FCHV, Nepal)

### Trust

CHWs increased community members’ trust in the formalised health system. CHWs were respected members of the community, with some individuals regarded as opinion leaders; this trust was facilitated by the selection of CHWs from the communities in which they lived. CHWs connected with the community by promoting and prompting vaccination. In Senegal and Zambia, CHWs facilitated the adaptation of REC/RED strategy to local context, ensuring community buy-in. These strategies required frequent interaction with community members—providing education, action planning, conducting surveys and tracking vaccination records. Through such interactions, social relationships were built, which strengthened community trust in CHWs and the activities they carried out within these communities. Although all CHW cadres had roles they served towards improving the health and well-being of the communities, mothers identified a cadre of CHW that they most trusted and felt they had the most investment in their health (mothers) and their children’s health (table 3).

#### Community engagement and knowledge sharing

Communities in all three countries were involved in decision making, planning, design, governance and delivery of services. Information and skills were shared between individuals or groups, and this included frequent interaction, and social relationships between CHWs, communities and health workers. CHWs were involved in providing health education at facilities, thus creating awareness about immunisation.

FCHV sisters also help us and teach us about [health]. They also teach and train us to get vaccination for the children… We trust [the information] and it is for our children. It is better not to suffer from any diseases. First, we need to have proper information about it and only then trust it. (Female Community Health Volunteer, Nepal)

In Nepal, FCHVs were required to work in the communities they resided in; CHWs reported that this generated trust and promoted culturally and contextually appropriate messaging to diverse stakeholders. Health and immunisation education were presented in monthly meetings; content was selected and tailored depending on literacy, misinformation and cultural norms. FCHVs educated community members in mothers’ health groups, ensured the group’s integration into their assigned communities to share health information with community members. In women’s savings groups (groups of women who come together to save, and lend, money to members), FCHVs emphasised the link between children’s health and future educational and occupational opportunities. FCHVs also approached caregivers individually, discussing potential barriers to immunisation, addressing specific information caregivers lacked and encouraging immunisation access. As FCHVs were embedded in the communities where they conducted outreach, their messaging incorporated differences in religion, tribe or caste, ethnicity and language.

I: How much do community members trust the sources of Vaccination information? //P4: Yes, they have trust. //All at a time: yes, we believe on them. //P5: Because they will also learn some information from here and provides us other information. // P4: They will learn from here and teach us. (Mothers, Nepal)

In Senegal, the bajenu gox included multiple family members in educational outreach on vaccinations and maternal and child health. Bajenu gox held education groups for adolescent girls in addition to women of all ages to target misinformation and mediate between different age groups. In recent years, bajenu gox has also conducted outreach with fathers’ groups, aiding in full family approval of childhood vaccinations. To address the lack of necessary human resources for community health outreach and support in Senegal, the MoH, under a USAID-funded community health plan, instituted the bajenu gox (godmothers) programme in 2009. The bajenu gox was trusted older women—‘proven female leaders’—living in the community who capitalised on their age and social role to support pregnant women and their families in prenatal to postnatal health, including immunisations (table 3). Bajenu gox duties were specifically targeted at building relationships of trust with community members. Many mothers specifically praised the bajenu gox for taking the time to explain diseases that vaccinations targeted, when vaccinations were happening, follow-up and individualised planning.

Special mention to our bajenu gox. We are all women, and we see the support they give us; they are our mothers and at the same time our bajenu gox. They leave all their activities to follow us and our children…each time there is a vaccination to be given; they identify the people concerned and accompany them to be vaccinated. Because vaccination is very important, before we said that without it, we could catch all kinds of diseases such as measles and others, but now we have more vaccines and thank God we have fewer diseases, and all our children are protected. (Mothers, Senegal)

Trust between the health workers and the bajenu gox was enhanced through supportive supervision. NGO employees, community development agents and head nurses were intended to provide supportive supervision for bajenu gox while they were working in communities, record field visit results on a standardised checklist, and send supervision reports to the district medical offices for review. This allowed for confidence and trust with service delivery among bajenu gox, the communities and healthcare workers.

In Zambia, NHCs played a prominent role in community sensitisation, outreach and vaccine education in most of our research communities in Zambia. NHCs were a trusted source of information for community members since they were recruited by community leaders and worked closely with health facility staff to coordinate outreach sessions. Consistent follow-through of outreach sessions contributed to community members’ trust in NHCs and, by extension, the health system. NHCs also worked to dispel myths and misconceptions about vaccinations by educating parents on the benefits. For example, if the barrier to access were due to a parent’s competing priorities, the NHC member would escort the child to the outreach event.

There is some time on the radio, and they call the NHC to come and talk about health issues. They encourage, especially in the village. They announce that next week, 'do not delay the children to come for vaccinations.' Yes, because of the community radio, they let anyone talk. Yes, especially the NHC, they talk a lot on encouraging other vaccines. (Head Woman, Zambia)

The engagement of CHWs and their communities and the frequent interactions CHWs had with their communities, especially with mothers, facilitated the understanding of the social and cultural norms which facilitated knowledge sharing and enhanced trust between the CHWs and the communities they served and the information they shared.

## Discussion

We conducted a qualitative study to assess factors of the CHW programmes that contributed to success in implementation of vaccination initiatives in three countries between 2000 and 2019—Nepal, Senegal and Zambia. Our findings suggest three domains behind CHWs—organisation, motivation and trust—strongly contributed to improve the implementation of health programming and may have positively influenced vaccination during our targeted time period. Organisation through expansion of CHW cadres motivated CHWs to carry out their roles and responsibilities related to vaccination—resulting in the improved implementation of vaccination programming. Our findings are policy relevant for other countries looking to expand vaccination through CHW platforms; however, those looking to apply these findings should be mindful that adaptation to local context is crucial.

### Organisation

Distinct cadres of CHWs had specific roles and responsibilities assigned to them in the three countries, which aligned with strategies implemented in other countries.^26^ This study, however, pointed out insufficiency in CHWs supervision, like findings from other studies within similar contexts.^4 27^ Expansion of CHW cadres increased the need for supervision. Since CHWs in middle-income and low-income countries are not healthcare professionals, increased supportive supervision may increase and enhance retention of knowledge, thus increasing their confidence. CHWs working together with other health workers and receiving required supervision could increase their confidence in work, and their motivation to work harder, thus improving their performance.

### Motivation

Motivation of CHWs was increased through intrinsic ‘work for the good of the community’ and extrinsic incentives—certificates, recognition, stipends and trust. Social cohesion—the ‘capacity of society to ensure well-being of all its members by minimising disparities and avoiding marginalisation’ in regard to the health and well-being of children was seen in the three countries.^28^ Capacity building, public awareness, involvement of CHWs and communities in different health programmes, and the recognition of childhood vaccination as a human right enhanced a generational shift in existing norms—hence communities increasingly embracing vaccination.^12^ In Asia and Africa, a multiple case study found that extrinsic motivation improved CHW work, similar to the findings of this study.^29^ Organisation and cultural context shaped the motivation of CHWs, as individual beliefs, pleasure and happiness drawn from well-being of children in the communities was also motivating.^29^ In Zambia, safe motherhood action groups expressed happiness with their work because they helped saved women’s lives.^30^ In Nepal, Senegal and Zambia, CHWs were part of the communities they served, increasing their sense of belonging and trust, therefore, increasing their motivation to dispense their duties.

CHWs are often the backbone of the front-line health systems in many parts of the world. Yet, this workforce, most of whom are women, remains unpaid, often resulting in undue psychosocial stress, food insecurity and time burdens.^31 32^ Unpaid work, including the roles of female CHWs, leave CHWs time poor, being unable to meet their basic needs, or participate in social or political activities—contributing to the widening equity gap and expanding the poverty cycle.^33^ CHWs’ great contributions to healthcare delivery is evident; however, limited attention has been placed on their remuneration. Nevertheless, payment of living wages to CHWs is a fundamental right.^34^ WHO now recommends remunerating practicing CHWs for their work with financial package commensurate with job demands, complexity, number of hours, training and roles and responsibilities.^9^ Nepal, Senegal and Zambia provide incentives to CHWs; however, this is incommensurate with their work.^35^ Similarly, countries such as Sierra Leone and Liberia have policies that guide the remuneration of CHWs, but CHWs from these countries expressed dissatisfaction with remunerations in place, highlighting the need to consider contextual factors and roles and responsibilities when planning and budgeting for CHW remuneration.^36^ Although consistency in the incentivisation of FCHVs was observed due to integration, and the financing of CHW programming at the national level in Nepal, Senegal and Zambia reported inconsistencies. In low-income and lower-middle-income countries, CHWs are not adequately remunerated, even with long service to the community and numerous contributions to elimination of diseases, reducing the menace of diseases, and increasing the reach of immunisation programmes. Furthermore, the roles of CHWs are deemed to fit into the other domestic activities, which are mostly done by women.^37^

Nevertheless, the incentivisation of CHWs in all countries was associated with higher motivation to create public awareness and conduct community mobilisation. Countries with clear financial management systems that include budgets for CHWs have seen great contributions of CHWs in general healthcare service delivery. Ethiopia and, Bangladesh, among others, successfully implemented a CHW strategy which has seen their vaccination coverage increase.^26^ Costing of CHW programming, both direct and indirect costs, and considering the difference in contexts and the funding sources have been found to be essential for an effective CHW programming.^38^ However, CHWs remuneration varies between countries, because of limited political will, targeted funding, and historical underpinnings and addressing these challenges may improve CHW performance and retention—thus sustainability—of CHW programming.^35 39^ Lack of consistent income could fail to overcome intrahousehold pressures, address competing priorities at the household level, and ultimately lead to decreased morale and retention of CHWs. Standardising CHWs compensation within different settings may be of value, which could be done through governments setting aside a funding stream dedicated to CHWs and considering other sources of funding to supplement existing sources. These strategies may reduce the financial challenges experienced by the CHWs within these countries.

### Trust

Trust and community ownership were generated through the CHWs selection process, which increased their motivation to provide immunisation-related services to the communities in Nepal, Senegal and Zambia. CHWs shared information on vaccination in local languages and reached hard-to-reach areas and minority communities. Being dependable, accountable and respected members of the community, CHWs understand the contexts of the communities they serve.^4 40^ This generates trust and enhances accountability through (1) CHW closeness to the community and local healthcare facilities, (2) uptake of information provided and (3) ease in community entry, and that of the health workers thus reducing child vaccination gaps especially among the illiterate mothers.^41^ Trust between caregivers and CHWs resulted in adequate knowledge and compliance with vaccination recommendations.

Changes in healthcare, including introduction of new vaccines and emerging epidemics, add another layer of work to CHWs and can be addressed through different cadres with specific responsibilities. The recent COVID-19 pandemic highlights the contribution of CHWs in creating demand for vaccination through addressing vaccine hesitancy, leveraging on their existing trust within communities.^42^ Strategic approaches that allow for expanding CHW cadres are necessary to bridge the gap in human resources and provide vaccination services to disadvantaged populations.^43^

Governments have committed to addressing some of the issues that CHWs have voiced; to combat shortages of CHWs in Zambia, the central government committed to increasing the number of CHWs, investing in training, and retaining more workers. The Zambia National Health Strategic Plans and the 2010 and 2019–2021 National CHW strategies instituted guidelines to formalise and harmonise the roles of front-line workers to decrease overlap in responsibilities, as well as uniform incentives. The government committed to bringing 30 000 more workers on board and increasing specialisation of cadres and workers. In 2018, Senegal’s government committed to creating more formalised training for different cadres. The 2019–2021 CHW Strategy identified disharmonised incentives as a challenge and allocated the district health office to coordinate and oversee CHW incentives (such as lunch and transport reimbursement), supplies (like protective clothing), orientation and training, and involvement in national campaigns. In Nepal, FIP worked well to motivate service providers, community workers, volunteers and parents.

### Limitations

This study has several limitations. First, the research tools focused on the factors that drove catalytic change and did not focus on interventions or policies that were unsuccessful. Second, using qualitative methods to understand historical events was challenging; interviewees often spoke about current experiences rather than discussing historical factors. However, research assistants probed respondents to reflect on longitudinal changes in the immunisation programme. Third, the scope of our study was extensive, and CHWs were only one component of our research. Fourth, some policy documents—including national-level strategic plans—were not available for our review. And finally, the COVID-19 pandemic impacted data collection in Senegal. National-level stakeholders shifted focus to COVID-19 response, and FGD participants were unavailable for the discussions as they prioritised their safety over data collection thus slowing the data collection process.

### Conclusion

This study highlighted essential qualities of CHW programmes in countries with high vaccination coverage—namely, organisation, motivation and trust. Each country had distinct CHW cadres involved in the implementation of vaccination programming. Additionally, CHWs were selected from their communities—which generated trust among community members and healthcare staff. CHWs bridge the equity gap in access to vaccination services through involvement of female volunteers, and CHWs enabled wider reach of vaccination services to minority populations and populations in hard-to-reach areas who otherwise could not be easily reached by healthcare workers. Although improvements in vaccination programming were seen in all three countries, CHWs faced challenges in providing adequate services in their communities. Workload, low and inconsistent compensation, inconsistency in training duration and scope, and supervision resulted in demotivation and high turnover of CHWs in all countries. While plans for changes in the CHW programming report increased duties and responsibilities for CHWs, the governments in all countries still need to adequately address issues related to the recognition of CHWs, especially their compensation. Based on their contexts, countries should focus more on ways of addressing such challenges, and country-specific research on strategies to ensure consistent funding for CHWs would be beneficial.

## Supplementary material

10.1136/bmjopen-2023-079358 online supplemental file 1

10.1136/bmjopen-2023-079358 online supplemental file 2

10.1136/bmjopen-2023-079358 online supplemental file 3

## Data Availability

Data are available on reasonable request.

## References

[1] Orenstein WA, Ahmed R (2017). Simply put: vaccination saves lives. Proc Natl Acad Sci USA.

[2] Rodrigues CMC, Plotkin SA (2020). Impact of vaccines; health, economic and social perspectives. Front Microbiol.

[3] WHO (2020). Immunization agenda 2030: A global strategy to leave no one behind.

[4] Pallas SW, Minhas D, Pérez-Escamilla R (2013). Community health workers in Low- and middle-income countries: what do we know about Scaling up and Sustainability. Am J Public Health.

[5] WHO (2016). Global strategy on human resources for health: workforce 2030.

[6] WHO (2021). Health workforce.

[7] Love MB, Gardner K, Legion V (1997). Community health workers: who they are and what they do. *Health Educ Behav*.

[8] Masis L, Gichaga A, Zerayacob T (2021). Community health workers at the dawn of a new era: 4. programme financing. Health Res Policy Syst.

[9] World Health Organization (2018). WHO guideline on health policy and system support to optimize community health worker programmes.

[10] Pérez LM, Martinez J (2008). Community health workers: social justice and policy advocates for community health and well-being. Am J Public Health.

[11] Patel AR, Nowalk MP (2010). Expanding immunization coverage in rural India: A review of evidence for the role of community health workers. Vaccine.

[12] Hester KA, Sakas Z, Ellis AS (2022). Critical success factors for high routine immunization performance: A case study of Nepal. *Vaccine X*.

[13] Hester KA, Sakas Z, Ogutu EA (2023). Critical interventions for demand generation in Zambia, Nepal, and Senegal with regards to the 5c psychological antecedents of vaccination. *Vaccine X*.

[14] WGH (2022). Subsidizing global health: women’s unpaid work in health systems.

[15] Bednarczyk RA, Hester KA, Dixit SM (2021). Protocol: identification and evaluation of critical factors in achieving high and sustained childhood immunization coverage in selected low- and lower-middle income countries. Public and Global Health.

[16] Health EiG (2021). Making better decisions in global health: understand positive Outliers to inform policy and practice. https://www.exemplars.health.

[17] Hester KA, Sakas Z, Ellis AS (2022). Critical success factors for high routine immunization performance: A case study of Senegal. medRxiv. *Vaccine X*.

[18] Micek K, Hester KA, Chanda C (2022). Critical success factors for routine immunization performance: A case study of Zambia 2000 to 2018. *Vaccine X*.

[19] Pfadenhauer LM, Gerhardus A, Mozygemba K (2017). Making sense of complexity in context and implementation: the context and implementation of complex interventions (CICI) framework. Implement Sci.

[20] Damschroder LJ, Aron DC, Keith RE (2009). Fostering implementation of health services research findings into practice: a consolidated framework for advancing implementation science. Implementation Sci.

[21] Hennink M, Kaiser BN (2022). Sample sizes for saturation in qualitative research: A systematic review of empirical tests. Social Science & Medicine.

[22] Hester KA (2021). Exemplars in vaccine delivery OSF home. https://osf.io/7ys4a/?view_only=739ca7a72f9749118b4aa3d2f7b655d9.

[23] Katigbak C, Van Devanter N, Islam N (2015). Partners in health: a conceptual framework for the role of community health workers in facilitating patients' adoption of healthy behaviors. Am J Public Health.

[24] Sripad P, McClair TL, Casseus A (2021). Measuring client trust in community health workers: A multi-country validation study. J Glob Health.

[25] Agarwal S, Sripad P, Johnson C (2019). A conceptual framework for measuring community health workforce performance within primary health care systems. Hum Resour Health.

[26] Perry H (2013). A brief history of community health worker programs. developing and strengthening community health worker programs at scale: a reference guide and case studies for program managers and policymakers, USAID, MCHIP.

[27] Phiri SC, Prust ML, Chibawe CP (2017). An exploration of Facilitators and challenges in the scale-up of a national, public sector community health worker cadre in Zambia: a qualitative study. Hum Resour Health.

[28] COUNCIL OE (2008). Towards an active, fair and socially cohesive Europe report of high level task force on social cohesion. TFSC 2007.

[29] Olaniran A, Madaj B, Bar-Zeev S (2022). Factors influencing motivation and job satisfaction of community health workers in Africa and Asia-A multi-country study. Int J Health Plann Manage.

[30] Sialubanje C, Massar K, Horstkotte L (2017). Increasing utilisation of skilled facility-based maternal Healthcare services in rural Zambia: the role of safe motherhood action groups. Reprod Health.

[31] Kasteng F, Settumba S, Källander K (2016). Valuing the work of unpaid community health workers and exploring the incentives to volunteering in rural Africa. Health Policy Plan.

[32] Panjabi R (2019). Community health workers are vital; governments should be paying them.

[33] Coffey C, Espinoza Revollo P, Harvey R (2020). Time to care: unpaid and underpaid care work and the global inequality crisis: Oxfam.

[34] Maes K (2016). The Lives of Community Helth Workers: Local Labor and Global Health in Urban Ethiopia.

[35] Colvin CJ, Hodgins S, Perry HB (2021). Community health workers at the dawn of a new era: 8. incentives and remuneration. Health Res Policy Syst.

[36] Raven J, Wurie H, Idriss A (2020). How should community health workers in fragile contexts be supported: qualitative evidence from Sierra Leone. *Hum Resour Health*.

[37] Central C (2018). Gender and community health workers: three focus areas for programme managers and policy makers. https://chwcentral.org/twg_article/gender-and-community-health-workers-three-focus-areas-for-programme-managers-and-policy-makers.

[38] Crigler HPaL (2014). Developing and strengthening community health worker program at scale.

[39] Ballard M, Westgate C, Alban R (2021). Compensation models for community health workers: comparison of legal frameworks across five countries. J Glob Health.

[40] Kane S, Kok M, Ormel H (2016). Limits and opportunities to community health worker empowerment: A multi-country comparative study. Soc Sci Med.

[41] Lee H-Y, Oh J, Heo J (2019). Association between maternal literacy and child vaccination in Ethiopia and southeastern India and the moderating role of health workers: a Multilevel regression analysis of the young lives study. Glob Health Action.

[42] Dixit SM, Sarr M, Gueye DM (2021). Addressing disruptions in childhood routine Immunisation services during the COVID-19 pandemic: perspectives from Nepal, Senegal and Liberia. BMJ Glob Health.

[43] Schleiff MJ, Aitken I, Alam MA (2021). Community health workers at the dawn of a new era: 6. recruitment, training, and continuing education. Health Res Policy Syst.

[44] Pallas SW, Minhas D, Pérez-Escamilla R (2013). Community health workers in low-and middle-income countries: what do we know about Scaling up and Sustainability. Am J Public Health.

[45] Gottert A, McClair TL, Hossain S (2021). Development and validation of a multi-dimensional scale to assess community health worker motivation. J Glob Health.

[46] Franco LM, Bennett S, Kanfer R (2002). Health sector reform and public sector health worker motivation: a conceptual framework. Soc Sci Med.

[47] O’Mara-Eves A, Brunton G, Oliver S (2015). The effectiveness of community engagement in public health interventions for disadvantaged groups: a meta-analysis. BMC Public Health.

[48] Rohman A, Eliyana A, Purwana D (2020). Individual and organizational factors’ effect on knowledge sharing behavior. JESI.

[49] Sakas Z, Hester KA, Ellis AS Critical success factors for high routine immunization performance: A multiple case study analysis of nepal, senegal, and zambia. Public and Global Health.

[50] Khatri RB, Mishra SR, Khanal V (2017). Female community health volunteers in community-based health programs of Nepal: future perspective. Front Public Health.

[51] Kristen Devlin KFE, Pandit-Rajani T (2016). Community Health Systems Catalog Country Profile: Senegal.

[52] Cothran D (2019). Senegal’s community-based health system model: structure, strategies, and learning.

[53] Ensor T, Green C, Quigley P (2014). Mobilizing communities to improve maternal health: results of an intervention in rural Zambia. Bull World Health Organ.

[54] Jacobs C, Michelo C, Moshabela M (2018). Implementation of a community-based intervention in the most rural and remote districts of Zambia: a process evaluation of safe motherhood action groups. Implement Sci.

[55] Zulu C, Michelo C, Mubita-Ngoma C (2017). Evaluation of training and implementation program for community-based child growth monitors and promoters in Zambia.

