# Supplementary material for: Success in vaccination programming through community health workers: a qualitative analysis of interviews and focus group discussions from Nepal, Senegal and Zambia
Source: BMJ Open. 2024 Apr 3;14(4):e079358. doi: 10.1136/bmjopen-2023-079358 (PMC11146414; doi:10.1136/bmjopen-2023-079358)
Supplement: online supplemental file 1 [file bmjopen-2023-079358supp001.pdf]

VACCINE EXEMPLARS
Key Informant Interview Guide – Tool 1b ALL COUNTRIES

Objectives:

- To understand the vaccine intervention characteristics during country-specific change points in DTP1/DTP3 coverage
- To identify key internal and external actors, stakeholders, and partnerships in the immunization program both now and in the past
- To identify the strategies behind current immunization programs to inform current context
- To identify previous immunization activities that were key during change points in the DTP1/DTP3 curve
- To understand key barriers and facilitators of interventions in the immunization sector

<table>
<tr><td colspan="2">PART I – DEMOGRAPHIC AND BASIC INFORMATION
Can be asked by interviewer or filled out by note taker.</td></tr>
<tr><td colspan="2">A01. Researcher/Interviewer name:</td></tr>
<tr><td colspan="2">A01b. Note taker(s) name(s):</td></tr>
<tr><td colspan="2">A01c. Translator name:</td></tr>
<tr><td colspan="2">A01d. Copy belongs to:</td></tr>
<tr><td colspan="2">A02. Consent given: Y / N
Time of consent ____ : ____ am/pm</td></tr>
<tr><td>A03. Date (dd/mm/yy): _____ /_____ / _____</td><td>A04a. Interview start time ____ : ____ am/pm
A04b. Interview end time ____ : ____ am/pm
A05. Recorder(s) used:</td></tr>
<tr><td>A06. Name of Respondent interviewed:</td><td>A07. Designation/title of Respondent interviewed:</td></tr>
<tr><td>A08. Organization name:</td><td>A09. Years in Organization:</td></tr>
</table>

PART II – INTERVIEW INTRODUCTION

<u>Interviewer Script</u>: Thank you very much for your willingness to meet with us today and share your experience and knowledge regarding the immunization program in ______ (COUNTRY). I'd like to begin by learning about you and your role related to the immunization program.

1. Can you tell me about your role in ______ (ORGANIZATION) ?

~ Individual Setting ~

Follow up:

- *How does your role relate to the immunization program?*
- *Responsibilities (past and present)?*
- *How long have you been working on vaccines/with the immunization program(s)?*
- *What drew your interest to vaccines/immunization programs? What type of projects have you worked on?*

PART IIa – IMMUNIZATION PROGRAM

Read the following script aloud:

<u>Interviewer Script</u>: Now I'd like to learn about the immunization program, and about the increase in coverage since the year 2000. I'd also like to know about what relevant events that may have contributed to this change, and how the immunization program is funded.

2. Will you tell me a bit about the current state of the vaccination program in ______ (COUNTRY). ?

~ Outer Setting ~

Follow up:

- *Who are the main decision makers at this point in time?*

3. Who are the main government actors involved in the current immunization program?

~ Outer Setting ~
~ Engaging; Process ~
~ RQ2. Political Will ~
~ RQ5. Knowledge and Resource Sharing ~
~RQ6. Workforce ~

Follow up:

- *If there were multiple governmental agencies, how were they related and what responsibility does each take in regard to vaccination efforts?*
- *What other activities did governmental agencies engaged in vaccination undertake?*
- *Which organizations or ministries are responsible for how the immunization system performs from top to bottom, inclusive of the regional level?*

4. How does the chain of command/structure within the immunization sector work?

Follow up:

- *What kind of directives come from the ministry?*
- *How do you report changes that are made in programming?*
- *Who do you report this to?*
- *What kind of changes in programming do people at different levels (national, provincial, district, health facility) contribute to?*
- *How are you made aware of changes in personnel within the immunization program?*
- *Who within the program reports to you?*
- *Who do you report to?*

5. Who are the main partner organizations involved in the current immunization program?
(NOTE: DON'T EXPECT TOO MUCH FOLLOW UP FOR G, H, I)

~ Outer Setting ~
~ Engaging; Process ~
~ RQ5. Knowledge and Resource Sharing ~
~RQ6. Workforce ~

Follow up:

- *What are their roles?*
- *What are the local donor, individuals, and local NGOs that contribute to the sector?*
- *Who makes up the internal and external-workgroups? Can you define these actors - Ministries, INGOs, local NGOs, private sector?*
- *How do the organizations/stakeholders (individual actors) coordinate with each other?*

6. When do you think the vaccination program started to show improvements?

~ Outer Setting ~
~ Inner Setting~

Follow up:

- *What happened in* $\overline{\quad YEAR(S) \quad}$ *to affect vaccine coverage?*
- *Why do you think this is when the program improved? What are the events?*
- *How would you describe the environment surrounding the vaccine program during this period? How did this make the change possible?*
- *What do you think catalyzed this change? (If examples needed, use beneficiary needs, policy, legislation, individual champions, politicians?*
- *Who were the main decision makers at this point in time? What were their incentives for performing well? Which party was in power at this time?*
- *Do other individuals agree with this assessment, or is there something else that is believed to be behind change? For example, do politicians think this is also the driver behind the increase in coverage?*
- *Which, of the reasons you gave me, do you consider the most important in improving immunization coverage? Why?*

7. ***(NOTE: ONLY SHOW/THIS to CONNECT TO PREVIOUS ANSWER)*** This graph shows the DTP1/DTP3 coverage trend since 2000 in $\overline{\quad COUNTRY \quad}$. In addition to what you've already said, what else can you tell us about these time points where coverage increased? What important or relevant events do you believe contributed to the increases?

8. Who were the key stakeholders during the time of improvement?

~ Outer Setting ~
~ RQ2. Political Will ~
~ RQ5. Knowledge and Resource Sharing ~
~ RQ6. Workforce ~

Follow up:

- *Ministries, partner organizations?*
- *What role did they play?*
- *If there were multiple governmental agencies, how were they related and what responsibility does each take in regard to vaccination efforts?Who made up the internal and external-workgroups?*
    - *Can you define these actors* **-** *Ministries, INGOs, local NGOs, private sector?*
- *What other activities did governmental agencies engaged in vaccination undertake?*
- *What has the turnover in these positions been like?*
- *Have the key people identified been in the same position for extended periods? What was the mandated term?*
    - *What are their motivations in involvement?*
    - *Can you connect me to XXX?*

-

9. What do you consider to be the main barriers to the improvement in immunization coverage in $\underset{YEAR(S)}{\_\_\_\_\_\_\_\_\_\_}$ before the increase?

~ Outer Setting ~

Follow up:

- *How was each barrier addressed?* ***(NOTE: REFER BACK TO SPECIFIC BARRIERS MENTIONED BY PARTICIPANT)***
- *What was the motivation behind addressing the barriers?*

10. What were the main sources of funding for immunization programming during this time period? ***(NOTE: FOR TOOLS H and I, JUST ASK THE MAIN QUESTION. DON'T EXPECT TO PROBE)***

~ Available Resources; Inner Setting ~
~ RQ2. Political Will ~

Follow up:

Ask these questions at each level -

- *Is there a fund specifically for vaccines, or is there just a general health fund?*
  - *How are these funds spent?*
- *How much of the overall health budget is allocated to vaccination activities?*
- *Is there an overall vaccine budget, or is it separated by vaccine?*
- *Are there financial barriers to increasing the vaccination rates? If so, what?*
  - *Which specific barrier(s) require more funding?*
- *Which level of government is responsible for spending the resources**? (EXAMPLES: Province, district, health facility)***
  - *How much power do you have to spend the money the way that you want to?*
- *Is there an advisory board/audit that checks how money is spent? Does the advisory board check the expenses for each level; regional, district, health center?*
- *Are there any restrictions on how contracts are assigned? **(EXAMPLES: Grants, distributors for supplies or vaccine, etc.)***
- *Can you share supporting documentation with me? If not, where can I access this, and do you know who may be able to assist me?*

PART III – INTERVIEWER TO ASK SECTIONS BELOW AS DETERMINED BY TITLE/DEPARTMENT OF RESPONDENT:

Central -

A. Minister of Health, Minister Representative, or General Secretary …………………...….…. 7
B. Ministry Departments of Epidemiology, Surveillance, EPI, Vaccines, Family Health….….. 10
C. Ministry Departments (or liaisons) of Planning, Statistics, Research, Human Resources, Communication and Development ……………………………………………………….… .20
D. Ministry Departments (or liaisons) of Infrastructure, Equipment, Finance ……………...…. 24
E. Partner Organizations, such as WHO, UNICEF, CDC, USAID etc.; NITAG, Professional Organization members …………………………………………………...………….. 26

Subnational -

G. Region Head, Surveillance, Prevention, and Vaccination Personnel ……………….….…. 30
H. District Head, Surveillance, Prevention, Vaccination Personnel ……………………....….38
I. Clinic Head …….…………………………………………………………………….….46
J. Community Leader…………………………………………………………………………53

PART IV – CONCLUSION ………………………………………………………………………57

SECTION A. MINISTER OF HEALTH, MINISTER REPRESENTATIVE, OR GENERAL SECRETARY

Objectives:

- To understand the vaccine intervention characteristics during country-specific change points in DTP1/DTP3 coverage
- To identify key internal and external actors, stakeholders, and partnerships in the immunization program both now and in the past
- To explore decision-making process in selecting the strategies and activities in the immunization program that created change points in DTP1/DTP3 coverage
- To identify the strategies behind current immunization programs to inform current context
- To identify previous immunization activities that were key during change points in the DTP1/DTP3 curve
- To understand key barriers and facilitators of interventions in the immunization sector

PART A1 – POLICY

1. What is the current state of legislation regarding vaccines and immunizations (laws, constitutional requirements)?

~ External Policies and Incentives; Outer Setting ~
~ RQ2. Political Will ~

Follow up:

- *Where would I find copies/documentation of that legislation?*
- ***If there is no knowledge of current legislation:*** *Is there past legislation that is being followed?*

2. How was legislation on vaccines and immunization developed?

~ External Policies and Incentives; Outer Setting ~
~ RQ2. Political Will ~

NOTE: BASE QUESTION ON THE PREVIOUS ANSWER.

Follow up:

- *When was key legislation passed?*
- *Who played a role in this (organization or person)?*

3. What national policies do you think were important in increasing vaccine coverage?

~ External Policies and Incentives; Outer Setting ~
~ RQ2. Political Will ~
~ RQ7. Vaccine Policy and Policy Enforcement ~

Follow up:

- *Which do you think has been the most important piece of legislation in contributing to the current rates of immunization coverage?*
- *What rule/regulation did you find to be most important/effective in increasing vaccine coverage?*
- *What role does the Ministry of Finance have in determining these rules and regulations?*
- *What role does the Ministry of Finance have in implementing the rules and regulations?*
- *Which supporting documents could you share with me?*

PART A2 – STAKEHOLDERS

4. How have financial investment decisions regarding the immunization sector been made?

~ Outer Setting ~
~ RQ1b. Immunization System Context Within the Health Sector ~

Follow up:

- *How is this relative to other areas of the healthcare system/sector?*
- *Who determines this?*
- *What percentage/amount of funding is spent on vaccinations as opposed to other health sector areas, including disease surveillance, coverage measurement, accountability?*
- ***(NOTE: Some probes may have been addressed in Part 2. Make sure to follow up on relevant information but don't repeat questions)***

PART A3 – IMMUNIZATION PROGRAM

5. What were key activities in planning changes for the immunization program? ***(NOTE: Refer to answers in section 2).***

~ Planning; Process ~
~ RQ5. Knowledge and Resource Sharing ~
~ RQ6. Workforce ~

Follow up:

- *Meeting, working group*
- *Did you have an action plan?*
- *A theory of change or log frame?*

6. What challenges did you face in the early planning phases?

~ Planning; Process ~

Follow up:

- *How were these challenges overcome* ***(EX: Internal to agency/external to agency)****?*

7. What kind of evidence or information **(EX: outbreaks, surveillance data)** informed changes in programming or interventions?

~ Planning; Process ~
~ RQ5. Knowledge and Resource Sharing ~

Follow up:

- *You mentioned ______________ (DATA SOURCE NAME(S)). What role did this information have in the push to increase vaccine coverage?*
- *Can you explain how ______________ (DATA SOURCE NAME(S)) is collected?*
- *How is this ______________ (DATA SOURCE NAME(S)) integrated into other health systems?*
- *[If surveillance system not mentioned] Does ______________ (COUNTRY) have a surveillance system?*

~ Planning; Process~
~ RQ5. Knowledge and Resource Sharing~

8. What areas of the country have seen the greatest improvement in vaccine rates?

~ Reflecting and Evaluating; Process ~
~ RQ3. Information Systems ~

~ RQ4. Intent/Demand for Vaccines ~

Follow up:

- *Why do you think these areas had the greatest improvement?*
    - *Was a project/intervention specifically tailored to this location?*
    - *Was it targeted as a critical area for change?*
- *What areas saw the quickest improvement?*
    - *Was a project/intervention specifically tailored to this location?*
    - *Was it targeted as a critical area for change?*

9. What areas of the country did not see significant changes?

~ Reflecting and Evaluating; Process ~
~ RQ3. Information Systems ~
~ RQ4. Intent/Demand for Vaccines ~

Follow up:

- *Why do you think this is?* (**NOTE: May have already had high coverage, may not relate to challenges***)*
- *What were the challenges?*
    - *Why?*
    - *Was a project/intervention specifically tailored to this location?*
        - *Implementation failure*
        - *Theory failure*
    - *Was it targeted as a critical area for change?*
- *What has been/might be done to mitigate this?*

10. What are the greatest barriers to sustained performance?

~ Reflecting and Evaluating; Process~

11. What are the perceptions of corruption in the public sector?

Follow up:

- *Are you aware of any anti-corruption measures that have been put in places?*
- *What kind of accountability measures are in place that would prevent corruption?*
- *Perceptions of quality of electoral processes?*
- *Judicial institutions?*
- *Anti-corruption efforts?*

SECTION B. MINISTRY DEPARTMENTS OF EPIDEMIOLOGY, SURVEILLANCE, EPI, VACCINES, FAMILY HEALTH, GAVI REPRESENTATIVE

Objectives:

- To understand the vaccine intervention characteristics during country-specific change points in DTP1/DTP3 coverage
- To identify key internal and external actors, stakeholders, and partnerships in the immunization program both now and in the past
- To explore decision-making process in selecting the strategies and activities in the immunization program that created change points in DTP1/DTP3 coverage
- To identify the strategies behind current immunization programs to inform current context
- To identify previous immunization activities that were key during change points in the DTP1/DTP3 curve
- To understand key barriers and facilitators of interventions in the immunization sector

PART B1 – POLICY

1. What is the current state of legislation regarding vaccines and immunization?

~ External Policies; Outer Setting ~
~ RQ2. Political Will ~

NOTE: EXAMPLES INCLUDE LAWS, CONSTITUTIONAL REQUIRMENTS

Follow up:

- *Where would I find copies/documentation of that legislation?*

2. How was legislation on vaccines and immunization developed? What year was it started?

~ External Policies; Outer Setting ~
~ RQ2. Political Will ~

Follow up:

- *When was key legislation passed?*
- *Which do you think has been the most important piece of legislation in contributing to the current rates of DTP3 coverage?*
- *Where would I find copies/documentation of that legislation?*

3. What policies were made at the national level that you think were important in increasing vaccine coverage during $\underset{YEAR(S)}{\underline{\hspace{3cm}}}$?

~ External Policies; Outer Setting ~
~ RQ2. Political Will ~
~ RQ7. Vaccine Policy and Policy Enforcement ~

Follow up:

- *How were these policies informed?*
- *What was the reason/rationale for creating these new policies?*
- *What is the policy making process, and how are new policies or recommendations disseminated and implemented once approved?*
- *How do they work in both vertical (e.g. across ministries) and horizontal (e.g. within different levels of a particular ministry)?*
- *What led to the selection of these policies?*
- *What challenges did you encounter with any new policies made?*

4. Are there relevant polices at the subnational/regional level? If so, what?

~ External Policies; Outer Setting ~
~ RQ2. Political Will ~
~ RQ7. Vaccine Policy and Policy Enforcement ~

Follow up:

- *If there are no policies at the subnational/regional level, why?*
  - *Do all regions need to follow the national law, or can they create their own?*
- *How were these policies informed?*
- *What led to the selection of these policies?*
- *What data were most important in setting policy?*
- *What kind of global/regional resources were utilized?*
- *How were policies implemented?*
-

PART B2 – STAKEHOLDERS

5. How have decisions on types of financial investments in the immunization sector been made, relative to other areas of the healthcare system/sector?

~ Outer Setting ~
~ RQ1b. Immunization System Context Within the Health Sector ~

Follow up:

- *Who determines this?*
- *What percentage/amount of funding is spent on vaccinations as opposed to other health sector areas, including disease surveillance, coverage measurement, accountability?*

PART B3 – IMMUNIZATION PROGRAM

6. What sort of intervention(s) do you attribute to the increase in immunization coverage?

~ Planning; Process ~
~ RQ4. Intent/Demand for Vaccines ~
~ RQ5. Knowledge and Resource Sharing ~
~ RQ6. Workforce ~

Follow up:

- *Prior to the intervention, what kind of piloting or testing of the intervention occurred?*
- *Did the service delivery of vaccinations change after the intervention? How?*
- *Where did this take place nationally?*

- *What was learned in this process?*

7. What were key activities in planning changes for the program?

~ Planning; Process ~
~ RQ5. Knowledge and Resource Sharing ~
~ RQ6. Workforce ~

Follow up:

- *What was your role?*
-
- *Did you have an action plan?*
- *A theory of change or log frame?*

8. What challenges did you face in the early planning phases?

~ Planning; Process ~

Follow up:

- *How were these challenges overcome* ***(EX: Internal to agency/external to agency)****?*

9. What were key training activities of [intervention/activities mentioned in Q6]?

~ Planning & Executing; Process ~
~ RQ5. Knowledge and Resource Sharing ~
~ RQ6. Workforce ~

Follow up:
Do you have the following information, and can we access it?

- *Who was involved in trainings* ***(NOTE: at all levels)****?*
- *Who taught the training?*
- *How were they selected*
  - *Trainers*
  - *Trainees?*
- *What was their motivation/incentive for involvement?*
- *How were the trainings conducted?*
- *Were individuals participating in the training given opportunities to provide feedback?*
  - *If so, how?*
  - *If not, why?*
- *If so, did this change the implementation of the program in the region*?

10. If no training activities, what modality did the intervention have?

Follow up:

- *What modalities were used for different aspects of the project, generally?*

(NOTE: FOLLOW UP IF THE INTERVENTION SEEMS UNCLEAR, E.G. IS MORE FUNDING AVAILABLE? LOW TURNOVER OF STAFF? ETC.)

11. What kind of evidence/information (***EX: outbreaks, surveillance data***) did you use to inform changes/interventions?

~ Planning; Process ~
~ RQ5. Knowledge and Resource Sharing ~

Follow up:

- *You mentioned* ___________ *(DATA SOURCE NAME(S)). What role did this information have in the push to increase vaccine coverage?*
- *Can you explain how* ___________ *(DATA SOURCE NAME(S)) is collected?*
- *How is this* ___________ *(DATA SOURCE NAME(S)) integrated into other health systems?*
- *[IF SURVEILLANCE SYSTEM NOT MENTIONED] How does the surveillance system in* ___________ *(COUNTRY) work?*

~ Planning; Process ~

~ RQ5. Knowledge and Resource Sharing~

12. What measures did you find critical for success when implementing interventions?

~ Reflecting and Evaluating; Process ~

~ Q6. Workforce ~

Follow up:

- *What advice would you give to another person/region/country who was going to implement a similar program/project to yours?*

13. What challenges/difficulties did you encounter when implementing interventions?

~ Reflecting and Evaluating; Process ~

Follow up:

- *How were these challenges overcome?*

14. What were the greatest challenges in project implementation?

~ Reflecting and Evaluating; Process ~

Follow up:

- *Of those challenges you mentioned, which was the greatest?*
- *Was the project specifically tailored to this location?*
- *Was it targeted as a critical area for change?*
- *How were decisions made about what to do?*
- *Who contributed to this decision-making? Why?*

15. What areas of the country have seen the greatest improvement in vaccine coverage that you would attribute to this intervention?

~ Evaluation; Process~

~ RQ3. Information Systems ~

~ RQ4. Intent/Demand for Vaccines ~

Follow up:

- *Was the project specifically tailored to this location?*
- *Was it targeted as a critical area for change?*
- *What areas saw the quickest improvement?*
- *Why do you think these areas had the greatest improvement?*

16. During the improvement period, did you recommend any new vaccine introductions ***(EX: PCV, rotavirus)***?

~ Planning; Process ~

~ RQ4. Intent/Demand for Vaccines ~<br>*~ RQ5. Knowledge and Resource Sharing ~*<br>*~ RQ6. Workforce ~*
Follow up:<br><br>• *How did they affect the overall delivery system?*
•
17. What areas did not experience significant change in coverage after implementation of the intervention?<br><br>***~ Evaluation; Process~***<br>*~ RQ3. Information Systems ~*<br>*~ RQ4. Intent/Demand for Vaccines ~*
Follow up:<br><br>• *Why do you think this is? (**EX: May have already had high coverage, may not relate to challenges)***<br>• *What were the challenges? Why?*<br>• *What has been/might be done to mitigate this?*
18. What populations are currently the most vulnerable to low coverage?<br><br>***~ Evaluation; Process~***
19. What populations historically were the most vulnerable to low coverage?<br><br>***~ Evaluation; Process~***
Follow up:<br><br>• *How did you identify these populations as vulnerable?*<br>○ *Both previously and now*<br>• *How did you reach out to these populations?*<br>• *What strategy/strategies do you use to reduce inequities?*<br>• *What strategy did you previously use to reduce inequities?*<br>• *What kind of data supports this?*<br>• *Is this data you can share with me?*

•
20. What interventions were offered through community health workers (CHWs)?<br><br>***~ Executing; Process ~***<br>*~ RQ6. Workforce ~*
Follow up:<br><br>• *How were/are CHWs motivated/incentivized for involvement?*<br>○ *Financial*<br>○ *Respect*<br>○ *Opportunities for learning/capacity building*<br>○ *Recruited by trusted person*<br>▪ *Unable to say*<br>• *What kind of trainings are offered to:*<br>○ *Increase learning?*<br>○ *Build capacity*<br>• *What are the qualifications to be a CHW?*<br>• *Are their responsibilities increased over time?*<br>• *How do they prioritize responsibilities?*<br>• *How do CHWs become better at what they do over time?*
21. What interventions were offered at various levels of the health system? To providers?<br><br>***~ Execution; Process ~***<br>*~ RQ3. Information Systems ~*<br>*~ RQ6. Workforce ~*
Follow up:<br><br>• *Province/district/health post*<br>• *How were these developed?*<br>• *How were they implemented?*<br>○ *Training?*<br>• *Why were they implemented in the way that they were?*<br>• *Who oversaw these interventions?*<br>• *What led to the selection of these interventions?*<br>• *Was there variation in different geographic regions etc. If so, why?*
•
•
22. What programs were offered to parents or first-time parents?<br>***~ Intervention Characteristics~***<br>***~Outer Setting ~***<br>*~ RQ4. Intent/Demand for Vaccines ~*
Follow up:<br><br>• *Were these tailored to different areas/cultures/religions/languages/etc.?*

- *How were these targeted specifically to mothers, fathers or alternative caretakers?*
- *How were they developed?*
- *Where did you get the idea for these interventions?*
  - *Did you use a framework or intervention to inform your intervention?*
- *How were they delivered?*
- *Who was engaged in delivery?*
- *What determined their involvement?*
- *What kinds of modalities were used?*
- *What kind of communication materials (if any) were utilized?*
  - *Mass media?*
  - *Dissemination through health centers?*
  - *Other?*
- *How did you get individuals to participate in this program?*
- *What led to their selection?*
- *Could you share any materials with us?*

**IF PARTICIPANT IS UNABLE TO ANSWER, NOT NECESSARY TO GO THROUGH EACH PROBE.*

~ Beneficiary needs and resources ~

23. Were the programs used in combination with other health interventions?

~ Outer Setting ~
~ RQ4. Intent/Demand for Vaccines ~

Follow up:

- *Were there any communication materials, strategies, or campaigns in conjunction with the other healthcare interventions?*
- *Or communications (IEC materials, mass media, etc.)?*
- *How was this coordinated?*
- *Who took ultimate responsibility for outcomes?*

24. Did you make any changes to the supply chain in ______________________________
YEAR(S) or during specific interventions mentioned

~ Execution; Process ~
~ Q6. Workforce ~

Follow up:

- *How were these changes made?*
- *Why was this change made?*
  - *Has this change had the anticipated impact (on coverage or otherwise)?*

25. Were there any changes made to the data systems in ___________________________ ?
YEAR(S)or specific intervetions mentioned

~ Execution; Process ~
~ Q3. Information Systems ~
~ Q6. Workforce ~

Follow up:

- *How were these changes made?*
- *Why was this change made?*
    - *Has this change had the anticipated impact (on coverage or otherwise)?*
- *Did you develop or already have in place a clinic-based immunization record keeping system to track immunizations administered to individuals?*
- *Who kept these records? Did parents hold records?*
- *What are parents' involvement in record keeping for their child's vaccinations?*
- *To what extent was technology a component of record keeping (computer, phone data collection)?*

26. During those years, did you have any kind of reminder/recall system for children's vaccinations?

~ Execution; Process~
~ Q3. Information Systems ~
~ Q6. Workforce ~

NOTE: REMINDER SYSTEM DEFINED AS A MESSAGE SENT BEFORE APPOINTMENTS TO REMIND PARENTS TO COME; RECALL SYSTEM DEFINED AS A NOTICE SENT TO PARENTS WHO FAIL TO KEEP THEIR APPOINTMENTS.

Follow up:

- *If so, how was this implemented?*
- *What kind of system was this?*
    - *Cell phone text reminders?*
    - *Community health worker in-person reminders?*
    - *Church/religious institution reminders?*
- *Was technology a component of this system?*
- *Who carries out the reminders or recall?*
- *Were there interventions offered through the CHWs?*

•
•
27. How do you monitor the performance of immunization programs?<br><br>***~ Evaluation; Process~***<br>*~ RQ3. Information Systems ~*
28. How does this differ from how immunization programs were monitored in the past?<br><br>***~ Evaluation; Process ~***<br>*~ RQ3. Information Systems ~*
Follow up:<br><br>• *Who is responsible for collecting data related to measures?*<br>• *How is data used?*<br>• *What measures are included?*<br>• *How often is data collected/shared?*<br>• *How is this information communicated on a local/regional/national level?*<br>• *How do you perceive the quality of data collected?*<br>• *If data quality is poor, is it/how is it used? If poor, what would improve the quality?*<br>• *Are there "independent" checks of the reported data to ensure they are valid?*<br>• *Would any other types of data have been helpful in managing the program?*<br>• *Is there an independent group that evaluates the entire program periodically? If so, please give some details regarding the composition of the group, how it is chosen, and what activities they undertake to evaluate the program?*
29. Is there any recognition for being at the top levels of coverage in the country or for making major improvements? If so, can you give some examples of recognition/incentives?
Follow up:<br><br>• *If yes, have you noticed any issues of misreporting due to the possibility of recognition/incentives?*
30. How do you measure the impact of immunization programs?<br><br>***~ Evaluation; Process ~***<br>*~ RQ3. Information Systems ~*

31. Is there public opinion data on vaccination?

~ Evaluation; Process ~
~ RQ3. Information Systems ~

Follow up:

- *If yes, how can we access this data?*

32. What factors have maintained the sustainability of the immunization program?

~ Evaluation; Process ~

a. **Partnerships:** Does the program have diverse organizations invested in its success?
b. **Partnerships**: What does the collaboration between these diverse organizations look like?
c. **Organizational capacity**: Were there organizational systems in place to support the program needs?
d. **Champion**: Does the program have strong champions? ***(EX: Political or advocacy support outside of the program?)***
e. **Funding**: Does the program have sustained funding?
   i. *Follow up: How important was support of the legislature to program success **(EX. appropriating additional funding not initially requesting by the executive branch**)?*
f. **Evaluation**: How do evaluation results inform future program planning, implementation, and funding?
   i. *Follow up: What kind of data drives this?*
   ii. *Can you share any data with us?*

g. **Adaptation**: How does the immunization program adapt to new evidence?

~ Evaluation; Process~
~RQ1. Why and How? ~
~ RQ 3. Information Systems ~

Follow up:

- *Where do you learn about new evidence?*

h. **Adaptation**: How does the immunization program adapt to new information?

i. **Communication**: How does the immunization program secure community support or awareness of the issue of childhood vaccines?

~ Evaluation; Process~
~ RQ3. Information Systems ~
~ RQ4. Intent/Demand for Vaccines ~
~ RQ5. Knowledge and Resource Sharing ~
~ RQ6. Workforce ~

Follow up:

- *How does the immunization program maintain community support of childhood vaccines?*
- *How does the immunization program maintain awareness of childhood vaccines?*

• *To what extent is the immunization program resilient from rumors and misinformation regarding immunizations?*<br>• *How could resilience be improved?*
j. **Media**: How is vaccination portrayed and discussed by the media?<br><br>***~ Outer Setting ~***<br>*~ RQ3. Information Systems ~*<br>*~ RQ4. Intent/Demand for Vaccines ~*<br>*~ RQ5. Knowledge and Resource Sharing ~*
Follow up:<br><br>• *What role did the media play in improved coverage?*<br>• *How did the media become involved and how was that involvement sustained?*
33. What kind of planning is there for future resource needs?<br><br>***~ Evaluation; Process~***<br>*~ RQ5. Knowledge and Resource Sharing ~*
34. What are the greatest barriers to sustained coverage?<br><br>***~ Reflection; Process~***
35. What are the perceptions of corruption in the public sector?<br><br>***~ Inner Setting ~***<br>*~ RQ2. Political Will ~*
Follow up:<br>• *Perceptions of quality of electoral processes?*<br>• *Judicial institutions?*<br>• *Anti-corruption efforts?*

SECTION C. MINISTRY DEPARTMENTS (OR LIAISONS) OF PLANNING, STATISTICS, RESEARCH, HUMAN RESOURCES, COMMUNICATION AND DEVELOPMENT

Objectives:

- To understand the vaccine intervention characteristics during country-specific change points in DTP1/DTP3 coverage
- To identify key internal and external actors, stakeholders, and partnerships in the immunization program both now and in the past
- To explore decision-making process in selecting the strategies and activities in the immunization program that created change points in DTP1/DTP3 coverage
- To identify the strategies behind current immunization programs to inform current context
- To identify previous immunization activities that were key during change points in the DTP1/DTP3 curve
- To understand key barriers and facilitators of interventions in the immunization sector

PART C1 – IMMUNIZATION PROGRAM

1. What were key activities in planning changes for the immunization program?

~ Planning; Process ~
~ RQ5. Knowledge and Resource Sharing ~
~ RQ6. Workforce ~

NOTE: REFER TO ANSWERS GIVEN IN SECTION 2.
IF NOT INVOLVED, SKIP TO Q4.

Follow up:

- *Did you have an action plan?*
- *A theory of change or log frame?*

2. What challenges did you face in the early planning phases?

~ Planning; Process ~

Follow up:

- *How were these challenges overcome **(EX. Internal to agency/external to agency)**?*

3. What kind of evidence/information **(EX: outbreaks, surveillance data)** did you use to determine changes/interventions?

~ Planning; Process~
~ RQ5. Knowledge and Resource Sharing ~

Follow up:

- *You mentioned* ______________ (DATA SOURCE NAME(S)). *What role did this information have in the push to increase vaccine coverage?*
- *Can you explain how* ______________ (DATA SOURCE NAME(S)) *is collected?*
- *How is this* ______________ (DATA SOURCE NAME(S)) *integrated into other systems?*
- *[If surveillance system not mentioned] How does the surveillance system in* ______________ (COUNTRY) *work?*

4. Prior to the critical changes/intervention, what kind of piloting/testing of the intervention occurred?

~ Planning; Process~

~ RQ4. Intent/Demand for Vaccines ~<br>*~ RQ5. Knowledge and Resource Sharing ~*<br>*~ RQ6. Workforce ~*
Follow up:<br>• *Did the service delivery of vaccinations change?*<br>• *Where did this take place?*<br>• *What was learned in this process?*
5. What were key training or technical assistance activities of the intervention?<br>***~ Planning, Executing; Process ~***<br>*~ RQ5. Knowledge and Resource Sharing ~*<br>*~ RQ6. Workforce ~*
Follow up:<br>• *Who was involved in trainings* ***(NOTE: at all levels)****?*<br>• *Who taught the training?*<br>• *How were they selected (trainers and trainees)?*<br>• *What was their motivation/incentive for involvement?*<br>• *How were the trainings conducted?*
6. If no training activities, what modality did the intervention have?
Follow up:<br>• *What modalities were used for different aspects of the project, generally?*
7. Did you make any changes to the supply chain in $\overline{\quad YEAR(S) \quad}$? If so, tell us how.<br>***~ Execution; Process~***<br>*~ Q6. Workforce ~*
Follow up:<br>• *What worked well?*<br>• *What were the challenges?*
8. Did you make any changes to data systems or their use in $\overline{\quad YEAR(S) \quad}$? If so, what kind of changes were made and how?<br>***~ Execution; Process~***<br>*~ Q3. Information Systems ~*<br>*~ Q6. Workforce ~*
9. How do you monitor the performance of immunization programs?<br>***~ Evaluation; Process~***<br>*~ RQ3. Information Systems ~*
10. How does this differ from how immunization programs were monitored in the past?<br>***~ Evaluation; Process~***<br>*~ RQ3. Information Systems ~*

Follow up:

- *Who is responsible for collecting data related to measures?*
- *How is data used?*
- *What measures are included?*
- *How often is data collected/shared?*
- *How is this information communicated on a local/regional/national level?*
- *How do you perceive the quality of data collected?*
- *If data quality is poor, is it/how is it used? If poor, what would improve the quality?*
- *Are there "independent" checks of the reported data to ensure they are valid?*
- *Would any other types of data have been helpful in managing the program?*
- *Is there an independent group that evaluates the entire program periodically? If so, please give some details regarding the composition of the group, how it is chosen, and what activities they undertake to evaluate the program?*

11. Is there any recognition for being at the top levels of coverage in the country or for making major improvements? If so, can you give some examples of recognition/incentives?

Follow up:

- *If yes, have you noticed any issues of misreporting due to the possibility of recognition/incentives?*

12. What changes have occurred in programming since $\frac{}{YEAR(S)}$?

13. What factors have helped to ensure the sustainability of the immunization program?

~ Evaluation; Process~

a. **Partnerships:** Does the program have diverse organizations invested in its success? *What does the collaboration between these diverse organizations look like?*

b. **Organizational capacity:** Were there organizational systems in place to support the program needs?

c. **Evaluation:** How do evaluation results inform future program planning, implementation, and funding?

d. **Communication:** How does the immunization program secure or maintain community support of childhood vaccines?
How does the immunization program secure or maintain awareness of childhood vaccines?
How do you define awareness?
To what extent is the immunization program resilient from rumors and misinformation regarding immunizations?
How could resilience be improved?

e. **Funding**: Does the program have sustained funding?
*How important was support of the legislature to program success (**EX. appropriating additional funding not initially requesting by the executive branch**)?*

f. **Adaptation**: How does the immunization program adapt to new evidence?

Where do you learn about new evidence?<br><br>g. **Adaptation**: How does the immunization program adapt to new information?<br><br>h. **Media:** How is vaccination portrayed and discussed by the media?<br>*What role did the media play in improved coverage?*<br>*How did the media become involved?*<br>*How was that involvement sustained?*
i. **Strategic planning:** What kind of planning is there for future resource needs?<br><br>***~ Evaluation; Process~***<br>*~ RQ5. Knowledge and Resource Sharing ~*
14. What are the perceptions of corruption in the public sector?<br><br>***~ Inner Setting ~***<br>*~ RQ2. Political Will ~*
Follow up:<br>• *Perceptions of quality of electoral processes?*<br>• *Judicial institutions?*<br>• *Anti-corruption efforts?*

SECTION D. MINISTRY DEPARTMENTS (OR LIAISONS) OF INFRASTRUCTURE, EQUIPMENT, FINANCE

Objectives:

- To understand the vaccine intervention characteristics during country-specific change points in DTP1/DTP3 coverage
- To identify key internal and external actors, stakeholders, and partnerships in the immunization program both now and in the past
- To explore decision-making process in selecting the strategies and activities in the immunization program that created change points in DTP1/DTP3 coverage
- To identify the strategies behind current immunization programs to inform current context
- To identify previous immunization activities that were key during change points in the DTP1/DTP3 curve
- To understand key barriers and facilitators of interventions in the immunization sector
- To understand current and historical investments in the immunization sector

PART D1 – POLICY

1. What are the current regulations related to expenditures in health services?

~ External Policies; Outer Setting ~
~ RQ2. Political Will ~

Follow up:

- *What are the current regulations related to vaccination in particular?*

2. How have these regulations changed since 2000?

~ External Policies; Outer Setting ~
~ RQ2. Political Will ~

Follow up:

- *What regulation did you find to be most important/effective in increasing vaccine coverage?*
- *Which supporting documents could you share with me?*
- *What role does the Ministry of Finance have in determining these rules and regulations?*
- *What role does the Ministry of Finance have in implementing the rules and regulations?*
- *What barriers exist?*
- *How are these barriers addressed?*
- *How does this differ from the past?*

3. How have decisions on types of investments in the immunization sector been made?

~ Outer Setting ~
~ RQ1b. Immunization System Context Within the Health Sector ~

Follow up:

- *How are these decisions relative to other investments in other healthcare sectors?*
- *Who determines this?*
- *What percentage/amount of funding is spent on vaccinations as opposed to other health sector areas, including disease surveillance, coverage measurement, accountability?*

PART D2 – IMMUNIZATION PROGRAM

4. Did you make any changes to the supply chain in $\overline{\quad YEAR(S) \quad}$? If so, tell us how.   ***~ Execution; Process~***   *~ Q6. Workforce ~*
5. What changes have occurred in immunization programming since $\overline{\quad YEAR(S) \quad}$?   ***~ Evaluation; Process ~***
6. What factors have maintained the sustainability of the immunization program?   ***~ Evaluation; Process~***
a. **Funding**: Does the program have sustained funding?   ***~ Evaluation; Process~***   *~ RQ7. Vaccine Policy and Policy Enforcement ~*
b. **Adaptation**: How does the immunization program adapt to new evidence?   ***~ Evaluation; Process~***   *~RQ1. Why and How? ~*   *~ RQ 3. Information Systems ~*
Probes: • *Where do you learn about new evidence?*
c. **Adaptation**: How does the immunization program adapt to new information?
Probes: • *Where do you learn about new information?*
d. **Evaluation**: How do evaluation results inform future program planning, and implementation, and funding?   ***~ Evaluation; Process~***   *~ RQ3. Information Systems ~*
Follow up: • *What kind of data drives this?*   • *Can you share any data with us?*
j. What are the perceptions of corruption in the public sector?   ***~ Inner Setting ~***   *~ RQ2. Political Will ~*
Follow up: • *Perceptions of quality of electoral processes?*   • *Judicial institutions?*   • *Anti-corruption efforts?*

**SECTION E.** PARTNER ORGANIZATIONS, SUCH AS WHO, UNICEF, CDC, USAID, ETC; NITAG, PROFESSIONAL ORGANIZATION MEMBERS<br><br>**Objectives:**<br>• To understand the vaccine intervention characteristics during country-specific change points in DTP1/DTP3 coverage<br>• To identify key internal and external actors, stakeholders, and partnerships in the immunization program both now and in the past<br>• To explore decision-making process in selecting the strategies and activities in the immunization program that created change points in DTP1/DTP3 coverage<br>• To identify the strategies behind current immunization programs to inform current context<br>• To identify previous immunization activities that were key during change points in the DTP1/DTP3 curve<br>• To understand key barriers and facilitators of interventions in the immunization sector
**PART E1 – STAKEHOLDERS**
1. What determines opportunities for career advancement for stakeholders involved in vaccination efforts?<br><br>***~ Relative Priority, Incentives and Rewards; Process ~***<br>*~ RQ2. Political Will ~*
Follow up:<br>• *Is there a way of systematically knowing partisan affiliations or who appointed them? Specifically, those involved in agencies relevant to vaccination.*
**PART E2 – POLICY**
2. What national policies do you think were important in increasing vaccination coverage during $\underset{YEAR(S)}{\rule{3cm}{0.4pt}}$?<br><br>***~ External Policies; Outer Setting~***<br>*~ RQ2. Political Will ~*<br>*~ RQ7. Vaccine Policy and Policy Enforcement ~*
Follow up:<br><br>• *How were these policies informed?*<br>• *What was the reason/rationale for creating these new policies?*<br>• *What is the policy making process, and how are new policies or recommendations disseminated and implemented once approved?*<br>• *How do they work in both vertical* ***(EX. across ministries)*** *and horizontal* ***(EX. within different levels of a particular ministry)****?*<br>• *What led to the selection of these policies?*
**PART E3 – IMMUNIZATION PROGRAM**

3. What areas of the country have seen the greatest improvement in vaccine rates?

~ Evaluation; Process ~
~ RQ3. Information Systems ~
~ RQ4. Intent/Demand for Vaccines ~

Follow up:

- *Was the project specifically tailored to this location?*
- *Was it targeted as a critical area for change?*
- *What areas saw the quickest improvement?*
- *Why do you think these areas had the greatest improvement?*

4. What areas of the country did not see significant changes?

~ Evaluation; Process~
~ RQ3. Information Systems ~
~ RQ4. Intent/Demand for Vaccines ~

Follow up:

- *Why do you think this is?* ***(EX: May have already had high coverage, may not relate to challenges)***
- *What were the challenges?*
  - *Why?*
- *What has been/might be done to mitigate this?*

5. What populations are currently the most vulnerable to low coverage?

~ Evaluation; Process~

6. What populations historically were the most vulnerable to low coverage?

~ Evaluation; Process~

Follow up:

- *How did you identify these populations as vulnerable?*
  - *Both previously and now*
- *How did you reach out to these populations?*
- *What strategy/strategies do you use to reduce inequities?*
- *What strategy did you previously use to reduce inequities?*
- *What kind of data supports this?*
- *Is this data you can share with me?*

7. How do you monitor the performance of immunization programs?

8. How does this differ from how immunization programs were monitored in the past?

Follow up:

- *Who is responsible for collecting data related to measures?*
- *How is data used?*
- *What measures are included?*

- *How often is data collected/shared?*
- *How is this information communicated on a local/regional/national level?*
- *How do you perceive the quality of data collected?*
- *If data quality is poor, is it/how is it used? If poor, what would improve the quality?*
- *Are there "independent" checks of the reported data to ensure they are valid?*
- *Would any other types of data have been helpful in managing the program?*
- *Is there an independent group that evaluates the entire program periodically? If so, please give some details regarding the composition of the group, how it is chosen, and what activities they undertake to evaluate the program?*

9. Is there any recognition for being at the top levels of coverage in the country or for making major improvements? If so, can you give some examples of recognition/incentives?

Follow up:

- *If yes, have you noticed any issues of misreporting due to the possibility of recognition/incentives?*

10. How do you measure the impact of immunization programs?

11. What factors have helped to ensure the sustainability of the immunization program?

~ Evaluation; Process~

a. **Partnerships:** Does the program have diverse organizations invested in its success?
b. **Partnerships**: What does the collaboration between these diverse organizations look like?
c. **Organizational capacity**: Were there organizational systems in place to support the program needs?
d. **Champion**: Does the program have strong champions? ***(EX: Political or advocacy support outside of the program?)***
e. **Funding**: Does the program have sustained funding?
   ii. *Follow up: How important was support of the legislature to program success (**EX. appropriating additional funding not initially requesting by the executive branch**)?*
f. **Evaluation**: How do evaluation results inform future program planning, implementation, and funding?
   iii. *Follow up: What kind of data drives this?*
   iv. *Can you share any data with us?*

g. **Media**: How is vaccination portrayed and discussed by the media?

~ Outer Setting ~
~ RQ3. Information Systems ~
~ RQ4. Intent/Demand for Vaccines ~
~ RQ5. Knowledge and Resource Sharing ~

Follow up:

- *What role did the media play in improved coverage?*
- *How did the media become involved?*
- *How was that involvement sustained?*

h. **Adaptation**: How does the immunization program adapt to new evidence?<br><br>***~ Evaluation; Process~***<br>*~RQ1. Why and How? ~*<br>*~ RQ 3. Information Systems ~*
Follow up:<br><br>• *Where do you learn about new evidence?*
i. **Adaptation**: How does the immunization program adapt to new information?
j. **Communication**: How does the immunization program secure community support or awareness of the issue of childhood vaccines?<br><br>***~ Evaluation; Process~***<br>*~ RQ3. Information Systems ~*<br>*~ RQ4. Intent/Demand for Vaccines ~*<br>*~ RQ5. Knowledge and Resource Sharing ~*<br>*~ RQ6. Workforce ~*
Follow up:<br><br>• *How do you define awareness?*<br>• *How does the immunization program maintain community support of childhood vaccines?*<br>• *How does the immunization program maintain awareness of childhood vaccines?*<br>• *To what extent is the immunization program resilient from rumors and misinformation regarding immunizations?*<br>• *How could resilience be improved?*
12. **Strategic planning**: What kind of planning is there for future resource needs?<br><br>***~ Evaluation; Process~***<br>*~ RQ5. Knowledge and Resource Sharing ~*
13. What are the perceptions of corruption in the public sector?<br>***~ Inner Setting ~***<br>*~ RQ2. Political Will ~*
Follow up:<br><br>• *Perceptions of quality of electoral processes?*<br>• *Judicial institutions?*<br>• *Anti-corruption efforts?*

SECTION G. PROVINCIAL HEALTH DIRECTOR, SURVEILLANCE, PREVENTION, AND VACCINATION PERSONNEL

Objectives:

- To understand the vaccine intervention characteristics during country-specific change points in DTP1/DTP3 coverage
- To identify key internal and external actors, stakeholders, and partnerships in the immunization program both now and in the past
- To identify the strategies behind current immunization programs to inform current context
- To identify previous immunization activities that were key during change points in the DTP1/DTP3 curve
- To understand key barriers and facilitators of interventions in the immunization sector
- To understand health system characteristics, currently and in the past
- To identify and/or confirm project district selection

PART G1 – HEALTH SYSTEM

1. Previously, we poke about general success/increase in coverage at the national level. What are some successes in the districts?
    a.

Follow up:

- *Which specific districts would you classify as exemplar districts?*
- *Why?*
- *Which districts have sustained high coverage from 2011 – 2017?*
    - *Was the project specifically tailored to this location?*
    - *Was it targeted as a critical area for change?? Why?*
    - *What areas saw the quickest improvement?*
    - *Why do you think these areas had the greatest improvement?*
- *Which areas did not experience significant change?*
- *Which populations area most vulnerable to low coverage??*
    - *How did you identify these populations as vulnerable? Both previously and now?*
    - *How did you reach out to these populations?*
    - *What strategy/strategies do you use to reduce inequities?*
    - *What kind of data supports this?*
    - *Is this data you can share with me?*

2. Here are some districts we have selected based on heterogeneity within the province (with a focus on greater populations). What do you think about these selections?

Follow up:

- *Would you make an argument for a different selected/districts to be included? Why?*

3. How many health facilities are available per 1,000 people?

PART G2 – IMMUNIZATION PROGRAM

4. What is a <u>specific intervention</u> that contributed to the increase in vaccine coverage in your province?

Follow up:

- *Some people have mentioned specific interventions/stakeholders as being critical to the improved coverage, such as* _____________

5. What was your involvement in this intervention(s) and the changes that followed? (IF NO DIRECT INVOLVEMENT IN PLANNING ACTIVITIES, SKIP TO Q9)

Follow up:

- *Were these directives from the ministry?*
- *How did you decide which changes to make?*
- *How did this intervention affect people's roles and responsibilities at different levels in the health system?*
- *How were these changes received at different levels of the health system, including parents/end-users?*

6. What were key activities in planning changes for the program?

~ Planning; Process ~
~ RQ5. Knowledge and Resource Sharing ~
~ RQ6. Workforce ~

Follow up:

- *Did you have an action plan?*
- *A theory of change or log frame?*
- *What challenges did you face in the early planning phases?*
  - *How were these challenges overcome* ***(EX: Internal to agency/external to agency)****?*

7. Prior to the intervention, what kind of piloting or testing of the intervention occurred?

~ Planning; Process~
~ RQ4. Intent/Demand for Vaccines ~
~ RQ5. Knowledge and Resource Sharing ~
~ RQ6. Workforce ~

Follow up:

- *Did the service delivery of vaccinations change?*
- *Where did this take place?*
- *What was learned in this process?*

8. What were key training activities?

~ Planning, Executing; Process ~
~ RQ5. Knowledge and Resource Sharing ~
~ RQ6. Workforce ~

Follow up:

- *If no training activities, what modality did the intervention have?*
- *If training activities, do you have the following information, and can we access it?*
  - *Who was involved in trainings* **(NOTE: at all levels)***?*
  - *Who taught the training?*
  - *How were they selected*
    - *Trainers*
    - *Trainees?*

• *What was their motivation/incentive for involvement?*<br>• *How were the trainings conducted?*<br>• *What were the expectations of the participants in training?*
9. What kind of evidence did you use to determine [changes/interventions]?<br><br>***~ Planning; Process~***<br>*~ RQ5. Knowledge and Resource Sharing ~*
Follow up:<br>*(E.g., outbreaks, surveillance data).*<br><br>• *You mentioned* ______ (DATA SOURCE NAME(S)) *. What role did this information have in the push to increase vaccine coverage?*<br>• *Can you explain how* ______ (DATA SOURCE NAME(S)) *is collected?*<br>• *How is this* ______ (DATA SOURCE NAME(S)) *integrated into other systems?*<br>• *How does the surveillance system in* ______ (COUNTRY)*. work?*<br>***~ Planning; Process~***<br>*~ RQ5. Knowledge and Resource Sharing~*
10. What do you see as critical for <u>implementation</u> of the [intervention(s)] to succeed?<br>***~ Reflecting and Evaluating; Process ~***<br>*~ Q6. Workforce ~*
Follow up:<br><br>• *When people give commands from top down, what authority do you have to make changes?*<br>• *What advice would you give to another person/region/country who was going to implement a similar program/project to yours?What are you most proud of in terms of this work?*
11. What challenges/difficulties did you encounter when <u>implementing</u> interventions?<br><br>***~ Reflecting and Evaluating; Process~***
Follow up:<br><br>• *How were these challenges overcome?*<br>○ *What contributed to these challenges?*<br>○ *How were decisions made about what to do?*<br>○ *What challenges did you encounter with any new policies made?*<br>○ *Of these challenges, which challenge was the greatest in <u>this</u> province?*<br>○ *Was the project specifically tailored to this location?*<br>• *Who contributed to this decision-making? Why?*
12. What interventions were offered through community health workers?<br><br>***~ Execution; Process~***<br>*~ RQ6. Workforce ~*
Follow up:<br><br>• *Outreach activities? Training?*<br>• *Including any kind of reminder system for people to get vaccinations?*

- *What kind of system was this? (Cellphone texts, in-person reminders (by who?), church/religious institution reminders?)*
- *How was technology a component of this system?*
- *Did you develop or already have in place a clinic-based immunization record keeping system to track immunizations administered to individuals?*

- *Tools used?*
- *How were these developed?*
- *What led to the selection of these interventions?*
- *Was there variation in different geographic regions etc. If so, why?*
- *How were/are CHWs motivated/incentivized for involvement?*
- *What are the job requirements to be a CHW?*
- *How have responsibilities shifted over time? How do you think this has impacted their performance? Their motivation?*
- *What kind of current involvement does the Neighborhood Health Committee have in current CHW programming and responsibilities? What kind of role did they have in the past?*
- *What kinds of continued training/capacity building are provided to CHWs?*

~ Beneficiary Needs and Resources; Outer Setting ~

13. What interventions were offered at various levels of the health system? To providers?

~ Execution; Process~
~ RQ3. Information Systems ~
~ RQ6. Workforce ~

Follow up:

- *How were these developed?*
- *How were they implemented? Training?*
- *Why were they implemented in the way that they were?*
- *What led to the selection of these interventions?*
- *Was there variation in different geographic regions etc. If so, why?*

14. What programs were offered to parents or soon-to-be parents?

~ Intervention Characteristics~
~ Outer Setting ~
~ RQ4. Intent/Demand for Vaccines ~

Follow up:

- *Were these tailored to different areas/cultures/religions/languages/etc.?*
- *How were these targeted specifically to mothers, fathers or alternative caretakers?*
- *How were they developed?*
- *Where did you get the idea for these interventions?*
    - *Did you use some other framework or intervention as to inform your intervention?*
- *How were they delivered?*

- *Who was engaged in delivery?*
- *What determined their involvement?*
- *What kinds of modalities were used?*
- *What kind of communication materials (if any) were utilized? Mass media? Dissemination through health centers? Other?*
- *How did you get individuals to participate in this program?*
- *What led to their selection?*
- *Could you share any materials with us?*

~ Beneficiary needs and resources ~

15. Did you make any changes to the supply chain in ______ (YEAR(S))?

Follow up:

- *What worked well?*
- *What were challenges?*

16. Are there relevant polices at the subnational/regional level? If so, what?

~ External Policies; Outer Setting ~
~ RQ2. Political Will ~
~ RQ7. Vaccine Policy and Policy Enforcement ~

Follow up:

- *If there no policies at the subnational/regional level, do all regions need to follow the national law, or can they create their own?*
- *How were these policies informed?*
- *What data were most important in setting policy?*
- *What kind of global/regional resources were utilized?*
- *How were policies implemented?*
- *What led to the selection of these policies?*

17. What policies were made at the local level?

~ External Policies; Outer Setting ~
~ RQ2. Political Will ~
~ RQ7. Vaccine Policy and Policy Enforcement ~

Follow up:

- *How were these policies informed?*
- *How were they implemented?*
- *What led to the selection of these policies?*

18. Did you make any changes to data systems in ______ (YEAR(S)) ?

~ Execution; Process~
~ Q3. Information Systems ~
~ Q6. Workforce ~

Follow up:

- *How were these changes made?*
- *Why was this change made?*
  - *Has this change had the anticipated impact* ***(EX: on coverage or otherwise)****?*
- *Did you develop or already have in place a clinic-based immunization record keeping system to track immunizations administered to individuals?*
- *Were parents responsible for keeping track of records?*
- *To what extent was technology a component of record keeping* ***(EX: computer, phone data collection)****?*

NOTE: REMINDER SYSTEM DEFINED AS A MESSAGE SENT BEFORE APPOINTMENTS TO REMIND PARENTS TO COME; RECALL SYSTEM DEFINED AS A NOTICE SENT TO PARENTS WHO FAIL TO KEEP THEIR APPOINTMENTS.

19. What changes were made to human resources in $\underline{}_{YEAR(S)}$?

~ Execution; Process~
~ Q6. Workforce ~

Follow up:

- *If so, what kind of changes were made and how?*

20. How do you monitor the performance of immunization programs?

~ Evaluation; Process ~
~ RQ3. Information Systems ~

Follow up:

- *How is this different from other health systems?*

21. How does this differ from how immunization programs were monitored in the past?

~ Evaluation; Process ~
~ RQ3. Information Systems ~

Follow up:

- *Who is responsible for collecting data related to measures?*
- *How is data used?*
- *What measures are included?*
- *How often is data collected/shared?*
- *How is this information communicated on a local/regional/national level?*
- *How do you perceive the quality of data collected?*
- *If data quality is poor, is it/how is it used? If poor, what would improve the quality?*
- *Are there "independent" checks of the reported data to ensure they are valid?*
- *Would any other types of data have been helpful in managing the program?*
- *Is there an independent group that evaluates the entire program periodically? If so, please give some details regarding the composition of the group, how it is chosen, and what activities they undertake to evaluate the program?*

22. Is there any recognition for being at the top levels of coverage in ________ (COUNTRY). or for making major improvements? If so, can you give some examples of recognition/incentives?	
Follow up:<br>• *If yes, have you noticed any issues of misreporting due to the possibility of recognition/incentives?*	
23. How do you measure the impact of immunization programs?	***~ Evaluation; Process~***<br>*~ RQ3. Information Systems ~*
24. What factors have maintained the sustainability of the immunization program?	***~ Evaluation; Process~***
Follow up:<br>• ***Partnerships:*** *Diverse organizations? Collaboration?*<br>• ***Champion:*** *Political advocacy or support?*<br>• ***Funding:*** *Sustained funding? What will happen to the program when Gavi funding is no longer present? What do you think about this planning?*<br>• ***Evaluation:*** *Informed future funding?*<br>• ***Adaptation:*** *How does new evidence and information impact the program?*	
25. **Communication**: How does the immunization program maintain community support of childhood vaccines?	***~ Evaluation; Process~***<br>*~ RQ3. Information Systems ~*<br>*~ RQ4. Intent/Demand for Vaccines ~*<br>*~ RQ5. Knowledge and Resource Sharing ~*<br>*~ RQ6. Workforce ~*
Follow up:<br>• *To what extent is the immunization program resilient from rumors and misinformation regarding immunizations?*<br>• *What kind of misinformation regarding immunizations exist?*<br>• *How could resilience be improved?*	
26. **Media**: How is vaccination portrayed and discussed by the media?	***~ Outer Setting ~***<br>*~ RQ3. Information Systems ~*<br>*~ RQ4. Intent/Demand for Vaccines ~*<br>*~ RQ5. Knowledge and Resource Sharing ~*
Follow up:<br>• *What role did the media play in improved coverage?*<br>• *How did the media become involved?*<br>• *How is that involvement sustained?*	
27. **Strategic planning:** What kind of planning is there for future resource needs?	***~ Evaluation; Process ~***<br>*~ RQ5. Knowledge and Resource Sharing ~*
28. What are the perceptions of corruption in the public sector?	***~ Inner Setting ~***<br>*~ RQ2. Political Will ~*
Follow up:<br>• *Perceptions of quality of electoral processes?*	

- *Judicial institutions?*
- *Anti-corruption efforts?*

**SECTION H.** DISTRICT HEAD, SURVEILLANCE, PREVENTION, VACCINATION PERSONNEL<br><br>**Objectives:**<br>• To understand the vaccine intervention characteristics during country-specific change points in DTP1/DTP3 coverage<br>• To identify key internal and external actors, stakeholders, and partnerships in the immunization program both now and in the past<br>• To identify the strategies behind current immunization programs to inform current context<br>• To identify previous immunization activities that were key during change points in the DTP1/DTP3 curve<br>• To understand key barriers and facilitators of interventions in the immunization sector<br>• To understand health system characteristics, currently and in the past
**PART H1 – HEALTH SYSTEM**
1. What is the general condition of the health facilities in your district? How does this vary at different levels?
2. Are vaccines available when they are needed? Are vaccines available where they are needed?
Follow up:<br>• *Are staff available when and where needed?*<br>• *Are supplies available when and where needed?* ***(EX: Syringes, other supplies?)***<br>• *Are vaccines availability tracked?*<br>o *By location?*<br>o *Quantity?*<br>o *Timing?*<br>• *How do vaccines arrive? Are they in ideal conditions?*<br>• *Where are the vaccines usually stored, and at what temperature?*<br>• *What levels or conditions prompt restocking?*<br>o *Are the minimum supply levels maintained?*<br>• *How long does it take to restock your supply of vaccines?*
3. How many clinics are available per 1,000 people?
Follow up:<br>• *What is the average distance/length of time that a person must travel to get to the clinic?*<br>• *On average, how long does someone wait to receive vaccines?*<br>• *Are there other services included in vaccine visits?*
**PART H2 – IMMUNIZATION PROGRAM**
4. What is a <u>specific intervention</u> that contributed to the increase in vaccine coverage?
5. What was your involvement in the intervention(s) and the changes that followed? *(IF NO DIRECT INVOLVEMENT, SKIP TO Q7)*
Follow up:<br>• *Were these directives from the ministry?*<br>• *How did you decide which changes to make?*

• *How did this intervention affect people's roles and responsibilities at different levels in the health system?*
6. What were key activities in <u>planning</u> changes for the program?<br><br>***~ Planning; Process ~***<br>*~ RQ5. Knowledge and Resource Sharing ~*<br>*~ RQ6. Workforce ~*
Follow up:<br><br>• *Did you have an action plan?*<br>• *A theory of change or log frame?*<br>• *What challenges did you face in the early planning phases?*<br>○ *How were these challenges overcome* ***(EX: Internal to agency/external to agency)****?*
7. What were key training activities?<br><br>***~ Planning, Executing; Process ~***<br>*~ RQ5. Knowledge and Resource Sharing ~*<br>*~ RQ6. Workforce ~*
Follow up:<br><br>• *If no training activities, what modality did the intervention have?*<br>• *If training activities, do you have the following information, and can we access it?*<br>• *Who was involved in trainings* ***(NOTE: at all levels)****?*<br>• *Who taught the training?*<br>• *How were they selected*<br>• *Trainers*<br>• *Trainees?*<br>• *What was their motivation/incentive for involvement?*<br>• *How were the trainings conducted?*<br>• *What were the expectations of the participants in training?*
8. What kind of evidence did you use to determine [changes/interventions]?<br><br>***~ Planning; Process ~***<br>*~ RQ5. Knowledge and Resource Sharing ~*
Follow up:<br><br>*(E.g., outbreaks, surveillance data).*<br><br>• *You mentioned* ________ (DATA SOURCE NAME(S)) *. What role did this information have in the push to increase vaccine coverage?*<br>• *Can you explain how* ________ (DATA SOURCE NAME(S)) *is collected?*<br>• *How is this* ________ (DATA SOURCE NAME(S)) *integrated into other systems?*<br>• *How does the surveillance system in* ________ (COUNTRY) *work?*<br>***~ Planning; Process~***<br>*~ RQ5. Knowledge and Resource Sharing~*
9. What do you see as critical for <u>implementation</u> of the [intervention(s)] to succeed?<br><br>***~ Reflecting and Evaluating; Process~***

~ Q6. Workforce ~
Probes:<br>• *What advice would you give to another person/region/country who was going to implement a similar program/project to yours?*<br>• *What are you most proud of in terms of this work?*
10. What challenges/difficulties did you encounter during <u>implementation</u> of the interventions?<br><br>***~ Reflecting and Evaluating; Process ~***
Follow up:<br>• *How were these challenges overcome?*<br>• *What contributed to these challenges?*<br>• *How were decisions made about what to do?*<br>• *Who contributed to this decision-making? Why?*<br>• *What challenges did you encounter with any new policies made?*<br>• *Of these challenges, which challenge was the greatest in this region?*<br>•
11. What areas of the district have seen the greatest improvement in vaccine rates? Can you share this data?<br><br>***~ Evaluation; Process~***<br>*~ RQ3. Information Systems ~*<br>*~ RQ4. Intent/Demand for Vaccines ~*
Follow up:<br>• *Was the project specifically tailored to this location?*<br>• *Was it targeted as a critical area for change?*<br>• *What areas saw the quickest improvement?*<br>• *Why do you think these areas had the greatest improvement?*
12. What areas did not experience significant changes?<br>***~ Evaluation; Process~***<br>*~ RQ3. Information Systems ~*<br>*~ RQ4. Intent/Demand for Vaccines ~*
Follow up:<br>• *Why do you think this is?* ***(EX: May have already had high coverage, may not relate to challenges)***<br>• *What were the challenges?*<br>○ *Why?*<br>• *What has been/might be done to mitigate this?*
13. What populations historically were the most vulnerable to low coverage? Why?<br><br>***~ Evaluation; Process~***

Follow up:

- *How did you identify these populations as vulnerable?*
    - *Both previously and now*
- *How did you reach out to these populations?*
- *What strategy/strategies do you use to reduce inequities?*
- *What strategy did you previously use to reduce inequities?*
- *What kind of data supports this?*
- *Is this data you can share with me?*

14. What interventions were offered through Community Health Workers?

~ Execution; Process ~
~ RQ6. Workforce ~

Follow up:

- *Outreach activities? Training?*
    - *Including any kind of reminder system for people to get vaccinations?*
        - ***What kind of system was this? (Cellphone texts, in-person reminders (by who?), church/religious institution reminders?)***
        - *How was technology a component of this system?*
        - *Did you develop or already have in place a clinic-based immunization record keeping system to track immunizations administered to individuals?*
- *Tools used?*
- *How were these developed?*
- *What led to the selection of these interventions?*
- *Was there variation in different geographic regions etc. If so, why?*
- *How were/are CHWs motivated/incentivized for involvement?*
- *What are the job requirements to be a CHW?*
- *How have responsibilities shifted over time? How do you think this has impacted their performance? Their motivation?*
- *What kind of current involvement does the Neighborhood Health Committee have in current CHW programming and responsibilities? What kind of role did they have in the past?*
- *What kinds of continued training/capacity building are provided to CHWs?*

~ Beneficiary Needs and Resources; Outer Setting ~

15. What interventions were offered at various levels of the health system? For providers?

~ Execution; Process~
~ RQ3. Information Systems ~
~ RQ6. Workforce ~

Follow up:

- *How were these developed?*
- *How were they implemented? Training*
- *Why were they implemented in the way that they were?*
- *What led to the selection of these interventions?*
- *Was there variation in different geographic regions etc. If so, why?*
- *What is the educational background of providers?*

16. What key interventions or programs were offered to parents or soon-to-be parents?<br>***~ Intervention Characteristics~***<br>***~ Outer Setting ~***<br>*~ RQ4. Intent/Demand for Vaccines ~*
Follow up:<br>• *Were these tailored to different areas/cultures/religions/languages/etc.?*<br>• *How were these targeted specifically to mothers, fathers or alternative caretakers?*<br>• *How were they developed?*<br>• *Where did you get the idea for these interventions?*<br>○ *Did you use some other framework or intervention to inform your intervention?*<br>• *How were they delivered?*<br>• *Who was engaged in delivery?*<br>• *What determined their involvement?*<br>• *Who determines the programming/methods?*<br>• *Who approves the programming/methods?*<br>• *Who formulates the messages?*<br>• *What kinds of modalities were used?*<br>• *What kind of communication materials (if any) were utilized? Mass media? Dissemination through health centers? Other?*<br>• *How did you get individuals to participate in this program?*<br>• *What led to their selection?*<br>• *Could you share any materials with us?*<br>*~ Beneficiary needs and resources ~*
17. Did you make any changes to the supply chain in $\underline{\qquad\qquad}_{YEAR(S)}$? If so, tell us how.<br>***~ Execution; Process ~***<br>*~ Q6. Workforce ~*
Follow up:<br>• *What worked well?*<br>*What were challenges?*
18. What strategies did you use to reduce the drop-out rate?
19. How do you monitor the performance of immunization programs?<br>***~ Evaluation; Process ~***<br>*~ RQ3. Information Systems ~*
20. How does this differ from how immunization programs were monitored in the past?<br>***~ Evaluation; Process ~***<br>*~ RQ3. Information Systems ~*
Follow up:<br>• *Who is responsible for collecting data related to measures?*<br>• *How is data used?*

- *What measures are included?*
- *How often is data collected/shared?*
- *How is this information communicated on a local/regional/national level?*
- *How do you perceive the quality of data collected?*
- *If data quality is poor, is it/how is it used? If poor, what would improve the quality?*
- *Are there "independent" checks of the reported data to ensure they are valid?*
- *Would any other types of data have been helpful in managing the program?*
- *Is there an independent group that evaluates the entire program periodically? If so, please give some details regarding the composition of the group, how it is chosen, and what activities they undertake to evaluate the program?*

21. Is there any recognition for being at the top levels of coverage in the country or for making major improvements? If so, can you give some examples of recognition/incentives?

Follow up:

- *Have you noticed any issues of misreporting due to the possibility of rewards?*

22. What factors have maintained sustainability of the immunization program?

~ Evaluation; Process~

Follow up:

- *Partnerships: Diverse organizations? Collaboration?*
- *Champion: Political advocacy or support?*
- *Funding: Sustained funding? What will happen to the program when Gavi funding is no longer present? What do you think about this planning?*
- *Evaluation: Informed future funding?*
- *Adaptation: How does new science and knowledge impact the program?*

a. **Communication**: How does the immunization program secure awareness of the issue of childhood vaccines?

~ Evaluation; Process~
~ RQ3. Information Systems ~
~ RQ4. Intent/Demand for Vaccines ~
~ RQ5. Knowledge and Resource Sharing ~
~ RQ6. Workforce ~

Follow up:

- *How does the immunization program maintain community support of childhood vaccines?*
- *To what extent is the immunization program resilient from rumors and misinformation regarding immunizations?*
- *How could resilience be improved?*

b. **Media**: How is vaccination portrayed and discussed by the media?

~ Outer Setting ~
~ RQ3. Information Systems ~
~ RQ4. Intent/Demand for Vaccines ~
~ RQ5. Knowledge and Resource Sharing ~

Follow up:<br><br>• *What role did the media play in improved coverage?*<br>• *How did the media become involved and how was that involvement sustained?*
c. **Strategic planning**: What kind of planning is there for future resource needs?<br><br>***~ Evaluation; Process ~***<br>*~ RQ5. Knowledge and Resource Sharing ~*
23. What are the perceptions of corruption in the public sector?<br>***~ Inner Setting ~***<br>*~ RQ2. Political Will ~*
Follow up:<br><br>• *Perceptions of quality of electoral processes?*<br>• *Judicial institutions?*<br>• *Anti-corruption efforts?*

SECTION I. CLINIC HEAD

PART 1 – HEALTH SYSTEM

Objectives:

- To understand the vaccine intervention characteristics during country-specific change points in DTP1/DTP3 coverage
- To identify key internal and external actors, stakeholders, and partnerships in the immunization program both now and in the past
- To identify the strategies behind current immunization programs to inform current context
- To identify previous immunization activities that were key during change points in the DTP1/DTP3 curve
- To understand key barriers and facilitators of interventions in the immunization sector
- To understand health system characteristics, currently and in the past
- To understand community norms and practices related to vaccinations

1. How would you describe the general condition of your health facility?

2. Are vaccines available when and where they are needed?

Follow up:

- *Are staff available when and where needed?*
- *Are supplies available when and where needed?* ***(EX: Syringes, other supplies)***.
- *Are vaccines availability tracked?*
  - *By location?*
  - *Quantity?*
  - *Timing?*
- *How do vaccines arrive? Are they in ideal conditions?*
- *Where are the vaccines usually stored, and at what temperature?*
- *What levels or conditions prompt restocking?*
  - *Are there minimum supply levels maintained?*

How long does it take to restock your supply of vaccine?

3. *How does [*COMMUNITY*]* view vaccines?

~ ***Decision-making process****~*
~Current factors behind immunization~
~Demand-side~

Follow up:

- *What are the main facilitators to people getting vaccines in your community?*
  - ***Examples****:*
    - *Encouragement from healthcare workers*
    - *Shame*

- *Geography/Accessibility*
- *Trust of vaccinators/healthcare workers*
- *Messaging/Increased Awareness*
- *Community expectation*
- *Seasonality*
- *Availability*

- *What are the main barriers to people getting vaccines in your community?*
  - ***Examples**:*
    - *Geography/Accessibility*
    - *Mistrust of healthcare workers*
    - *Stock outs*
    - *Cold chain*
    - *Vaccine hesitancy (ex. Side effects)*
    - *Influence of community/religious leaders*
    - *Seasonality*
    - *Wastage*
- *How are these barriers addressed?*
- *Are there groups less likely to prioritize vaccines? Who and why?*
- *Are there children who receive some infant vaccines but don't receive others? Why?*
- *How are vaccines prioritized compared to other health interventions/measures?*

4. How have outbreaks of diseases affected vaccination rates?

~Evaluation; Process~
~RQ3. Information Systems~
~RQ4. Intent/Demand for Vaccines~

5. What services do you think the government is responsible for? Compared to other government responsibilities, how important are vaccines?

~Intervention; Outer setting~

Follow up:

- *Why are they important/not important?*
- *What is the most important to [COMMUNITY]?*
- *What is the least important to [COMMUNITY]?*

6. How has this health facility's ability to provide vaccines changed over time?

Follow up:

- *What changed?*
  - ***Examples**:*
    - *Demand from community?*
    - *Supply*
      Improved systems?
    - *Training?*
    - *Government emphasis?*
- *About what year was this?*

- *What other events happened around this time?*
  - *Political*
  - *Infrastructure changes*
  - *Other health issues*
- *Can you think of any certain events that increased or decreased vaccinations?*
  - *Strikes*
  - *Extreme weather*
  - *Outbreaks? What disease? Vaccine preventable (flu, measles, mumps, rubella, pertussis)?*

PART I2 – IMMUNIZATION PROGRAM

7. What is a specific intervention that contributed to the increase in vaccine coverage?

Follow up:

- *What was your involvement in this intervention(s) and the changes that followed?*
- *How were the changes received?*
- *Who were the key decision-makers of this intervention?*

8. What key interventions or programs were offered to parents or soon-to-be parents?

~ Intervention Characteristics~
~Outer Setting ~
~ RQ4. Intent/Demand for Vaccines ~

Follow up:

- *Were these tailored to different areas/cultures/religions/languages/etc.?*
- *How were these targeted specifically to mothers, fathers or alternative caretakers?*
- *How were they developed?*
- *Where did you get the idea for these interventions?*
  - *Did you use some other framework or intervention as to inform your intervention?*
- *How were they delivered?*
- *Who was engaged in delivery?*
- *What determined their involvement?*
- *Who determines the program/methods?*
- *Who formulates the messages?*
- *What kinds of modalities were used?*
- *What kind of communication materials (if any) were utilized? Mass media? Dissemination through health centers? Other?*
- *How did you get individuals to participate in this program?*
- *What led to their selection?*
- *What led to their selection?*
- *What encouraged parents to be more engaged in their children's health?*
- *Was this specific to immunization or general health?*
- *Were these interventions offered in combination with other health interventions?*
- *What are you doing to reduce dropout rates?*
- *What kind of awareness campaigns are there?*
- *Could you share any materials with us?*

~ Beneficiary needs and resources ~

9. What interventions were offered through Community Health Workers?

~ Execution; Process ~
~ RQ6. Workforce ~

Follow up:

- *Outreach activities? Training?*
    - *Including any kind of reminder system for people to get vaccinations?*
        - ***What kind of system was this? (Cellphone texts, in-person reminders (by who?), church/religious institution reminders?)***
        - *How was technology a component of this system?*
        - *Did you develop or already have in place a clinic-based immunization record keeping system to track immunizations administered to individuals?*
- *Tools used?*
- *How were these developed?*
- *What led to the selection of these interventions?*
- *Was there variation in different geographic regions etc. If so, why?*
- *How were/are CHWs motivated/incentivized for involvement?*
- *What are the job requirements to be a CHW?*
- *How have responsibilities shifted over time? How do you think this has impacted their performance? Their motivation?*
- *Is there a correlation between incentives and outcomes?*
- *What kind of current involvement does the Neighborhood Health Committee have in current CHW programming and responsibilities? What kind of role did they have in the past?*
- *What kinds of continued training/capacity building are provided to CHWs?*

~ Beneficiary Needs and Resources; Outer Setting ~

10. What interventions were offered at various levels of the health system? To providers?

~ Execution; Process~
~ RQ3. Information Systems ~
~ RQ6. Workforce ~

Follow up:

- *How were these developed?*
- *How were they implemented? Training?*
- *Why were they implemented in the way that they were?*
- *What led to the selection of these interventions?*
- *Was there variation in different geographic regions etc. If so, why?*
- *What is the educational background of providers?*

11. How is your work monitored?

12. How do you monitor the performance of immunization programs?

~ Evaluation; Process~
~ RQ3. Information Systems ~

13. How does this differ from how immunization programs were monitored in the past?

~ Evaluation; Process~
~ RQ3. Information Systems ~

Follow up:<br><br>• *Who is responsible for collecting data related to measures?*<br>• *How is data used?*<br>• *What measures are included?*<br>• *How often is data collected/shared?*<br>• *How is this information communicated on a local/regional/national level?*<br>• *How do you perceive the quality of data collected?*<br>• *If data quality is poor, is it/how is it used? If poor, what would improve the quality?*<br>• *Are there "independent" checks of the reported data to ensure they are valid?*<br>• *Would any other types of data have been helpful in managing the program?*<br>• *Is there an independent group that evaluates the entire program periodically? If so, please give some details regarding the composition of the group, how it is chosen, and what activities they undertake to evaluate the program?*
14. Is there any recognition for being at the top levels of coverage in the country or for making major improvements? If so, can you give some examples of recognition/incentives?
Follow up:<br><br>• *Have you noticed any issues of misreporting due to the possibility of rewards?*
15. How do you measure the impact of immunization programs?
16. What was key to success when implementing interventions?<br>***Reflecting and Evaluating; Process~***<br>*~ Q6. Workforce ~*
Follow up:<br><br>• *What advice would you give to another person/region/country who was going to implement a similar program/project to yours?*<br>• *What are you most proud of in terms of this work?*
17. What challenges/difficulties did you encounter when implementing interventions?<br>***~ Reflecting and Evaluating; Process~***
Follow up:<br><br>• *How were these challenges overcome?*<br>• *What challenges did you encounter with any new policies made?*
18. What changes have occurred in programming since this intervention(s) was implemented?<br>***~ Evaluation; Process~***<br>*~ RQ3. Information Systems ~*
19. What changes have occurred in programming since $\overline{\qquad YEAR(S) \qquad}$ /these interventions were implemented?<br>***~ Evaluation; Process~***
20. What factors have maintained the sustainability of the immunization program?<br>***~ Evaluation; Process~***

Follow up:<br><br>• ***Partnerships:*** *Diverse organizations? Collaboration?*<br>• ***Champion:*** *Political advocacy or support?*<br>• ***Funding:*** *Sustained funding? What will happen to the program when Gavi funding is no longer present? What do you think about this planning?*<br>• ***Evaluation:*** *Informed future funding?*<br>• ***Adaptation:*** *How does new science and knowledge impact the program?*
d. **Communication**: How does the immunization program secure community support or awareness of the issue of childhood vaccines?<br><br>***~ Evaluation; Process ~***<br>*~ RQ3. Information Systems ~*<br>*~ RQ4. Intent/Demand for Vaccines ~*<br>*~ RQ5. Knowledge and Resource Sharing ~*<br>*~ RQ6. Workforce ~*
Follow up:<br><br>• *How do you define awareness? What is the community aware of (EX. Benefits, services offered, etc.)*<br>• *How does the immunization program maintain community support of childhood vaccines?*<br>• *To what extent is the immunization program resilient from rumors and misinformation regarding immunizations?*<br>• *How could resilience be improved?*
e. **Media**: How is vaccination portrayed and discussed by the media?<br>***~ Outer Setting ~***<br>*~ RQ3. Information Systems ~*<br>*~ RQ4. Intent/Demand for Vaccines ~*<br>*~ RQ5. Knowledge and Resource Sharing ~*
Follow up:<br><br>• *What role did the media play in improved coverage?*<br>• *How did the media become involved and how was that involvement sustained?*
f. **Strategic planning:** To what extent is there planning for future resource needs?<br><br>***~ Evaluation; Process~***<br>*~ RQ5. Knowledge and Resource Sharing ~*
21. What are the greatest barriers to sustained coverage?<br>***~ Reflection; Process~***
22. What are the perceptions of corruption in the public sector?<br>***~ Inner Setting ~***<br>*~ RQ2. Political Will ~*
Follow up:<br><br>• *Perceptions of quality of electoral processes?*<br>• *Judicial institutions?*<br>• *Anti-corruption efforts?*

SECTION J. COMMUNITY LEADERS (RELIGIOUS LEADERS, HEAD MAN, CHIEFS,

Objectives:

- To understand their role, if any, as it relates to health interventions, specifically immunization coverage
- To identify key community stakeholders who have a role in immunization programming
- To understand the cultural context and environmental context of the selected community
- To understand what drives community acceptance of immunizations
- To understand the motivations of key community stakeholders in vaccine intervention characteristics during country-specific change points in DTP1/DTP3 coverage
- To identify the strategies behind current immunization programs to inform current context
- To identify previous immunization activities — including specific interventions, delivery systems, policies, facilitators and barriers — that were key during change points in the DTP1/DTP3 curve
- To understand specific barriers and facilitators to implementation of interventions in the immunization sector

PART J1 – INTERVIEW INTRODUCTION

__Interviewer Script__: **Thank you very much for your willingness to meet with us today and share your experience and knowledge regarding the immunization program in ____________ (COUNTRY). I'd like to begin by learning about you and your role related to the immunization program.**

1. Can you tell me about your role in ____________ (THIS COMMUNITY)?

~ Individual Setting ~

Follow up:

- *How does your role relate to health care programming?*
- *How does your role relate to the immunization program?*
- *Responsibilities (past and present)*
- *What are the contributions you feel you've made in the immunization programming in your community?*
- *What has motivated you to make these contributions?*

2. Who are other community leaders/stakeholders who have had an impact on the immunization program?

~ Inner Setting ~

Follow up:

- *What role did they play?*
- *What were their key contributions?*
- *What motivated their involvement?*
- *What were their top three incentives for performing well?*
- *What are the stakeholders' party/group affiliation?*
- *Are their positions relevant to vaccinations?*

<u>Interviewer Script</u>: Now I'd like to learn about the health care services in your community, and how immunization coverage plays a part. I'd also like to hear a bit more about community attitudes towards immunizations.

3. How does *[COMMUNITY]* view vaccines?

~ Inner Setting ~
~ Evaluation; Process ~
~Decision making process; demand-side~
~current factors behind immunization~

Probes:

- *What are the main facilitators to people getting vaccines in your community]?*
    - *Encouragement from health care workers*
    - *Shame*
    - *Geography*
    - *Enforcement*
    - *Trust of vaccinators/health care workers*
    - *Messaging/Awareness*
    - *Community expectation?*
    - *Fundamental right?*
    - *Seasonality?*
- *What are the main barriers to people getting vaccines in your community?*
    - *Geography*
    - *Mistrust of health care workers*
    - *Stock out*
    - *Cold chain*
    - *Vaccine hesitancy (ex. side effects)*
    - *Influence of community/religious leaders*
    - *Seasonality*
    - *Wastage*
- *How are these barriers addressed?*
- *Are there groups less likely to prioritize vaccines? Who and why?*
- *Are there children who receive infant vaccines (ex. DTP1) but don't receive others (ex. DTP2, DTP3)? Why?*
- *How are vaccines prioritized compared to other health interventions/measures?*

4. How have outbreaks of diseases affected mandatory vaccination rates?

~ Evaluation; Process ~
~ RQ3. Information Systems ~
~ RQ4. Intent/Demand for Vaccines ~

Probes:

Did vaccination rates increase or decrease?

5. Compared to other government responsibilities - like public safety, roads and infrastructure, education – how important are vaccines in this community?

~Intervention; Outer Setting~

Probes:

- *Why are they important/not important?*
- *What is the most important to you / [COMMUNITY]?*
- *What is the least important to you / [COMMUNITY]?*
- *Who do you believe is responsible for providing vaccines?*
    - *Government?*
    - *NGOs and aid agencies?*

6. How has the health facility's ability to provide vaccines changed over time?

~Intervention; Inner setting~

Probes:

- *What changed?*
    - *Demand from community?*
    - *Supply?*
    - *Improved systems?*
    - *Training?*
    - *Governmental emphasis?*
- *About what year was this?*
    - *What other events happened around this time?*
        - *Political*
        - *Infrastructure changes*
        - *Other health issues*
- *Can you think of any certain events that increased or decreased vaccinations?*
    - *Strikes*
    - *Extreme weather*
    - *Outbreaks*
        - *What diseases?*
            - *Vaccine preventable (flu, measles, mumps, rubella, pertussis etc.)*
            - *Non-vaccine preventable?*

7. What are specific interventions/programming shifts that have happened in the last ten years?

[Intent: Identify past immunization activities and interventions; identify previous key actors in the program]

~ Evaluation; Process~

[CFIR – Intervention, Outer Setting, Inner Setting, Characteristics of Individuals, Process]

Probes:

- *What intervention had the greatest impact?*
- *How was this intervention implemented in your community?*
- *How did people react to this intervention?*
- *What were the facilitators to uptake?*
- *What were barriers to uptake?*

8. What factors have contributed to the sustainability of the immunization program?

~ Evaluation; Process~

a. **Organizational capacity:** Were there organizational systems in place to support the program/intervention needs?<br>***~ Evaluation; Process~***<br>*~ RQ1a. Why and How? ~*<br>*~ RQ1b. Networks ~*
b. **Communication:** How does the immunization program maintain community support or awareness of the issue of childhood vaccines?<br>***~ Evaluation; Process ~***<br>*~ RQ3. Information Systems ~*<br>*~ RQ4. Intent/Demand for Vaccines ~*<br>*~ RQ5. Knowledge and Resource Sharing ~*<br>*~ RQ6. Workforce ~*
Follow-up:<br><br>*To what extent is the immunization program resilient from rumors and misinformation regarding immunizations?*<br>*How could resilience be improved?*
c. **Media:** How is vaccination portrayed and discussed by the media?<br>***~ Outer Setting ~***<br>*~ RQ3. Information Systems ~*<br>*~ RQ4. Intent/Demand for Vaccines ~*<br>*~ RQ5. Knowledge and Resource Sharing ~*
Follow-up:<br><br>• *What role did the media play in improved coverage?*<br>• *How did the media become involved and how was that involvement sustained?*
d. **Strategic planning:** To what extent is there planning for future resource needs?<br><br>***~ Evaluation; Process~***<br>*~ RQ5. Knowledge and Resource Sharing ~*
8. What are the greatest risks to sustained coverage?<br><br>***~ Reflection; Process~***
9. What lessons have you learned from working on/creating the immunization program?<br><br>***~ Evaluation [IP]***

**PART IV – CONCLUSION**<br>**<u>Interviewer Script</u>: Before we end this interview, I want to ask if you have any questions, comments, or anything else you think is important for us to know.**<br>***(Allow time for questions, comments).***
1. What lessons have you learned from working on the immunization program?<br><br>***~ Evaluation; Process~***
2. During the time we were speaking, you mentioned the following people: $\frac{\qquad\qquad}{NAME(S)}$ who were/are also stakeholders in the immunization program. Can you provide us introductions to these individuals? Who else would you suggest that we speak to about the immunization program?<br><br>***~ Characteristics of Individuals ~***
3. You also mentioned the following data sources: $\frac{\qquad\qquad\qquad}{DATA\ SOURCE\ NAME(S)}$. How can I access this data? Are you able to assist me? Are there other relevant documents you think I should have? How can I access this?
**<u>Interviewer Script</u>: Our next steps in the project are to continue key informant interviews with other stakeholders, and to analyze some of the quantitative data we are collecting.**
4. What level of update/involvement would you like to have in the project moving forward?
**<u>Interviewer Script</u>: Thank you much for your time and for answering my questions today. You have provided great information that will helpful during our research. If there are no other questions or concerns, then I will turn off the recorder, and you are free to leave as you please. Thank you again.**

NOTES:

VACCINE EXEMPLARS
Focus Group Discussion – Female Community Health Volunteers (FCHV)

Objectives:
- To understand the health services characteristics, including vaccine interventions, now and in the past;
- To identify the strategies behind vaccine programs;
- To identify role and responsibilities of the FCHVs in the immunization sector
- To identify FCHV immunization intervention-related motivations
- To understand barriers and facilitators to use of immunization services
- To understand the factors behind different levels of immunization coverage in their community, in the past and present

**PART 1 – BASIC INFORMATION**<br>*Can be asked by facilitator or filled out by note taker.*	
**A01**. **Researcher/Facilitator name:**	
**A01b. Note taker(s) name(s):**	
**A02. Consents provided by all:** Y / N<br><br>Time of consents ____ : ____ am/pm<br>Number of consents: _______	
**A03.** Date (dd/mm/yy): _____ /_____ / _____	**A04a.** Discussion start time ____ : ____ am/pm<br><br>**A04b.** Discussion end time ____ : ____ am/pm
**A05.** Recorder(s) used:	
**A06.** Location:	**A07.** Demographic surveys completed: Y / N

**PART 2a – INTRODUCTION**<br>*Facilitator to read aloud the following script:*

We are gathering today because we are interested in learning what caregivers like you think about childhood vaccines in *[COMMUNITY]*. My name is ______________, and I will be leading the discussion. This is ___________, and they will be taking notes.

We are specifically interested in learning about your thoughts, opinions, experiences, and perceptions related to the vaccination programming in your community. Please answer to the best of your ability. There are no right or wrong answers. Please share your views and opinions even if they are different from others, and you may choose to not answer any questions you are not comfortable with. You will be reimbursed (X) for traveling to this discussion, and food and drinks are provided here today. This should take about an hour and a half to two hours of your time. This discussion will be audio recorded, and used for research at Emory University and Center for Molecular Dynamics, Nepal (CMDN) to understand how Nepal has achieved high vaccination coverage. We will be recording to make sure we have accurately collected all of the information told to us. Is everyone okay with this discussion, and with being recorded?

Before we start the discussion, we have a survey for everyone to fill out. It asks you a few questions about yourself, and about how you feel about vaccines.

Pass out demographic survey; wait to continue with ground rules until all have been completed and handed back to note taker.

PART 2b – GROUND RULES

Wait for all group members to respond to the previous question, and check that all consent forms have been signed. Read aloud the following script:

For our discussion today, we have some ground rules that I would like everyone to follow:

1. Respect all group members, as well as their views and opinions. It is okay to disagree, but do so respectfully.
2. Everyone deserves a chance to speak. Please do not interrupt.
3. Do not use your mobile phones during the focus group discussion.
4. Please avoid side conversations.
5. Whatever is said here today is said in confidence, and should not be discussed outside of this group.

Does everyone agree with these rules? Do you have any you think we should add?

PART 2c – OPENING

Add any suggested rules to the list of ground rules, and wait for agreement on the rules. Read the following script aloud:

Are there any other questions before we begin? If everyone is ready, we will turn on the audio recorder and start the discussion.<br><br>*Turn on recorder once everyone is ready to start; Let everyone know that you have turned on the recorder.*<br><br>Now that the recorder is turned on, is everyone okay with me recording our discussion today? To start, let's go around the room and introduce ourselves. What is your name, how did you get started in this position, and how many years have you been working in *[COMMUNITY]*? How many people do you serve in *[COMMUNITY]?*

**PART 3 – GENERAL HEALTH/HEALTH SYSTEM**<br>*Read the following script aloud:*	
I am now going to ask you some questions about health, and the health system in *[COMMUNITY].*	
Q1. Where do people in *[COMMUNITY]* receive preventative healthcare?<br><br>*[Intent: Develop understanding of the resources that are available and that are being utilized]*<br><br>*[CFIR – Inner Setting]*	*Probes:*<br>• *And health services?*<br>• *Door to door, health facility, hospital, etc.?*<br>• *Do people use health facilities/hospital services for primary health care or emergency care? Why? How often?*<br>• *What other types of health services do you use?*<br>○ *Traditional healers, family traditions/cures*<br>• *What are the different staff types (nurse, volunteer) that work in the healthcare services?*<br>• *What are these people's responsibilities?*

**PART 4 – FCHV Roles in Vaccinations**<br>*Read the following script aloud:*	
Now, I'd like to ask you all about vaccines – specifically, vaccines that are given to children under 1 year old.	
Q2.What is a FCHV's background and training?	• *What level of education do you have?*<br>• *What was your past experience in community organizations or health care before you became an FCHV?*<br>• *What kind of training do FCHVs receive?*<br>○ *Who trains FCHVs, and how often?*<br>○ *How long of a session do you have?*<br>○ *Is this verbal, do you have written manuals, visual manuals? How do FCHVs practice message delivery?*

Q3. Walk me through FCHV responsibilities in the vaccination process.<br><br>*[Intent: Identify current immunization activities and interventions; decision making process; identify key actors in the program]*<br><br>*[CFIR – Intervention, Inner Setting, Process]*	• *What vaccine promotion duties do FCHVs participate in?*<br>• *Is this part of routine immunization services, or an outreach effort?*<br>• *Who do you answer to?*<br>• *What kind of encouragement do you receive from your community/supervisor?*<br>• *How many children/families are FCHVs responsible for?*<br>• *What kind of difficulties do FCHVs have reaching out to families?*<br>• *How do FCHVs in your community reach different families?*<br>• *How far do FCHVs typically travel to reach families?*<br>• *How important is FCHV's work with vaccines relative to their other responsibilities?*<br>• *What kind of evaluation do FCHVs receive? What health indicators do FCHVs report on/are responsible for?*<br>• *What monitoring systems exist to track vaccination coverage?*<br>• *Are there any difficulties in data collection, recording, and/or submitting to the health post? What are they?*<br>• *What system do you use to track and report the number of children vaccinated?*<br>• *How have monitoring systems changed over the time you've been in your position?*<br>• *What are FCHVs' motivations for providing vaccines/increasing coverage?*<br>• *What kind of compensation do you receive for the work you are doing?*
Q4. *Are there any programs or methods that FCHVs utilize to motivate vaccination?*<br><br>*[Intent: Decision making process; current factors behind immunization]*<br><br>*[CFIR – Inner Setting]*	*Probes:*<br><br>• *Are these programs/methods national or community determined?*<br>• *Who determines the programming/methods?*<br>• *Who approves the programming/methods?*<br>• *What messages are promoted in this community regarding vaccines?*<br>• *Who formulates the messages?*<br>• *Who approves the messaging?*

	• *How are these messages communicated to community members?*
Q5. How does *[COMMUNITY]* view vaccines?<br><br>*[Intent: Decision making process; current factors behind immunization; demand-side]*<br><br>*[CFIR – Inner Setting]*	*Probes: What are the main facilitators to people getting vaccines in your community?*<br>*Examples for reference:*<br>○ *Encouragement from health care workers*<br>○ *Shame*<br>○ *Geography*<br>○ *Enforcement*<br>○ *Trust of vaccinators/health care workers*<br>○ *Messaging/Awareness*<br>○ *Community expectation?*<br>○ *Seasonality*<br>○ *Fundamental right*<br>○ *Availability*<br>*What are the main barriers to people getting vaccines in your community? Examples for reference:*<br>○ *Geography*<br>○ *Mistrust of health care workers*<br>○ *Stock out*<br>○ *Cold chain*<br>○ *Vaccine hesitancy (ex. side effects)*<br>○ *Influence of community/religious leaders*<br>○ *Seasonality*<br>○ *Wastage*<br>• *How are these barriers addressed, if at all?*<br>• *Are there groups less likely to prioritize vaccines? Who and why?*<br>• *Are there children who receive infant vaccines (ex. DTP1) but don't receive others (ex. DTP2, DTP3)? Why?*<br>• *How are vaccines prioritized compared to other health interventions?*
Q7. Are vaccines available when and where they are needed?	*Probes:*<br>• *How about staff, syringes and other supplies?*<br>• *In what conditions do vaccines arrive?*<br>• *Where are vaccines stored?*<br>○ *Where is this storage relative to your clinic/where vaccines are administered?*<br>•

Q8. How has the immunization program changed over time?<br><br>*[Intent: Identify past immunization activities and interventions; identify previous key actors in the program; identify change points]*<br><br>*[CFIR – Intervention, Outer Setting, Inner Setting, Process]*	*Probes:*<br><br>• *What changed?*<br>o *Demand from community?*<br>o *Supply?*<br>o *Improved systems?*<br>o *Training?*<br>o *Governmental emphasis?*<br>o *Increased funding*<br>• *When did this happen? About what year was this?*<br>o *What other events happened around this time?*<br>▪ *Political*<br>▪ *Infrastructure changes*<br>▪ *Other health issues*<br>• *Can you think of any certain events that increased or decreased vaccinations?*<br>o *Strikes*<br>o *Extreme weather*<br>• *How have FCHV's motivations to vaccinate changed over time?*<br>o *Has this been affected by any certain events? (**NOTE: REFERENCE BACK TO ANSWER TO PREVIOUS PROBE)***
Q9. How have outbreaks of diseases affected mandatory vaccination rates?<br><br>*[Intent: Identify past disease events that may have impacted past vaccination events; identify change points]*<br><br>*[CFIR – Intervention, Outer Setting, Inner Setting, Process]*	*Probes:*<br><br>• *Did vaccination rates increase or decrease?*
Q10. Can you think of a specific intervention that you believe contributed to increased vaccine coverage?<br><br>*[Intent: Identify past immunization activities and interventions; identify previous key actors in the program; identify change points]*<br><br>*[CFIR – Intervention, Outer Setting, Inner Setting, Characteristics of Individuals, Process]*	*Probes:*<br><br>• *How was this intervention implemented in your community?*<br>• *What kind of training was received to prepare different people for this intervention?*<br>• *What went well in the implementation process of this intervention? were the facilitators to implementation?*<br>• *What was challenging in the implementation process of this*

	intervention? What were the barriers to implementation?
Q11. What was difficult about improving vaccine coverage in this community at that time?<br><br>*[Intent: Identify past immunization activities and interventions; identify previous key actors in the program]*<br><br>*[CFIR – Intervention, Outer Setting, Inner Setting, Characteristics of Individuals, Process]*	*Probes:*<br>• *How did this affect your community?*<br>• *What was implemented to address these specific difficulties?*<br>• *Why do you think this was successful?*<br>• *How were different people motivated for this success?*

**PART 5 – POLICY**	
Q12. What, if any, vaccines do children need to receive?<br><br>*[Intent: Identify current immunization activities and interventions]*<br><br>*[CFIR – Intervention, Outer Setting, Inner Setting]*	*Probes:*<br>• *Are the requirements clear?*<br>• *What vaccinations are required for children to be allowed to go to school?*<br>• *What affect have these laws/requirements had on FCHVs' jobs?*
Q13. How have these requirements changed over time?<br><br>*[Intent: Identify past immunization activities and interventions; identify previous key actors in the program; identify change points]*<br><br>*[CFIR – Intervention, Outer Setting, Inner Setting, Process]*	*Probes:*<br>• *Why did requirements change?*<br>• *About what year/time was this?*<br>• *What other events happened around this time?*<br>• *Who worked with your community to adapt to the new regulations?*<br>• *What kind of assistance/training was received to enable you to deliver according to new requirements?*

**PART 4 – CLOSING**<br>*Read the following script aloud:*
Those are all of my questions. What other information about vaccinations do you have that you feel would be important for me to know? Do you have any other questions or comments you'd like to add before we end the discussion?<br><br>*Wait for group to answer.*

Thank you all very much for your time and for answering my questions today. You have all provided great information that will be of help during our research. If you have any other questions or concerns, please feel free to contact _____________. You can also find this information on the consent document that you signed when we started the discussion. If there are no other questions or concerns, then I will turn off the recorder, and we will give travel reimbursement. Thank you again for your help.

VACCINE EXEMPLARS
Focus Group Discussion – Community Health Workers (CHWs), Community Health Assistants (CHAs)

Objectives:
- To understand the health services characteristics, including vaccine interventions, now and in the past;
- To identify the strategies behind vaccine programs;
- To identify role and responsibilities of the CHWs/CHAs in the immunization sector
- To identify CHW/CHA immunization intervention-related motivations
- To understand barriers and facilitators to use of immunization services
- To understand the factors behind different levels of immunization coverage in their community, in the past and present

**PART 1 – BASIC INFORMATION**<br>*Can be asked by facilitator or filled out by note taker.*	
**A01**. **Researcher/Facilitator name:**	
**A01b. Note taker(s) name(s):**	
**A02. Consents provided by all:** Y / N<br><br>Time of consents ____ : ____ am/pm<br>Number of consents: _______	
**A03.** Date (dd/mm/yy): _____ /_____ / _____	**A04a.** Discussion start time ____ : ____ am/pm<br><br>**A04b.** Discussion end time ____ : ____ am/pm
**A05.** Recorder(s) used:	
**A06.** Location:	**A07.** Demographic surveys completed: Y / N

**PART 2a – INTRODUCTION**
**Interviewer script:** We are gathering today because we are interested in learning what members of your community think about childhood vaccines. My name is ______________, and I will be leading the discussion. This is ___________, and they will be taking notes.<br><br>We are specifically interested in learning about your thoughts, opinions, experiences, and perceptions related to the vaccination programming in your community. Please answer to the best of your ability. There are no right or wrong answers. Please share your views and opinions even if they are different from others, and you may choose to not answer any questions you are not comfortable with. You will be reimbursed K50 for traveling to this discussion, and snacks are provided for after the discussion. This should take about two hours of your time. This discussion will be audio recorded, and used for research at Emory University and the Zambia Emory HIV Research Project (ZEHRP) to understand how Zambia has achieved high vaccination coverage. We will be recording to make sure we have accurately collected all of the information told to us. Is everyone okay with this discussion, and with being recorded?<br><br>Before we start the discussion, we have a survey for everyone to fill out. It asks you a few questions about yourself, and about how you feel about vaccines.<br><br>*PASS OUT DEMOGRAPHIC SURVEY; WAIT TO CONTINUE WITH GROUND RULES UNTIL ALL HAVE BEEN COMPLETED AND HANDED BACK TO NOTETAKER.*
**PART 2b – GROUND RULES**<br>*WAIT FOR ALL GROUP MEMBERS TO RESPOND TO THE PREVIOUS QUESTION, AND CHECK THAT ALL CONSENT FORMS HAVE BEEN SIGNED.*
**Interviewer script:** For our discussion today, we have some ground rules that I would like everyone to follow:<br><br>6. Respect all group members, as well as their views and opinions. It is okay to disagree, but do so respectfully.<br>7. Everyone deserves a chance to speak. Please do not interrupt.<br>8. Do not use your mobile phones during the focus group discussion.<br>9. Please avoid side conversations.<br>10. Whatever is said here today is said in confidence, and should not be discussed outside of this group.<br><br>Does everyone agree with these rules? Do you have any you think we should add?
**PART 2c – OPENING**<br>*ADD ANY SUGGESTED RULES TO THE LIST OF GROUND RULES, AND WAIT FOR AREEMENT ON THE RULES.*

**Interviewer script:** Are there any other questions before we begin? If everyone is ready, we will turn on the audio recorder and start the discussion.<br><br>*TURN ON RECORDER ONCE EVERYONE IS READY TO START. LET EVERYONE KNOW THAT YOU HAVE TURNED ON THE RECORDER.*<br><br>**Interviewer script:** Now that the recorder is turned on, is everyone okay with me recording our discussion today? To start, let's go around the room and introduce ourselves. What is your name, how did you get started in this position, and how many years have you been working in *[COMMUNITY]*? How many people do you serve in *[COMMUNITY]?*	
**PART 3 – GENERAL HEALTH/HEALTH SYSTEM**	
**Interviewer script:** I am now going to ask you some questions about health, and the health system in *[COMMUNITY]*.	
Q1. Where do people in your community receive preventative health services?	*Probes:*<br>• *Anything else?*<br>• *Door to door, health facility, etc.?*<br>• *Do people use health facilities services for primary health care or emergency care? Why? How often?*<br>• *What other types of health services do you use?*<br>○ ***Ex.** Traditional healers, family traditions/cures*<br>• *What are the different staff types (**Ex.** nurse, volunteer) that work in the healthcare services?*<br>• *What are these people's responsibilities?*
**PART 4 – CHW/CHA Roles in Vaccinations**	
**Interviewer script:** Now, I'd like to ask you all about vaccines – specifically, vaccines that are given to children under 1 year old.	
Q2.What is a *[CHW's or CHA's]* background?	• *What level of education do you have?*<br>• *What was your past experience in community organizations or health care before you became a [CHW/CHA]?*<br>• *What kind of training do [CHWs/CHAs] receive related to vaccinations?*<br>○ *Who trains [CHWs/CHAs], and how often?*<br>○ *How long of a session do you have?*<br>○ *Is this verbal, do you have written materials, visual materials? How do [CHWs/CHAs] practice message delivery?*
Q3. Walk me through [CHWs/CHAs] responsibilities in the vaccination process.	• *What vaccine promotion duties do [CHWs/CHAs] participate in?*

NOTE: DO NOT NEED TO GO THROUGH EACH PROBE.	• *Is this part of routine immunization services, or an outreach effort?*<br>• *Who do you answer to?*<br>• *What kind of encouragement do you receive from your community/supervisor?*<br>• *How many children/families are [CHWs/CHAs] responsible for?*<br>• *What kind of difficulties do [CHWs/CHAs] have reaching out to families?*<br>• *How do [CHWs/CHAs] in your community reach different families?*<br>• *How far do [CHWs/CHAs] typically travel to reach families?*<br>• *How important is [CHWs/CHAs] work with vaccines relative to their other responsibilities?*<br>• *What kind of evaluation do [CHWs/CHAs] receive? What health indicators do [CHWs/CHAs] report on/are responsible for?*<br>• *What monitoring systems exist to track vaccination coverage?*<br>• *Are there any difficulties in data collection, recording, and/or submitting to the health post? What are they?*<br>• *What system do you use to track and report the number of children vaccinated?*<br>• *How have monitoring systems changed over the time you've been in your position?*<br>• *What are [CHWs'/CHAs'] motivations for providing vaccines/increasing coverage?*<br>• *How have these motivations changed over time?*<br>o *What kind of incentives do you receive for the work you are doing?*
Q4. *Are there any programs or methods that [CHWs/CHAs] utilize to improve vaccination coverage?*	*Probes:*<br>• *Are these methods national or community determined?*<br>• *Who determines the methods?*<br>• *Who approves the methods?*<br>• *What messages are promoted in this community regarding vaccines?*<br>• *Who formulates the messages?*<br>• *Who approves the messaging?*<br>• *How are these messages communicated to community members?*
Q5. How does your community view vaccines?	*Probes:*<br>• ***How are vaccines prioritized compared to other health interventions?***

	• *What are the main facilitators to people getting vaccines in your community?Anything ese?*<br>*Examples for reference:*<br>○ *Encouragement from health care workers*<br>○ *Shame*<br>○ *Geography*<br>○ *Trust of vaccinators/health care workers*<br>○ *Messaging/Awareness*<br>○ *Community expectation*<br>○ *Seasonality*<br>○ *Fundamental right*<br>○ *Availability*<br><br>• *What are the main barriers to people getting vaccines in your community? Anything else?*<br>*Examples for reference:*<br>○ *Geography*<br>○ *Mistrust of health care workers*<br>○ *Stock out*<br>○ *Cold chain*<br>○ *Vaccine hesitancy (ex. side effects)*<br>○ *Influence of community/religious leaders*<br>○ *Seasonality*<br>• *How are these barriers addressed?*<br>• *Are there groups less likely to prioritize vaccines? Who and why?*<br>• *Are there children who receive infant vaccines but don't receive others? Why?*
Q7. Are vaccines available when and where they are needed?	*Probes:*<br>• *How about staff? Other supplies (**Ex.** syringes)?*<br>• *In what conditions do vaccines arrive?*<br>• *Where are vaccines stored?*<br>○ *Where is this storage relative to your clinic/where vaccines are administered?*
Q8. How has the immunization program changed over time?	*Probes:*<br>• *What changed?What else?*<br>• ***Examples:***<br>○ *Demand from community?*<br>○ *Supply?*<br>○ *Improved systems?*<br>○ *Training?*<br>○ *Governmental emphasis?*<br>○ *Increased funding*<br>• *When did this happen? About what year was this?*

	○ *What other events happened around this time?*<br>▪ *Political*<br>▪ *Infrastructure changes (Ex. road development)*<br>▪ *Other health issues*<br>• *Can you think of any certain events that increased or decreased vaccinations? (Ex. Strikes, extreme weather)*
Q9. How have outbreaks of diseases affected the number of children getting vaccinated?	*Probes:*<br>• *Why?*
Q10. Can you think of a specific intervention that you believe contributed to increased vaccine coverage?	*Probes:*<br>• *What was done to prepare different people for this intervention?*<br>• *How was this intervention implemented in your community?*<br>• *What went well in the implementation process of this intervention?*<br>• *What was challenging in the implementation process of this intervention?*
Q11. What was difficult about improving vaccine coverage in this community?	*Probes:*<br>• *When was this?*<br>• *How did this affect your community?*<br>• *What was implemented to address these specific difficulties?*<br>• *Why do you think this was successful?*<br>• *How were different people motivated for this success?*
**PART 5 – POLICY**	
Q12. What vaccines do children need to receive?	*Probes:*<br>• *Do community members know the schedule of vaccinations?*<br>• *What vaccinations are required for children to be enrolled in school?*<br>• *What effect have these requirements had on <u>[CHWs/CHAs]</u> jobs?*<br>• *Is there a time when you need to present proof of routine vaccinations?*<br>• *Have these requirements changed?*<br>• *If yes, about what year/time was this?*<br>○ *What other events happened around this time?*<br>• *Who worked with your community to adapt to the new regulations?*

	• *What kind of assistance/training was received to enable you to deliver according to new requirements?*
**PART 4 – CLOSING**	
**Interviewer script**: Those are all of my questions. What other information about vaccinations do you have that you feel would be important for me to know? Do you have any other questions or comments you'd like to add before we end the discussion?<br><br>*WAIT FOR GROUP TO ANSWER.*<br><br>**Interviewer script:** Thank you all very much for your time and for answering my questions today. You have all provided great information that will be of help during our research. If you have any other questions or concerns, please feel free to contact Dr. William Kilembe. You can also find this information on the consent document that you signed when we started the discussion. If there are no other questions or concerns, then I will turn off the recorder, and we will give travel reimbursement; feel free to have snacks. Thank you again for your help.	

VACCINE EXEMPLARS
Focus Group Discussion – Parents/Caregivers

Note: Do not read red italics aloud

Objectives:
- To understand the vaccine intervention characteristics during country-specific change points in DTP1/DTP3 coverage
- To identify parental/caregiver motivation behind interventions during the change point within the immunization program
- To explore decision-making process of parents/caregivers in the change point
- To identify previous immunization activities - including specific interventions, delivery systems, policies, facilitators and barriers – that were key during change points in the DTP1/DTP3 curve and to understand barriers and facilitators to implementation
- To understand the current factors behind different levels of immunization coverage in their community
- To understand the factors behind different levels of immunization coverage in their community at change point

Framework Application Key:
WHAT – Solutions
HOW – Mechanisms of Change
WHY – Problem Identification

**PART 1 – BASIC INFORMATION**<br>*Can be asked by facilitator or filled out by note taker.*
**A01. Researcher/Facilitator name:**

**A01b. Note taker(s) name(s):**	
**A01c. Translator or observer:**	
**A01d. Copy belongs to:**	
**A02. Consents provided by all:** Y / N<br><br>Time of consents ____ : ____ am/pm<br>Number of consents: _______	
**A03.** Date (dd/mm/yy): _____ /_____ / _____	**A04a.** Discussion start time ____ : ____ am/pm<br><br>**A04b.** Discussion end time ____ : ____ am/pm<br><br>Time zone: _____
**A05.** Group (check all that apply):<br>☐ Mothers (under age 25)<br>☐ Mothers/Grandmothers (over age 25)<br>☐ Fathers<br>☐ Other - _____________	**A06.** Recorder(s) used:
**A07.** Location:	**A08.** Demographic surveys completed: Y / N

PART 2a – INTRODUCTION
Facilitator to read aloud the following script:

We are gathering today because we are interested in learning what caregivers like you think about childhood vaccines in *[COMMUNITY]*. My name is ______________, and I will be leading the discussion. This is ___________, and they will be taking notes.

We are specifically interested in learning about your thoughts, opinions, experiences, and perceptions related to the vaccines your child/grandchild has or has not received. Please answer to the best of your ability. There are no right or wrong answers. Please share your views and opinions even if they are different from others, and you may choose to not answer any questions you are not comfortable with. You will be reimbursed (X) for traveling to this discussion, and food and drinks are provided here today. This should take about an hour to an hour and a half of your time. This discussion will be audio recorded, and used for research at Emory University and *[LOCAL PARTNER]* to understand how *[COUNTRY]* has achieved high vaccination coverage. We will be recording to make sure we have accurately collected all of the information told to us. Is everyone okay with this discussion, and with being recorded?

Before we start the discussion, we have a survey for everyone to fill out. It asks you a few questions about yourself, and about how you feel about vaccines.

Pass out demographic survey; wait to continue with ground rules until all have been completed and handed back to note taker.

PART 2b – GROUND RULES

Wait for all group members to respond to the previous question, and check that all consent forms have been signed. Read aloud the following script:

For our discussion today, we have some ground rules that I would like everyone to follow:

11. Respect all group members, as well as their views and opinions. It is okay to disagree, but do so respectfully.
12. Everyone deserves a chance to speak. Please do not interrupt.
13. There are no right or wrong answers.
14. Please avoid side conversations.
15. Whatever is said here today is said in confidence, and should not be discussed outside of this group.

Does everyone agree with these rules? Do you have any you think we should add?

PART 2c – OPENING

Add any suggested rules to the list of ground rules, and wait for agreement on the rules. Read the following script aloud:

Are there any other questions before we begin? If everyone is ready, we will turn on the audio recorder and start the discussion.

Turn on recorder once everyone is ready to start; Let everyone know that you have turned on the recorder.

Now that the recorder is turned on, is everyone okay with me recording our discussion today? To start, let's go around the room and introduce ourselves. What is your name, how many children do you have, and what are their ages?

PART 3 – GENERAL HEALTH

Read the following script aloud:

I am now going to ask you some questions about health in general, and health in *[COMMUNITY]*.

Q1. Please tell me about how people in *[COMMUNITY]* receive healthcare and health services?<br><br>*[Intent: Develop understanding of the resources that are available and that are being utilized; health literacy; general health interventions]*	*Probes:*<br>• *Local health clinic, nurse, doctor, community health workers?*<br>• *Friends and family?*<br>• *Where do you seek healthcare?*<br>○ *Why?*<br>• *Do you go to prevent disease, or only when you are sick?*<br>○ *Why?*<br>• *When do you seek health related information?*<br>○ Why?
Q2. How does *[COMMUNITY]* help families to stay healthy?<br><br>*[Intent: Develop understanding of the resources that are available and that are being utilized; health literacy; general health interventions]*	*Probes:*<br>• *What are the health activities you take part in within [COMMUNITY]?*<br>• *What are the health activities your family members take part in within [COMMUNITY]?*<br>• *What do you believe your role is in regards to vaccinations?*

**PART 4 – VACCINES**<br>*Read the following to participants:*	
We are now going to talk about vaccines. Specifically, the vaccines your child was given before they were 2 years old.	
Q3. What has *[COMMUNITY]* been told about vaccines?<br><br>*[Intent: Health literacy; decision making process of parents; motivation]*	*Probes:*<br>• *How did you get this information?*<br>○ *Mass media*<br>○ *Verbally (family, friends, community health worker, etc.)*<br>○ *Paper form*<br>• *What do you think about this information?*<br>• *How much do you trust the source of vaccination information?*<br>○ *Why?*
Q4. What has *[COMMUNITY]* been told about the diseases vaccines prevent?<br><br>*[Intent: Health literacy; decision making process of parents; motivation]*	*Probes:*<br>• *How does your knowledge of this disease make you feel about vaccines (in general, or the vaccine that prevents the disease)*<br>• *Do you know what this disease is?*<br>• *Have you seen anyone get this disease before?*<br>• *Who told you about these diseases?*<br>• *Do people get this disease still?*

Q5. What do [*PROVIDERS*] at [*CLINIC*] tell mothers/caregivers about vaccinations?<br><br>*[Intent: Characteristics of community possibly leading to decision making process]*	*Probes:*<br>• *What vaccinations do providers talk about when you go to the clinic?*<br>• *Who else talks about vaccines?*<br>○ *Community health workers*<br>○ *Community leaders*<br>○ *Religious leader*<br>○ *Family (who)?*<br>○ *Friends, etc.?*
Q6. What affects mothers/caregivers decision to vaccinate or not to vaccinate their child?<br><br>*[Intent: Demand-side factors/motivation; decision making process]*	*Probes:*<br>• *How is this received by other community members?*<br>• *Is this needed for school?*<br>• *What are [COMMUNITY] beliefs surrounding vaccination?*<br>• *How convenient is it for caregivers to vaccinate children?*<br>• *Do you know of any leaders/movements/organizations that promote vaccination?*<br>○ *Any that oppose vaccination?*<br>• *Is it expensive to get vaccinated?*<br>• *When you go for vaccines, do you get other services or goods?*<br>○ *Examples – food, supplies for children, check-ups, etc.*
Q7. How important is it in *[COMMUNITY]* to get vaccinations for children?	*Probes:*<br>• *Why/why not?*
Q8. Tell me about a normal visit to get children vaccinated in *[COMMUNITY]*.<br><br>*[Intent: Find possible interventions in place within the community]*	*Probes:*<br>• *How far did you have to travel?*<br>○ *What kind of transportation did you use (walk, drive, bus, etc.)*<br>• *Did you have to present any documentation?*<br>• *About how long did getting your child vaccinated take?*<br>• *Were more than one of children getting vaccinated? Why/why not?*<br>• *How much time did it take to get to the vaccination site?*<br>• *How much of this time was spent waiting in line?*<br>• *How did this affect your daily routine?*<br>• *Was this a special vaccination day?*<br>○ *How did you know about this?*<br>○ *Are there any reminder systems?*<br>▪ *How do they work?*

	▪ *Do you pay attention to these reminders?*<br>▪ *When did these reminders start?*<br>○ *Who told you about this?*

**PART 4 – HISTORY AND POLICY**	
Q9. Have the opinions of caregivers/mothers in *[COMMUNITY]* changed over time?<br><br>*[Intent: Develop entire history; set up a timeline of how vaccination has changed]*	*Probes:*<br>• Why?<br>• *What changed for you?*<br>• *About what year was this?*<br>○ *What other events happened around this time?*
Q10. What requirements are there for childhood vaccines?<br><br>*[Intent: Knowledge of policy and policy implications]*	*Probes:*<br>• *How did you learn about these requirements?*<br>• *Have you ever had to present your child's vaccination record to have access to services?*<br>○ *What vaccinations are required for children to be allowed to go to school?*<br>• *How do you feel about these laws/requirements?*<br>• *What affect have these laws/requirements had on your life?*
Q11. Compared to other government responsibilities - like public safety, roads and infrastructure, education – how important are vaccines?	*Probes:*<br>• *Why are they important/not important?*<br>• *What is the most important to you / [COMMUNITY]?*<br>• *What is the least important to you / [COMMUNITY]?*<br>• *Who do you believe is responsible for providing vaccines?*<br>○ *Government?*<br>○ *NGOs and aid agencies?*

**PART 4 – CLOSING**
Read the following script aloud:

Those are all of my questions. Do you have any other questions or comments you'd like to add before we end the discussion?

Wait for group to answer.

Thank you all very much for your time and for answering my questions today. You have all provided great information that will helpful during our research. If you have any other questions or concerns, please feel free to contact ______________. You can also find this information on the consent document that you signed when we started the discussion. If there are no other questions or concerns, then I will turn off the recorder, and you are free to leave as you please. Thank you again.
