# Supplementary material for: Success in vaccination programming through community health workers: a qualitative analysis of interviews and focus group discussions from Nepal, Senegal and Zambia
Source: BMJ Open. 2024 Apr 3;14(4):e079358. doi: 10.1136/bmjopen-2023-079358 (PMC11146414; doi:10.1136/bmjopen-2023-079358)
Supplement: online supplemental file 2 [file bmjopen-2023-079358supp002.pdf]

Code	Memo
**1. Outer Setting (outside (Nepal/Senegal/ Zambia) government and the MOH)**	
1a. External policy & pressure	A broad construct that includes external strategies to spread interventions, including policy and regulations (governmental, regulatory, advisory, NITAG, ICC - or other funders or central entities), external mandates, recommendations and guidelines, pay-for-performance (RBF, PFP), collaboratives, and public or benchmark reporting. Includes global interventions.
1a1. Adapting global guidelines	Specific mention of the adaptation of guidelines, interventions, or policies **from external sources** to country-specific context - specifically, how and/or why these external guidelines were adapted. Does include mentions of RED/REC.
1b. External networks	The degree to which the organization is networked, and how it is networked. Communication and coordination with external partners and organizations.
**2. Inner Setting (Nepal/Senegal/Zambia) government, including the MOH)**	
2a. Internal strategy to the health system	Strategies for improving the health system at the national level. Includes how and why policies are developed, key decision-makers, and the overall decision-making environment. Includes mentions of health systems strengthening and health post expansion. Named policies specific to vaccinations go in 2a1.
2a1. National health policies	Mentions of specific national health policies **related to vaccinations**. This includes vaccine schedules and new vaccine and/or antigens introductions (even if the "vaccine introduction" isn't specific/named). If the specific policy name is not used, then code with the parent code.
2a2. Roles and responsibilities	Descriptions of **roles, responsibilities, shift in responsibilities over time, and supervision**. Includes description of how roles are coordinated within and between different levels of government. Includes the mentions of chain of command (i.e. official hierarchy within the system of authority that dictates who is in charge of whom, and who each key player reports to). Note - put roles related to resource allocation (i.e., "who can spend money?" in the 3d3: Budgeting code)
2b. Adaptability for implementation **(national to sub-national levels)**	The degree to which an intervention, policy, service delivery, or vaccine programming (in-country) was adapted, tailored, refined, or reinvented to meet local needs. This includes adaptations made at subnational levels to national-level policies or programs. Includes mentions of situational analysis or community diagnosis.
2c. Networks and Communication	The nature and quality of social networks and formal and informal communications within an organization - includes **communication between entities; or between individuals**. Includes coordination and collaboration **between government ministries** (Example: the Ministry of Health working with the Ministry of Education); **AND within the MOH** (Example: provincial level working with the community level), health facilities (Example: peer-to-peer learning between health facilities), etc.
2d. Implementation Climate - *do not use this parent code*	
2d1. Relative Priority	Implementer's shared perception of the importance of the implementation within the organization. Include mentions of political will.
2d2. Organizational Incentives & Rewards	Incentives such as goal-sharing awards, performance reviews, promotions, and raises in salary, and less tangible incentives such as increased stature or respect. Includes money, gifts, etc.

2d3. Goals and Feedback	The degree to which goals are clearly communicated, acted upon, and feedback is given to staff. The alignment of that feedback with goals. Reviewing evaluation of tasks; evaluation metrics.
2e. Readiness for Implementation - *do not use this parent code*	
2e1. Disease specific funding	Funding that is siloed, and **supposed to be used for one specific disease - even if that funding isn't used for its intended purposes.** Examples include Rotary funds for polio eradication; Lions Club funds for measles eradication; use of polio specific funds to pay salaries of staff that work on multiple projects.
2e2. Budgeting	General budgeting information - where resources are coming from, how they are allocated, who decides how resources are used, etc. Includes identification of funding sources and captures where the funding originated from (ex. WHO, RBF, Gavi, Ministry of Finance, Government)
2e3. Human resources	Mentions of the **availability (presence or lack of) human resources for health** - including staff at all levels, community workers, and volunteers.
2e4. Access to Knowledge for implementers	Ease of access to digestible information and knowledge about the intervention and how to incorporate it into work tasks. Includes past or present training from the government, external partners, health facility staff, or peers, as well as the application of knowledge. Only code for access to knowledge for implementors (CHWs, volunteers, health staff, media personnel) - education at the community level should be included in Public Awareness.
**3. Context**	
3a. Geographical/Environmental access to vaccines	Includes mentions of urban, rural, remote, or migrant populations. Also includes mentions of natural disasters like flooding or earthquakes. Also include explicit discussion of differences between districts/regions.
3b. Epidemiological context	**Mentions of vaccination coverage**, disease prevalence, and **trends/changes in coverage or disease prevelance**. Includes mentions of outbreaks (community, or national).
3c. Socio-economic & socio-cultural access to vaccines	Contextual factors such as norms, values, roles, or social, or economic resources that may affect (either positively or negatively) individual access to vaccines. Includes mention of access to education and literacy.
3d. Political context	Political context of Senegal as it relates to health systems or vaccine programming. Includes mentions of unrest and corruption.
3d1. Decentralization	Includes mentions of district or zone creation or restructuring.
**4. Specific interventions/Misc.**	
4a. Catalyst for change	**Interviewee's perception of key changes** that contributed to the improvement of and/or sustainability of immunization coverage. What were the key drivers for improved vaccine coverage in the country? "When did vaccine coverage improve?" "Why did vaccine coverage improve in this time period?" "Vital role…"
4b. Supply chain	Any mention of the vaccine supply chain, including stock, supply, and transportation. Includes mentions of challenges with the supply chain.
4b1. Cold chain expansion	Any explicit mention of **policy or decision-making related to the cold chain**. Includes mentions of the challenges with cold chain policy or decision-making.

4c. Frontline Workers	Mention of personnel (either paid or volunteer) working in health services that have direct contact with community members **and how their role relates to vaccination** (including supervisory or reporting capacities). Includes mentions of frontline health workers' motivations for working with the vaccination program. Includes mentions of CDS [Health Development Committee]. Code mentions of general role in "Roles and responsibilities"
4c1. Outreach services	Any work related to vaccinations that is done by frontline workers outside the location of the health facility (Examples: Child Health Week; mobile outreach sites, vaccination campaigns). Includes mention of mobilization or sensitization.
4d. Public awareness and demand for vaccines	Any mention of public knowledge, awareness, or demand for vaccines. For example: Are people aware of vaccines? Do they know the benefits? Do they know it's important? **//** Do parents intend and plan to vaccinate their kids? Do they demand vaccines from the government? Includes mentions of community level education and information dissemination.
4d1. Media	Any mentions of media in regards to vaccinations (either positive or negative). Examples include newspapers, posters, billboards, television, online portals, radio, social media. Includes media engagement with the health sector and mention of journalists working on health programs.
4d2. Community engagement	Working collaboratively with and through groups of people in a community to address issues affecting the well-being of those people. Examples of community engagement include social marketing, role modeling, training comminity leaders and natural leaders, and other similar activities.
4d3. Hesitancy	Mention of parents or the community's unwillingness to vaccinate, afraid of vaccines, unsure of getting their children vaccinated, or unsure about the effectivness/benefits of vaccines. Includes mentions of rumors and misinformation.
4e. Patient Needs and Resources	Use this code when an interviewee talks about **community members' barriers and facilitators to vaccination**. Includes social determinants of health. Patients refer to community members, beneficiaries of implementation, including children and parents of children who are being vaccinated.
4f. Addressing Equity	Strategies or approaches used to address equity and promote equitable access to vaccines. May include challenges to addressing equity, or barrier for implementation/policy. Barriers for individuals (beneficiaries, community members) should go in the 4: Context codes.
4g. Data quality	Changes in data quality over time. Includes mentions of data verification, internal and external data audits, data-quality assessments (DQAs), supervision for data collection.
4h. Data for **sub-national** decision-making	Any mention of the use of data for evidence-based decision-making at provincial/regional, district or health facilities/posts/centers, or local level. Includes discussion of using data to assess vaccination coverage.<br>(Examples: Provincial or District level: monthly or quarterly review meetings; Health facility level: review of data to determine outreach locations; Community level: action planning.)
4i. Surveillance	Mentions of past or present disease surveillance and how the information is used. Also includes mentions of how the surveillance system was established, funded, adapted, or how it operates. Includes discussion of monitoring and evaluation of surveillance data.
