# Supplementary material for: Success in vaccination programming through community health workers: a qualitative analysis of interviews and focus group discussions from Nepal, Senegal and Zambia
Source: BMJ Open. 2024 Apr 3;14(4):e079358. doi: 10.1136/bmjopen-2023-079358 (PMC11146414; doi:10.1136/bmjopen-2023-079358)
Supplement: Supplementary file 4 [file bmjopen-2023-079358.reviewer_comments.pdf]

PEER REVIEW HISTORY

BMJ Open publishes all reviews undertaken for accepted manuscripts. Reviewers are asked to complete a checklist review form (**http://bmjopen.bmj.com/site/about/resources/checklist.pdf**) and are provided with free text boxes to elaborate on their assessment. These free text comments are reproduced below.

ARTICLE DETAILS

**TITLE (PROVISIONAL)**	Success in vaccination programming through community health workers: A qualitative analysis of interviews and focus group discussions from Nepal, Senegal, and Zambia
**AUTHORS**	OGUTU, EMILY; Ellis, Anna; Hester, Kyra; Rodriguez, Katie; Sakas, Zoe; Jaishwal, Chandni; Yang, Chenmua; Dixit, Sameer; Bose, Anindya; Sarr, Moussa; Kilembe, William; Bednarczyk, Robert; Freeman, Matthew

VERSION 1 – REVIEW

**REVIEWER**	De Jong, Michelle<br>University of the Western Cape, School of Public Health
**REVIEW RETURNED**	26-Sep-2023

**GENERAL COMMENTS**	This article focuses on the role of community health workers in improving vaccine coverage specifically focusing on CHW programming organisation, motivation and trust. The article includes insights from 3 LMICs: Nepal, Senegal and Zambia. I general the article is very interesting and generally well written, especially the introduction and conclusion. The methods are good, I have just made a few small suggestions relating to the structure, and the tables included were helpful. I do however, feel that the findings and discussion sections could be significantly revised to enhance clarity, reduce repetition and overlap and to strengthen the overall argument of the article. I have provided more detailed questions and suggestions below.<br><br>Introduction<br>The introduction is clear and provides an overview of the key areas of relevance to the current study. It presents a strong motivation for the significance of the results.<br><br>Methods<br>The methods are well described, I do have a few small suggestions for restructuring this section which I believe may enhance the flow and readability. I would recommend dividing up the section on ‘Qualitative data analysis and collection’ so that Table 2 comes after the sentence “The activities are summarized in (Table 2).“ (line 141 p.6) then- after the table- I would continue with the description of thematic analysis, after which I would move Table 1 on the definition of themes.<br><br>A few methodological details could also be added such as the total number of participants/ interviews and focus groups (this is mentioned in the abstract and is in the table but could be briefly added to the written section) and if any specific method of thematic analysis was followed.

	Findings<br>I am not sure I understand the framework which was developed (described in lines 182-184). What was the purpose of the framework and how was it used? How did it differ from the code book? Am I correct in understanding that the framework was not necessarily based on the data but was developed before reading and coding the data? It sounds like it was based on previous studies on how community health workers influence community health/behaviour change? And then the framework was used to develop codes which were then deductively applied to the data? Why were the themes of organisation, trust and motivation selected over others? But later (line 193) it is mentioned that the 3 themes were derived from the data? I think this process could be explained a little more clearly and explicitly.<br><br>On lines 189-190 (p8) it is mentioned that one of the cadres of CHWs was 'more trusted'. I wasn't sure who they were more trusted than? Other health workers? Or other cadres of CHWs?<br><br>3.1 Organisation<br>It isn't really clear from the data presented, how the organisation of the CHW programmes had an influence on their effectiveness in increasing vaccine coverage? I had the impression from the previous sections that specific ways of organising CHWs were more effective than others? Perhaps that having more diversified and specialised cadres may have been an enabling factor but this does not seem to be the case as it is mentioned that the Nepal CHWs (which has only one cadre) were critical whereas in Senegal and Zambia (which have more cadres) there was 'overlapping or deficient coverage'?<br><br>From reading this section, it seems as though this first theme on organisation, is not necessarily answering the question on which factors successfully contributed to vaccine initiatives but is more intending to described the current programmes and contexts across the 3 regions? If this is the case, perhaps this could be made clearer from the beginning of the article? If not, the key enabling factors related to the organisation of CHW programmes need to be highlighted more clearly.<br><br>3.2.1 Recognition<br><br>The last quote on p13 (Lines 303-304) is not clearly linked to recognition? I'm not sure if there is more to the quote where the participant explains the reason behind wanting to work so that children will not miss vaccines?<br><br>3.2.2 Incentives<br>I think it would be useful to have a brief description of how you are conceptualising incentives, including definitions of intrinsic and extrinsic incentives and some examples. At the moment, there is quite a bit of overlap between the recognition section and the one on intrinsic incentives. I think this section could be reconsidered and potentially some of the information here could be moved to the recognition sub-theme. The term 'incentives' immediately brings to mind financial compensation, which I think could be another sub-theme and then perhaps there could be a separate sub-theme on intrinsic motivation- feelings of enjoyment or pride in

	their work by CHWs could be included here? If this reflects the data?<br><br>3.2.3<br>I was not sure how the CHW's community knowledge (line 338 p.14) was linked to capacity building? Perhaps this link could be more clearly explained? There are some important insights in this paragraph (lines 338-343) which demonstrate the important role CHWs were playing in these communities, but it isn't clear how capacity building was related to these achievements? In addition, I wasn't sure how community empowerment enabled the development and maintenance of social norms related to vaccines (lines 342-343)? This could also be more clearly explained.<br><br>The Zambia example on p. 14 (lines 349-356) could also be more clearly linked to capacity building.<br><br>3.3 Trust<br>Line 381 the acronym REC/RED should be written out in full.<br>Lines 386-388 which respondents are being referred to here? In Nepal there is only one cadre so does this statement not apply to Nepal? Which cadre was the one which was identified as the most trustworthy and why? What were they doing differently which could be learnt from?<br><br>Lines 455-457: I found this last sentence a bit confusing as it was not linked to the previous quote. I would recommend removing.<br><br>In general, the section on trust could be reviewed to try and reduce repetition, the themes of community engagement and knowledge sharing do appear interlinked but then I wonder if they should be two separate themes? The issue of knowledge sharing comes up frequently in the community engagement section? I also thought that perhaps key elements of the theme could be reiterated toward the end to sum up important insights for the reader? E.g. In terms of community engagement, it appears that it was not only frequent engagement with the community that was significant but also the ways in which CHWs engaged the community- they needed to spend time with individuals and to take into account community and social norms when sharing information in order to establish the trust of the community?<br><br>Overall in the findings, it would also be good to see more of the participants' quotes. Also, were there regional differences in levels of trust or motivation? How might these have influenced the effectiveness of the vaccine initiatives?<br><br>Discussion<br>The first sentence of the discussion does not align exactly with the stated aim of the analysis in the introduction. I think a consistent aim should be evident throughout the article.<br><br>Lines 463-464: It is not clear what is meant by expansion of CHW types and this is not covered in the organisation section of the findings.<br><br>The first paragraph of the discussion could be revised to summarise the key themes. At the moment it is a bit confusing to follow and new ideas, not presented in the findings, are presented

	here e.g. trust is described as contributing to CHW motivation which was not explained in the previous section.<br><br>Organisation<br>The issue of supervision and training was presented in the motivation and the capacity building sections of the findings, not the organisation section. The findigns and discussion should be consistent with each other.<br><br>Motivation<br>I would avoid presenting new findings in the discussion. The issue of vaccination as a human right, shifting community norms among others were not presented in the findings.<br><br>Trust<br>At times in this section and in the discussion overall, it isn't clear if the findings of this study or of other studies are presented. A clear articulation of what this study found and then what was different or similar in other studies and potential explanations for differences or surprising similarities could be commented on.<br>The issue of different cadres was not, I don't think, discussed in the section on trust. Is paragraph starting line 539 meant to be on trust still? If not, a new heading may be helpful here.<br><br>In the discussion overall, it would be helpful for the authors to comment on what can be learned from these findings in terms of what worked well in these regions- practices which contributed to the improvements in vaccine coverage- and some recommendation for other countries.<br><br>Conclusion<br>Nice conclusion!

**REVIEWER**	Crocker-Buque, Timothy<br>Faculty of Public Health and Policy, London School of Hygiene and Tropical Medicine, London, UK, Health Protection Research Unit in Immunisation
**REVIEW RETURNED**	21-Nov-2023

**GENERAL COMMENTS**	Thanks for the opportunity to review this paper, which presents an impressive piece of qualitative work looking at factors of success in vaccination programme delivery relating to community health workers in Nepal, Senegal and Zambia.<br>Overall, it is a well conducted, interesting and insightful piece of work well worthy of publication.<br>I have one major issue and a small number of minor issues that would improve the manuscript:<br>• The main issue on reviewing this manuscript is a clack of clarity around how the three domains (organization, motivation, and trust) were derived from the interviews. The interview guides, as described, focus on domains of implementation based on the CFIR and CICI frameworks, which then underwent qualitative thematic analysis, but how these domains were arrived at is unclear from the text as it currently stands. Given the scope and extent of the interviews conducted "thematic analysis" with inductive coding is an insufficiently detailed description of the methods employed to develop the three domains, which form the structure of the remainder of the paper. This is also not described in any more detail in the cited protocol. It would be difficult to repeat these methods and given how the methodology is

	described, I don't imagine another group of researchers would necessarily come up with the same domains, and it is not wholly clear how these domains relate to the frameworks used to structure the topic guides. Therefore, my primary request is that the methods of coding and developing the themes and domains is described in more detail.<br>• There are two other minor points in relation to this: Firstly, the themes that arose from the data are described in Table 1 in the methods, which makes it look as though they were determined before the analysis was undertaken. This should be placed at the start of the Findings, if there was no coding framework employed at the start of the analysis. This also applies to the description of Table 1 in lines 112-117. If the themes were determined a priori and used to code the data, then this should be clearly described and justified in the text, but this would not be considered inductive coding. This is confusing with the additional description in the Findings section (lines 181-184), where a "framework" is described, derived from other CHW frameworks, but not defined. Does this refer to the domains? Or was another framework applied to the analysis? If this was used to inform the analytic methods this should be in the methods section.<br>• Secondly Figure 1 describes 'diversification' rather than 'organization' as one of the domains – please clarify.<br>Minor points as follows:<br>• Paragraph 2.1, line 120-124: while I appreciated space is limited, it would be useful for the reader to know the basic vaccine coverage statistics for the countries and regions selected, otherwise it is not clear how good/excellent these places are, especially compared to other comparable regions. Suggest adding a summary overview here.<br>• Line 134: "piloted before use" – on whom, by whom? Suggest adding some more information here.<br>• Line 142-143: clarify whether the transcripts were translated, if so into what language and by whom?<br>• Table 2: needs a title. Also the ** next to the "community health workers" line is not described in the legend.<br>• Lines 194-196: this sentence is unclear.<br>• Table 3 is fascinating and very helpful.<br>• Line 234-239: would be interesting to tie this into the literature on street level bureaucracy in the discussion.<br>• The exploration of intrinsic and extrinsic motivation is excellent and carried nicely through to the discussion.<br>• Line 479-483: It's unclear, given the description of the analytic framework how the statement "social cohesion… was seen in the three countries" was described and evaluated using the methods described. I think this is outside the scope of this analysis.<br>• Suggest moving the sentence lines 509-513 to earlier in the paragraph (before or immediately after 493-495).<br>• Lines 516-518: this is interesting context for the Findings relating to remuneration. Could some quantification of the budgets and effects be added to make it more concrete?

VERSION 1 – AUTHOR RESPONSE

	Reviewer 1		

3	Generally, the article is very interesting and generally well written, especially the introduction and conclusion. The methods are good, I have just made a few small suggestions relating to the structure, and the tables included were helpful. I do however, feel that the findings and discussion sections could be significantly revised to enhance clarity, reduce repetition and overlap and to strengthen the overall argument of the article.	We have revised the findings and discussion section to provide more clarity	
5	Methods: I would recommend dividing up the section on 'Qualitative data analysis and collection' so that Table 2 comes after the sentence "The activities are summarized in (Table 2)." (line 141 p.6) then- after the table- I would continue with the description of thematic analysis, after which I would move Table 1 on the definition of themes.	Addressed: We have split the sections as suggested and moved table 2<br>"2.2.1. Qualitative data collection and<br>2.2.2. Data analysis"<br><br>However, we have not moved table 1 since it describes the themes in general, both inductively and deductively generated and deem fit at its current location.	Line 135<br><br>Line 164-172
6	A few methodological details could also be added such as the total number of participants/ interviews and focus groups (this is mentioned in the abstract and is in the table but could be briefly added to the written section) and if any specific method of thematic analysis was followed.	However, due to the word count limit, we have not included additional information as it already exists within the paper in Table 2	
7	Findings<br>I am not sure I understand the framework which was developed (described in lines 182-184). What was the purpose of the framework and how was it used? How did it differ from the code book?	We have added this sentence to provide more clarity "We developed the framework both deductively and inductively, withdrawing the domains of organization, motivation, and trust from previous studies, and aligning the definitions and categorizations with information from data."	Line 205-207
	Am I correct in understanding that the framework was not necessarily based on the data but was developed before reading and coding the data? It sounds like it was based on previous studies on how community health workers influence community health/behaviour change?	The broad domains of the framework were based on literature (Fig 2), and the definitions and explanations based on both literature and	Line 203-207<br><br>Line 211-212

		data, Table 1. We have clarified the text.	Line 121-122
	And then the framework was used to develop codes which were then deductively applied to the data?	Addressed:<br><br>“Themes around 1) organization, 2) motivation, and 3) trust were derived from the literature and aligned with data ...”	Line 216-218
	Why were the themes of organization, trust and motivation selected over others?	The themes of organization, motivation and trust highly aligned with the data and that was the basis of selecting them over the others	
	But later (line 193) it is mentioned that the 3 themes were derived from the data? I think this process could be explained a little more clearly and explicitly.	We have added “…and aligned with literature…”	Line 217-218
8	On lines 189-190 (p8) it is mentioned that one of the cadres of CHWs was ‘more trusted’. I wasn’t sure who they were more trusted than? Other health workers? Or other cadres of CHWs?	Addressed	Line 214
9	Organisation<br>It isn’t really clear from the data presented, how the organization of the CHW programmes had an influence on their effectiveness in increasing vaccine coverage? I had the impression from the previous sections that specific ways of organizing CHWs were more effective than others? Perhaps that having more diversified and specialized cadres may have been an enabling factor, but this does not seem to be the case as it is mentioned that the Nepal CHWs (which has only one cadre) were critical whereas in Senegal and Zambia (which have more cadres) there was ‘overlapping or deficient coverage’?	This is a synthesis of data from three countries, and the discussions are based on the three countries. Due to the word limit, we could not discuss each cadre individually. All the cadres discussed contributed to success in vaccination, and in Nepal, FCHV were highly mentioned to be the major contributors to success in vaccination.	
10	From reading this section, it seems as though this first theme on organization, is not necessarily answering the question on which factors successfully contributed to vaccine initiatives but is more intending to described the current programmes and contexts across the 3 regions? If this is the case, perhaps this could be made clearer from the beginning of the article? If not,	CHWs organization was one of the factors that contributed to success in vaccination.<br>Although CHW could be part of the general description of the program, from our analysis, their organization contributed to successful vaccination	Line 224-241

	the key enabling factors related to the organization of CHW programmes need to be highlighted more clearly.	programming as described in the text	
11	3.2.1 Recognition<br>The last quote on p13 (Lines 303-304) is not clearly linked to recognition? I'm not sure if there is more to the quote where the participant explains the reason behind wanting to work so that children will not miss vaccines?	Addressed: Included additional quote "We celebrated the immunization month at [location] last time"	Line 329-330
12	3.2.2 Incentives<br>I think it would be useful to have a brief description of how you are conceptualizing incentives, including definitions of intrinsic and extrinsic incentives and some examples. At the moment, there is quite a bit of overlap between the recognition section and the one on intrinsic incentives. I think this section could be reconsidered and potentially some of the information here could be moved to the recognition sub-theme.?	Addressed. We have moved the information on intrinsic motivation to recognition "In Nepal and Senegal, CHWs expressed their motivation from the satisfaction they derived from belonging to their communities. CHWs highly respected their communities, and they served voluntarily because of the mutual respect between community and CHW. Seeing children in their communities protected from diseases and growing up healthy was additional motivation. FCHVs had pride and expressed happiness if all the children within their catchment areas were fully vaccinated. A FCHV in Nepal stated that "they worked for the good of the community."	Line 331-336
13	The term 'incentives' immediately brings to mind financial compensation, which I think could be another sub-theme and then perhaps there could be a separate sub-theme on intrinsic motivation-feelings of enjoyment or pride in their work by CHWs could be included here? If this reflects the data	Addressed. We have reference table 1 which has the definitions and examples of both intrinsic and extrinsic incentives.<br><br>Examples of extrinsic incentives are included "…tangible extrinsic resources - including money, food, bicycles, uniforms, stationary, childcare, and harvesting assistance…"	Line 338<br><br>Line 339-340

14	I was not sure how the CHW's community knowledge (line 338 p.14) was linked to capacity building? Perhaps this link could be more clearly explained?	We have clarified the link between capacity building and community knowledge. "In all countries, CHW utilized their community knowledge through identification of community needs around immunization, which informed decision-making, and ultimately contributed to planning at higher levels."	Line 364-367
15	There are some important insights in this paragraph (lines 338-343) which demonstrate the important role CHWs were playing in these communities, but it isn't clear how capacity building was related to these achievements?	We have added more information to provide clarity: "The frequent interactions between CHWs and community members through community dialogues and education, and encouragement of knowledge sharing between the community members themselves enhanced community empowerment, enabling development and maintenance of social norms related to vaccination."	Line 369-372
16	In addition, I wasn't sure how community empowerment enabled the development and maintenance of social norms related to vaccines (lines 342-343)? This could also be more clearly explained.	Addressed: Added more information.<br><br>"The frequent interactions between CHWs and community members through community dialogues and education, and encouragement of knowledge sharing between the community members themselves enhanced community empowerment, enabling development and maintenance of social norms related to vaccination."	Line 369-372
17	The Zambia example on p. 14 (lines 349-356) could also be more clearly linked to capacity building.	Addressed.<br><br>"Prior to taking up their roles, NHCs received training, which enhanced their capacity to perform their duties."	Line 380-381

18	3.3 Trust<br>Line 381 the acronym REC/RED should be written out in full.	REC/RED is defined earlier in the text	Line 307-308
19	Lines 386-388 which respondents are being referred to here?	Changed respondents to mothers.	Line 417
	In Nepal there is only one cadre so does this statement not apply to Nepal?	This statement also applies to Nepal. The focus of this paper was on CHWs who played major role in immunization. In Nepal FCHVs played this role	
	Which cadre was the one which was identified as the most trustworthy and why?	Table 3 provides more information on different CHW cadres. We are limited to providing more information on specific CHW cadres due to the word limit	Line 245-248
	What were they doing differently which could be learnt from?	Addressed: “they had the most investment in the health of the mothers and their children”	Line 418-419
20	Lines 455-457: I found this last sentence a bit confusing as it was not linked to the previous quote. I would recommend removing.	We have deleted it as suggested.	
21	In general, the section on trust could be reviewed to try and reduce repetition, the themes of community engagement and knowledge sharing do appear interlinked but then I wonder if they should be two separate themes? The issue of knowledge sharing comes up frequently in the community engagement section?	We have reviewed and combined community engagement and knowledge sharing.	Line 331-464
	I also thought that perhaps key elements of the theme could be reiterated toward the end to sum up important insights for the reader? E.g. In terms of community engagement, it appears that it was not only frequent engagement with the community that was significant but also the ways in which CHWs engaged the community- they needed to spend time with individuals and to take into account community and social norms when sharing information in order to establish the trust of the community?	We have added a summary “The engagement of CHWs and their communities and the frequent interactions CHWs had with their communities, especially with mothers, facilitated the understanding of the social and cultural norms which facilitated knowledge sharing and enhanced trust between the CHWs and the communities they served and the information they shared.”	Line 478-481
22	Overall in the findings, it would also be good to see more of the participants’ quotes. Also, were	Due to the word limit, we could not provide many quotes within	

	there regional differences in levels of trust or motivation? How might these have influenced the effectiveness of the vaccine initiatives?	the manuscript. More quotes can be on found on our Open Science Framework website. We have added the link in the text.	
23	Discussion<br>The first sentence of the discussion does not align exactly with the stated aim of the analysis in the introduction. I think a consistent aim should be evident throughout the article.	Addressed. Edited the sentence to read "We conducted a qualitative study to assess factors of the CHW programs that contributed to success in implementation of vaccination initiatives in three countries between 2000 and 2019 - Nepal, Senegal and Zambia"	Line 538-540
24	Lines 463-464: It is not clear what is meant by expansion of CHW types and this is not covered in the organization section of the findings.	Addressed: Changed types to cadres	Line 543
25	The first paragraph of the discussion could be revised to summarize the key themes. At the moment it is a bit confusing to follow and new ideas, not presented in the findings, are presented here e.g. trust is described as contributing to CHW motivation which was not explained in the previous section.	We have summarized and reviewed the first paragraph to include the key themes "organization, motivation and trust" and how they contributed to improved vaccination programming.<br><br>We have changed the sentence that appeared as introducing new ideas to "Trust between communities, healthcare workers, and CHWs contributed to CHWs' commitment to their work-- resulting in the improved implementation of vaccination programming. Our findings are policy relevant for other countries looking to expand vaccination through CHW platforms; however, those looking to apply these findings should be mindful that adaptation to local context is crucial"	Line 538-548
26	Organization<br>The issue of supervision and training was presented in the motivation and the capacity building sections of the findings, not the organization section. The findings and discussion	We have reviewed for consistency and made appropriate changes. We, however, discussed supervision and training together with	

	should be consistent with each other.	organization as this clearly shows the link between them.	
27	Motivation<br>I would avoid presenting new findings in the discussion. The issue of vaccination as a human right, shifting community norms among others were not presented in the findings.	Vaccination as a human right was noted as part of the main study, and was a motivating factor based on previous literature and we have included the citation.	Line 566
28	Trust<br>At times in this section and in the discussion overall, it isn't clear if the findings of this study or of other studies are presented. A clear articulation of what this study found and then what was different or similar in other studies and potential explanations for differences or surprising similarities could be commented on.<br>The issue of different cadres was not, I don't think, discussed in the section on trust. Is paragraph starting line 539 meant to be on trust still? If not, a new heading may be helpful here.	This paragraph links the domain of trust with existing literature, no changes have been made.	
29	In the discussion overall, it would be helpful for the authors to comment on what can be learned from these findings in terms of what worked well in these regions- practices which contributed to the improvements in vaccine coverage- and some recommendation for other countries.	This is addressed in the last paragraph of the discussion and within the discussion section.<br><br>Example "This study, however, pointed out insufficiency in CHWs supervision, like findings from other studies within similar contexts[4, 40]."<br><br>Recommendation: "Since CHWs in middle and low-income countries are not healthcare professionals, increased supportive supervision may increase and enhance retention of knowledge, thus increasing their confidence"<br><br>"In Nepal, Senegal, and Zambia, CHWs were part of the communities they served, increasing their sense of belonging and trust, therefore	Line 629-641<br><br>Line 551-553<br><br>Line553-555

		increasing their motivation to dispense their duties."<br><br>"Nepal, Senegal and Zambia provide incentives to CHWs; however, this is incommensurate with their work[23]."<br><br>Recommendation: "Standardizing CHWs compensation within different settings may be of value, which could be done through governments setting aside a funding stream dedicated to CHWs and considering other sources of funding to supplement existing sources. These strategies may reduce the financial challenges experienced by the CHWs within these countries."<br><br>"Strategic approaches that allow for expanding CHW cadres are necessary to bridge the gap in human resources, and provide vaccination services to disadvantaged populations [56]."	Line 571-573<br><br>Line 583-584<br><br>Line 606-610

			Line 626-628
30	Conclusion<br>Nice conclusion!	We appreciate this feedback	
	Reviewer 2		
31	The main issue on reviewing this manuscript is a lack of clarity around how the three domains (organization, motivation, and trust) were derived from the interviews. The interview guides, as described, focus on domains of implementation based on the CFIR and CICI frameworks, which then underwent qualitative thematic analysis, but how these domains were arrived at is unclear from the text as it currently stands. Given the scope and extent of the interviews conducted “thematic analysis” with inductive coding is an insufficiently detailed description of the methods employed to develop the three domains, which form the structure of the remainder of the paper. This is also not described in any more detail in the cited protocol. It would be difficult to repeat these methods and given how the methodology is described, I don’t imagine another group of researchers would necessarily come up with the same domains, and it is not wholly clear how	Addressed<br><br>“We applied thematic analysis to identify the contribution of CHWs in the success of childhood immunization in Nepal, Senegal, and Zambia. Emory researchers developed codebook inductively using emerging themes, and deductively using themes from CHW frameworks from previous studies, coded the transcripts, and analyzed data using MaxQDA2020 software (Berlin, Germany). The coding team met frequently, discussed emerging themes, and clarified discrepancies within the codes. The researchers then	Line 164-162

	these domains relate to the frameworks used to structure the topic guides. Therefore, my primary request is that the methods of coding and developing the themes and domains is described in more detail.	categorized the themes within the broader domains of organization, motivation, and trust (Table 1). Topic guides and codebooks used for data collection and analysis are attached in the appendix 1"	
32	Firstly, the themes that arose from the data are described in Table 1 in the methods, which makes it look as though they were determined before the analysis was undertaken. This should be placed at the start of the Findings, if there was no coding framework employed at the start of the analysis. This also applies to the description of Table 1 in lines 112-117. If the themes were determined a priori and used to code the data, then this should be clearly described and justified in the text, but this would not be considered inductive coding. This is confusing with the additional description in the Findings section (lines 181-184), where a "framework" is described, derived from other CHW frameworks, but not defined. Does this refer to the domains? Or was another framework applied to the analysis? If this was used to inform the analytic methods this should be in the methods section.	We have added more information to clarify coding and analysis process included above	Line 164-172
33	Secondly Figure 1 describes 'diversification' rather than 'organization' as one of the domains – please clarify.	Addressed: changed to organization	Figure 2
34	Paragraph 2.1, line 120-124: while I appreciated space is limited, it would be useful for the reader to know the basic vaccine coverage statistics for the countries and regions selected, otherwise it is not clear how good/excellent these places are, especially compared to other comparable regions. Suggest adding a summary overview here.	We have added figure 1: DPT3 coverage of Nepal, and Senegal, and Zambia from 2000 to 2018; WUENIC data. We have also included information<br>"Figure 1 provides snapshot of coverage estimates for Nepal, Senegal, and Zambia."	Line126

35	Line 134: “piloted before use” – on whom, by whom? Suggest adding some more information here.	Addressed: piloted by the research assistants among themselves	Ln 140-141
36	Line 142-143: clarify whether the transcripts were translated, if so into what language and by whom?	Addressed: A sentence has been added: Transcripts were translated to English manually or by using Google Translate (for French), and verified by fluent bilingual speaker	Line 148-149
37	Table 2: needs a title. Also the ** next to the “community health workers” line is not described in the legend.	Title is now included.<br><br>Added description of asterisk * which was an oversight.<br><br>Asterisk** definition now included	Line 157-158<br><br>Line 160<br><br>Line 155
38	Lines 194-196: this sentence is unclear.	We have not made any changes since this is a summary of the 3 domains and how they connected and contributed to success in vaccination	
39	Table 3 is fascinating and very helpful.	We appreciate this feedback	
40	Line 234-239: would be interesting to tie this into the literature on street level bureaucracy in the discussion.	This is integrated in the discussion around trust and community ownership.	Line 612-621
41	The exploration of intrinsic and extrinsic motivation is excellent and carried nicely through to the discussion.	We appreciate this feedback	
42	Line 479-483: It’s unclear, given the description of the analytic framework how the statement “social cohesion… was seen in the three countries” was described and evaluated using the methods described. I think this is outside the scope of this analysis.	We included social cohesion, based on it’s definition as seen through extrinsic motivation. The word limit did not allow us to provide more details of how it linked to motivation	
43	Suggest moving the sentence lines 509-513 to earlier in the paragraph (before or immediately after 493-495).	We appreciate the suggestion; however, we have not made changes since line 509-513 is specific to renumeration and the	

		preceding discussion is on renumeration.	
44	Lines 516-518: this is interesting context for the Findings relating to remuneration. Could some quantification of the budgets and effects be added to make it more concrete?	Addressed:<br><br>“Costing of CHW programming, both direct and indirect costs, and considering the difference in contexts and the funding sources have been found to be essential for an effective CHW programming [51].”	Line 598-600

VERSION 2 – REVIEW

**REVIEWER**	De Jong, Michelle<br>University of the Western Cape, School of Public Health
**REVIEW RETURNED**	09-Feb-2024

**GENERAL COMMENTS**	Thank you for the opportunity to review this article again. I appreciate the authors efforts to address some of the suggestions I provided in my previous review. While additional details were added, I still felt that the thematic analysis process could be more clearly described, perhaps the code book would help. A few additional suggestions could be considered.<br><br>Line 125: Table 1<br>No studies are cited in the section on organization- References could be added to this section or the process of developing themes could be more clearly explained.<br><br>Line 147: The specific languages which were used could be included here<br><br>Line 190: I didn’t see the code book in Appendix 1, this could be included.

VERSION 2 – AUTHOR RESPONSE

	Reviewer 1		
3	Line 125: Table 1<br>No studies are cited in the section on organization- References could be added to this section or the process of developing themes could be more clearly explained.	Citations added: Expansion of CHWs with different cadres, and roles and responsibilities; may be based on the scope of work, reimbursement, qualifications, language, education level, geographic region, and their distinct roles and responsibilities(19, 20)	Table 1

5	Line 147: The specific languages which were used could be included here	Added: KIIs and FGDs were conducted in Nepali in Nepal, French in Senegal, and Bemba in Zambia	
6	Line 190: I didn’t see the code book in Appendix 1, this could be included.	Added: Codebook, Appendix 3	
